# Darizmetinib (HRX215): A Promising 1st-in-Class Liver Regenerating Drug in Phase 1b/2a Clinical Development, Targeting the Stress Signaling Protein Kinase MKK4

**DOI:** 10.1021/acs.jmedchem.6c00689

**Published:** 2026-05-06

**Authors:** Wolfgang Albrecht, Roland Selig, Bent Pfaffenrot, Philip Kloevekorn, Michael Juchum, Stefan Zwirner, Sabrina Klotz, Lars Zender, Stefan Laufer

**Affiliations:** † HepaRegeniX GmbH, Tuebingen 72072, Germany; ‡ Department of Pharmaceutical Chemistry, University of Tuebingen, Tuebingen 72076, Germany; § Department of Medical Oncology and Pneumology (Internal Medicine VIII), University Hospital Tuebingen, Tuebingen 72076, Germany; ∥ iFIT Cluster of Excellence (EXC 2180) ‘‘Image-guided and Functionally Instructed Tumor Therapies’’, University of Tuebingen, Tuebingen 72076, Germany; ⊥ German Cancer Research Consortium (DKTK), German Cancer Research Center (DKFZ), Heidelberg 69120, Germany; # Tuebingen Center for Academic Drug Discovery & Development (TueCAD2), Tuebingen 72076, Germany

## Abstract

Mitogen-activated protein kinase kinase 4 (MKK4), a MAP2 kinase that activates c-Jun N-terminal kinase (JNK) and p38 mitogen-activated protein kinase, is a key kinase of the stress-activated protein kinase (SAPK)/mitogen-activated protein kinase (MAPK) signaling network. Inhibition of MKK4 represents a novel therapeutic approach by leveraging a rerouting mechanism within the signaling network, predominantly via MKK7 and JNK1, to modulate the regenerative capacity of hepatocytes. In this study, we describe the discovery of darizmetinib (HRX215), a first-in-class MKK4 inhibitor currently in clinical development. Darizmetinib was derived from a known BRaf inhibitor, and through extensive structure–activity relationship (SAR) studies, we successfully engineered potency and selectivity for MKK4 while eliminating the original BRaf on-target activity. Preclinical proof-of-concept studies, along with *in vivo* evaluations of different lead candidates, identified darizmetinib, demonstrating dose-dependent efficacy across various disease-relevant pharmacological models.

## Graphical abstract

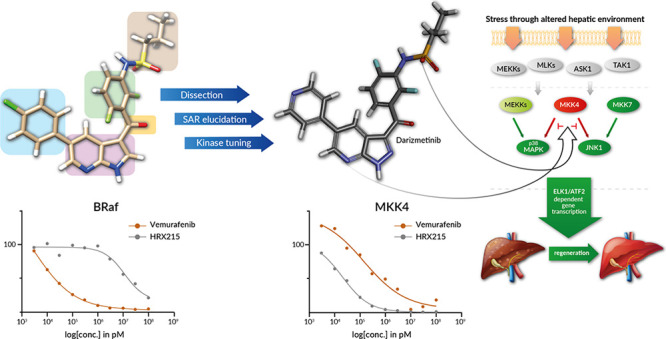

## Introduction

Liver diseases represent a major and growing global health problem, accounting for more than two million deaths annually, a number predicted to double within the next 20 years.^1^ Liver diseases, whether acute or chronic, are marked by a progressive decline in liver function and structural deterioration of liver tissue. This deterioration is driven by external factors such as high-fat diets, alcohol abuse, toxins, and infections as well as internal factors such as autoimmune disorders, genetic predispositions, and metabolic diseases. While healthy livers possess remarkable regenerative potential, damage-associated changes in the hepatic microenvironment of injured livers significantly reduce hepatocyte regeneration. In this progressive liver condition, lipid accumulation triggers inflammation and cell damage, leading to fibrosis and eventually cirrhosis. Recently, the U.S. Food and Drug Administration granted accelerated approval to Rezdiffra (resmetirom),^2^ the first drug for the treatment of metabolic dysfunction-associated steatohepatitis (MASH, formerly NASH). The complex pathophysiology of the condition and the need for long-term safety have until now hindered drug discovery efforts. The recent approval of Rezdiffra, an oral thyroid hormone receptor-β (THR-β) agonist that improves lipid metabolism and mitochondrial function in liver cells, represents an important advance in the treatment of MASH. Nevertheless, effective therapies for acute liver failure and advanced chronic liver disease remain unavailable, underscoring a critical unmet medical need. In this context, recent studies have identified mitogen-activated protein kinase 4 (MKK4) as a key regulator of hepatocyte regeneration.

In 2013, Wuestefeld et al. published the discovery of MKK4 as a key regulatory protein for liver regeneration.^3^ For details of the unique, unbiased *in vivo* RNAi-screen at that time, reference is made to the original publication. The MAP2 kinase MKK4 belongs to the 3-tiered stress signaling pathway and, after being activated through MAP3 kinases, contributes together with MKK7 to the activation of the JNK-isoforms. MKK4 is also involved in the p38 MAP kinase signaling pathway, which, however, is mainly activated by the MAP2 kinases MKK3 and MKK6. The stress signaling pathway, although ubiquitously expressed and highly conserved, exhibits cell- and context-specific features.^4,5^ Wuestefeld et al. nicely demonstrated that in the regenerating liver, the shRNA-mediated downregulation of MKK4, which led to a residual protein expression of approximately 10%, did not result in an inhibition or reduced activity of JNK-signaling. Quite the contrary, a high activity of MKK7/JNK1 signaling and activation of transcription factors ATF2 and ELK1 is apparently essential for unlocking the liver regeneration capacity (Figure 1). The genetic suppression of either MKK7 with MKK4 or JNK1 with MKK4 completely abolished the pro-regenerative effect of specific MKK4 downregulation.^3^

**Figure 1:**
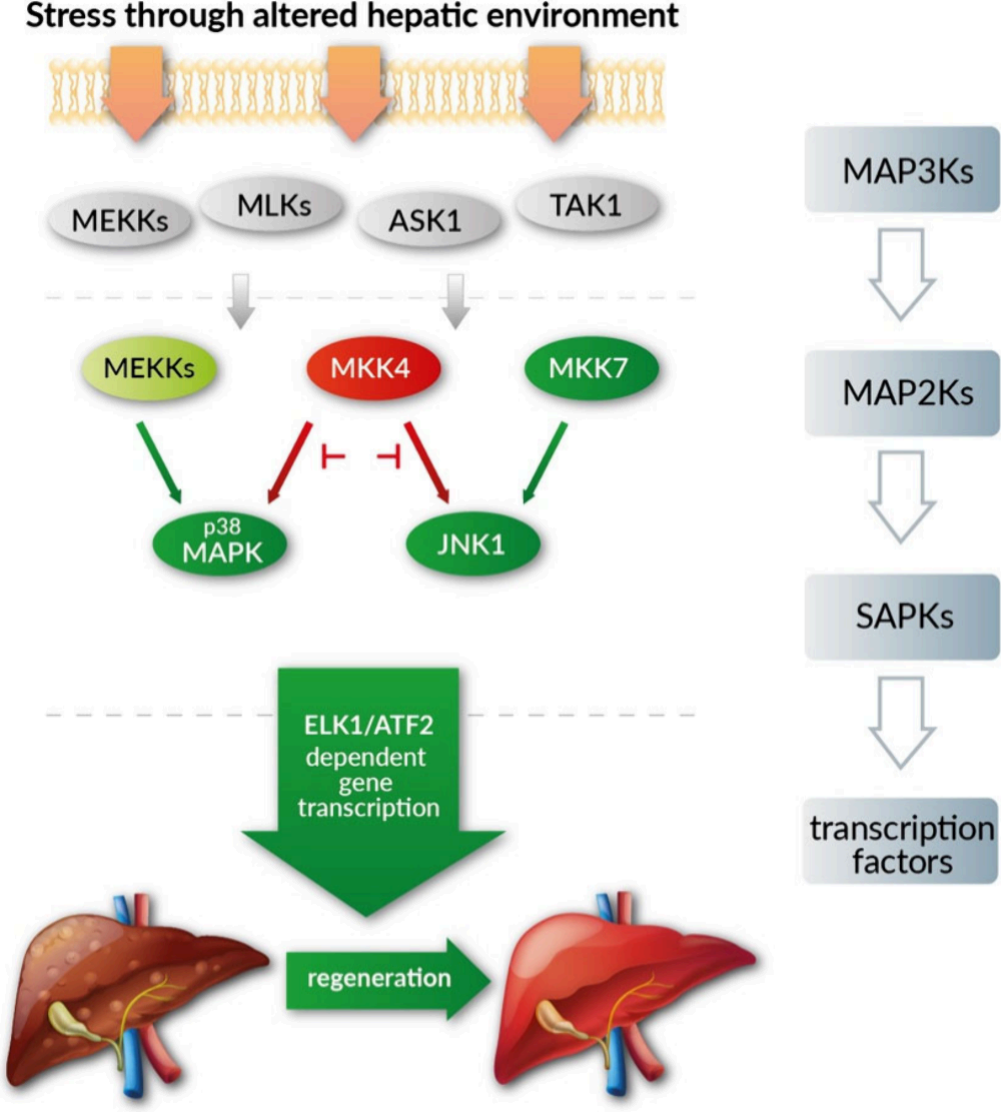
Stress signaling pathway, activated by liver injury. Genetic knockdown or pharmacologic inhibition of MKK4 promotes MKK7-mediated activation of JNK1 leading to the activation of pro-regenerative gene transcription.

In mice, after 2/3 hepatectomy, the genetic suppression of MKK4 led to faster cell-cycle entry and progression, as demonstrated by the time-dependent expression of cyclin E and cyclin A. MKK4-suppression also stabilized hepatocytes in mouse livers after the induction of acute liver injury by single CCl_4_-administration and reduced the development of liver fibrosis in chronic liver disease. These data strongly suggest, that MKK4 is a promising pharmacological target for promoting liver regeneration and treatment of liver diseases. Prior to the discovery of its potential in the liver regeneration space, MKK4 has been described as a driver of metastatic transformation in human prostate cancer^6^ and a first search toward small-molecule inhibitors was based on a thermal shift assay based screen of a small library but only a few isoflavones such as geraldol or alizarin with IC_50_-values in the range of 1 μM were identified.^6^ As potential anti-inflammatory drugs upstream of JNK, Bayer disclosed pyrimidino­[4,5*-b*]­indole derivatives as dual inhibitors of MKK4 and MKK7,^7,8^ which were expected to be useful for treatment of inflammatory and immunoregulatory disorders, but since the filing of two patent applications, no further progress has been reported.

As recently reported,^9^ we achieved a significant breakthrough with the discovery of HRX215 (INN: darizmetinib). Darizmetinib demonstrated exceptional efficacy in various pharmacological models after intravenous and oral administration. After successful completion of the IND-enabling safety program and a first-in-human Phase 1 trial, a Phase 2a safety and efficacy study in patients undergoing resection of colorectal liver metastases was launched (ClinicalTrials.gov ID NCT06638502).

Darizmetinib is a result of an efficient kinase selectivity tuning approach using BRaf^V600E^-inhibitor vemurafenib (VMF), which also inhibits MKK4 with moderate potency. In this paper, we present the medicinal chemistry approach to dissect the structure–activity relationship (SAR) of VMF with a focus on BRaf and MKK4, the exploration and optimization of multiple chemical scaffolds, and the results of iterative rounds of design and optimization which finally led to the selection of darizmetinib as a first-in-class MKK4-inhibitor that entered clinical development. This project also underlines that drug discovery programs on a new and innovative target can be successfully realized in a biotech-academic collaboration setting.

## Results and Discussion

### Strategy and Hit Identification

When our program was initiated, only a few MKK4 inhibitors were reported, most of which showed limited selectivity. Interest in MKK4 inhibition increased after Bayer disclosed in a 2004 patent a series of 4-phenyl-pyrimido­[4,5*-b*]­indoles as dual MKK4/7 inhibitors with IC_50_ values ≤ 1 μM.^7^ In 2013, Krishna et al. described trihydroxyisoflavone derivatives as MKK4 inhibitors,^10^ followed by Kim et al. with protoberberine derivatives in 2014.^11^ More recently, in 2017, Deibler et al. identified AST-487, PLX4720, Pazopanib, and related compounds as MKK4 inhibitors exhibiting low-micromolar potency.^12,13^

Based on our literature and patent search, the clinically approved BRaf^V600E^ inhibitor VMF, an analog of PLX4720, was identified as a promising scaffold for developing MKK4 inhibitors, owing to its MKK4 off-target activity and druglike properties.^14^ Our biochemical analyses confirmed that VMF binds to MKK4 with high affinity (K_D_ = 13.5 nM) and efficiently blocks JNK1 phosphorylation (IC_50_ = 0.8 μM). To assess whether VMF also interferes with MKK4 activation, we established a reconstituted MEKK2–MKK4–JNK1 kinase cascade assay that recapitulates the physiological activation process. Using this assay, we found that VMF inhibits MEKK2-dependent phosphorylation and activation of MKK4 (IC_50_ = 7 μM), indicating that VMF targets both the active and inactive state of MKK4.^9^ Disclosed crystal structures of nonphosphorylated MKK4 included the AMP-PNP-bound structures 3ALN^15^ and 3ALO,^15^ as well as the APO structure 3VUT.^16^ Unfortunately, despite sufficient resolution, all structures are burdened by a low anisotropy, which precluded an accurate pose prediction, and therefore, a drug discovery supportive VMF:MKK4 model could not be established.

Since a docking approach was not possible, we hypothesized that VMF may bind to MKK4 in a manner similar to its interactions with BRaf. VMF is a Type 1 ^1^/_2_ inhibitor of BRaf and, based on the biochemical inhibition of both MKK4 activation and MKK4 activity, a similar orientation was hypothesized. In the crystal structure 4RZV,^17^ VMF occupies the ATP-binding cleft of the BRaf (R509H) kinase domain monomer so that the kinase is trapped in the inactive DFG-in conformation (Figure 2A). Superimposing the crystal structures 3ALO (MKK4) and 4RZV (BRaf) suggests that the 7-azaindole serves as a hinge binding motif with interactions to Met^181^ and Glu^179^. Furthermore, the interaction between the sulfonamide nitrogen and the Asp^594^ backbone amide in BRaf corresponds to the sulfonamide *N-H*:Asp^247^ bridge in MKK4. According to this model, the sulfonyl moiety interacts with Phe^248^, whereas in BRaf the sulfonyl oxygens interact with Lys^483^ and Gly^596^. Consequently, the chlorophenyl moiety is oriented toward the solvent, and the difluorophenyl sulfonamide is positioned into a hydrophobic pocket (Figure 2B).

**Figure 2:**
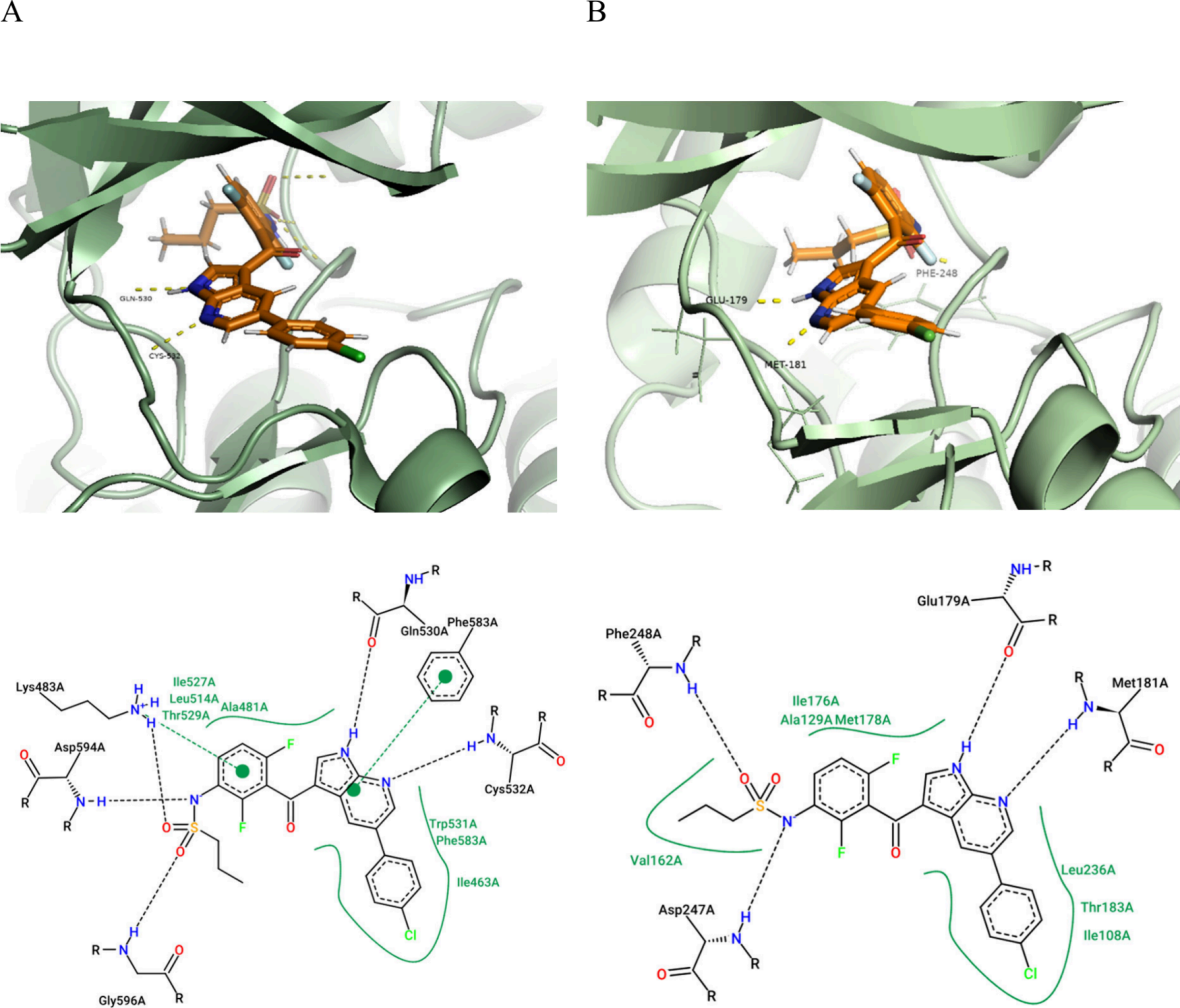
A. Binding mode of VMF in BRaf, elucidated by X-ray crystallography (4RZV); B. Hypothesized binding mode of VMF in MKK4. 2D plots were created with PoseView.^19^

VMF is also a potent inhibitor of the human leucine zipper- and sterile alpha-motif containing kinase ZAK (MAP3K20), but, as elucidated with the crystal structure 5HES,^18^ VMF occupies the ATP-binding pocket in the cleft between the N- and C-lobes of the kinase domain and stabilizes the DFG-in conformation in a type 1 inhibitor manner.

Our objective was to modify VMF to improve the inhibition potency against MKK4 with no significant activity toward RAF-kinases, and to improve and maintain the selectivity against JNK1 and MKK7, respectively. Based on the results of the genetic suppression of both kinases, an inhibition of the activity of either JNK1 or MKK7 would reduce or abolish the pro-regenerative effect of MKK4-inhibition (see Figure 1).

Since a docking approach was not possible, we elaborated relevant SARs experimentally based on the assumption of the above-described model. The chemical structure of VMF offers multiple opportunities for structural modifications. As shown in Figure 3, the molecule was dissected into moieties “a” to “e”. Initially, the hinge-binding 7-azaindole “a” and the carbonyl bridge “c”, the joint region between the 7-azaindole and the arylsulfonamide, were modified.

**Figure 3:**
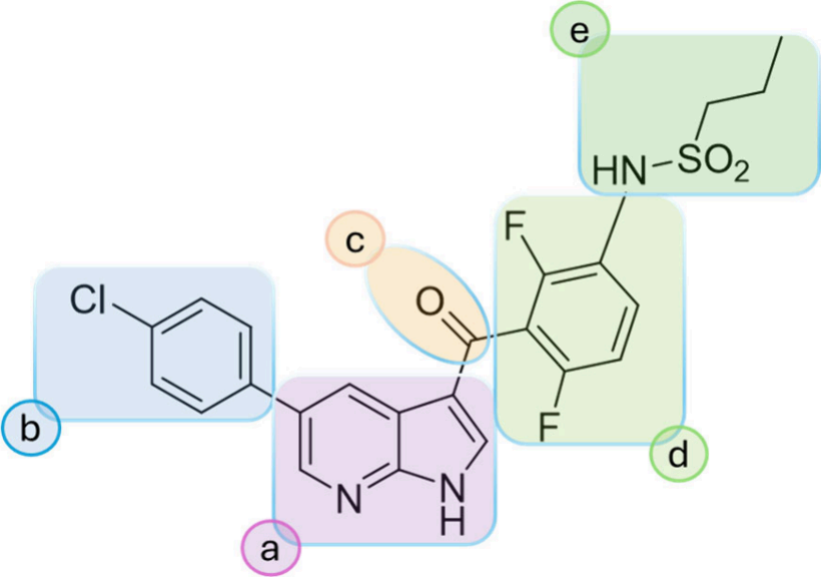
Chemical structure of VMF highlighting modification sites a–e.

In patent applications, 1*H*-pyrazolo­[3,4*-b*]­pyridines and 5*H*-pyrrolo­[2,3*-b*]­pyrazine have been disclosed as BRaf inhibitors but not further developed. To investigate, whether these scaffolds are advantageous regarding shifting the kinase selectivity toward MKK4-inhibiton, the corresponding VMF-analogs were synthesized. In addition, the 7-azaindole ring was further substituted to learn whether steric extensions at the hinge-binding motif are tolerated and if so, whether the selectivity can be improved. The results of these modifications are summarized in Table 1 and demonstrate that only unsubstituted 7-azaindole **1** and 1*H*-pyrazolo­[3,4*-b*]­pyridine **2** showed a promising MKK4 binding affinity.

**Table 1:**
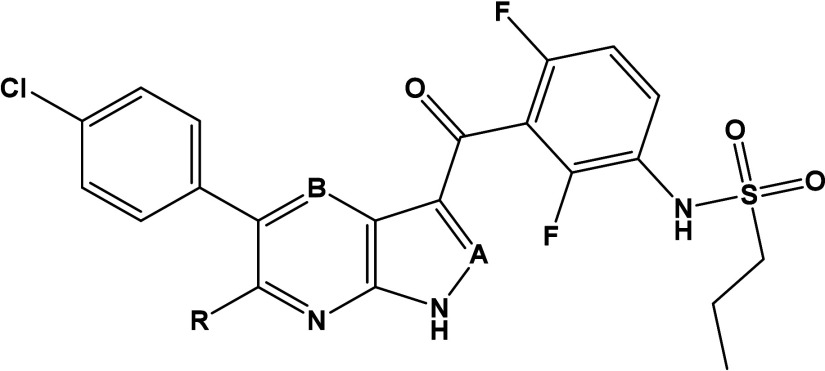
Affinity of a-Modified Vemurafenib Analogues to MKK4 and BRaf, Determined at a Concentration of 100 nM and Quantified by Calculation of PoC^a^

				**Binding (PoC) [%]**	
**Compound**	**A**	**B**	**R**	**MKK4**	**BRaf**
**1** (VMF)	-CH=	-CH=	-H	14	16
**2**	-N=	-CH=	-H	12	25
**3**	-CH=	-N=	-H	90	91
**4**	-CH=	-C(Cl)=	-H	65	95
**5**	-CH=	-C(CN)=	-H	100	100
**6**	-CH=	-CH=	-OCH_3_	90	100

a Percent of control; 100 = no binding, 0 = quantitative occupation; ligands were modified in position “a”.

We also explored the potential of 9*H*-pyrimido­[2,3*-b*]­indoles, disclosed by Bayer as dual inhibitors of MKK4 and MKK7.^7,8^ Two reference compounds of the patent application, compounds **7** and **8**, were prepared but the MKK4 potency was modest only (Table 2). To further modify the potent 1*H*-pyrrolo­[2,3*-b*]­pyridine (7-azaindole) core of VMF, based on the assumption of higher flexibility of MKK4 in the hydrophobic region than BRaf, the pyrido­[2,3*-b*]­indole derivatives **9a**–**c** were synthesized, which represented an attractive starting point for development of selective MKK4 inhibitors (Table 2). Provided that the pyridine and the indole-*NH* match the 7-azaindole and pyrazolopyridine binding to the hinge motif, the carbonylphenylpropane-1-sulfonamide group would be shifted significantly toward the hydrophobic region in two dimensions. This shift with the present interaction partners of the carbonylphenylpropane-1-sulfonamide moiety such as donor/acceptor- and π-interactions as well as steric demand, seemed to be more tolerated in MKK4 than in BRaf, as assumed.

**Table 2:**
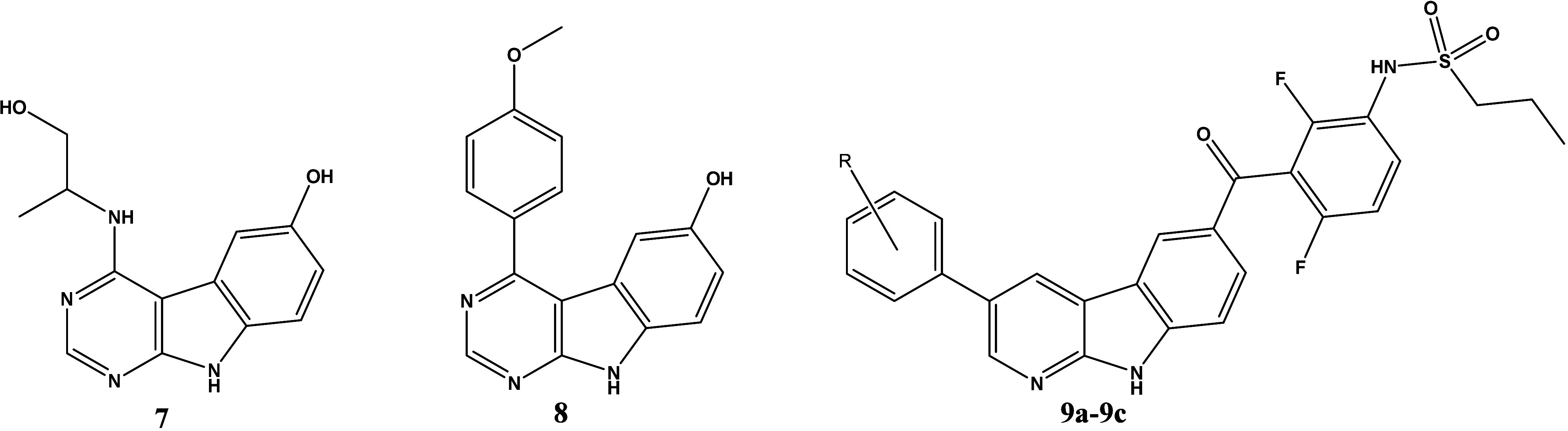
Affinity of 9*H*-Pyrimido­[2,3-*b*]­indoles, Dual MKK4/7 Inhibitors and of Novel Pyrido­[2,3-*b*]­indoles to MKK4, BRaf and ZAK, Determined at a Concentration of 100 nM and Quantified by Calculation of PoC and MKK4 Inhibition Potency, Determined with a Functional Biochemical Assay

		**Binding (PoC) @100 nM [%]**			
**Compound**	**R**	**MKK4**	**BRaf**	**ZAK**	**Inhibition (MKK4) IC**_**50**_ **[μM]**
**7**	N/A	66	91	81	15
**8**	N/A	17	82	84	1.2
**9a**	(o)-F	2.3	87	100	0.13
**9b**	(p)-Cl	11	100	100	N/D
**9c**	(p)-OH	1.2	89	100	0.07

In crystal structures of the 7-azaindole PLX4720 in BRaf and of VMF in BRaf^V600E^ and ZAK, the functional role and interaction of the carbonyl linker with the proteins were not fully elucidated. With ZAK, an interaction with the side chain of Asp^151^, which is bridged via a water molecule, has been postulated. The pyrido­[2,3*-b*]­indoles **9a**–**9c**, where the position of the carbonyl group is shifted relative to the hinge binding motif, showed no binding affinity to ZAK (Table 2) and essentially no binding to BRaf. Therefore, it was questioned whether a substitution of the carbonyl group in 7-azaindoles would lead to a similar improvement of selectivity against BRaf and ZAK. Several modifications were made, first to determine if the carbonyl moiety is necessary, second, if so, whether isosteres could lead to an improvement, and, finally, to assess if extension of the joint region is tolerated. Unfortunately, all carbonyl isosteric approaches as well as extended versions thereof resulted in a complete loss of MKK4 binding affinity (Table 3). The necessity of the carbonyl was clearly indicated with compound **1**. Interestingly, amide **10i** was active toward BRaf with a much better selectivity profile than VMF within the tested set of kinases.

**Table 3:**
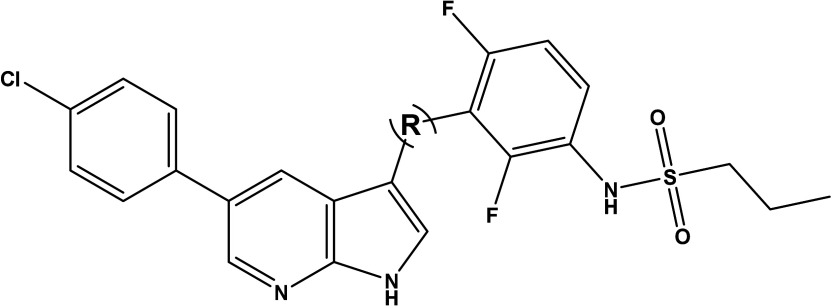
Affinity of c-modified Vemurafenib Analogues to MKK4 and BRaf, Determined at a Concentration of 100 nM and Quantified by Calculation of PoC^a^

		**Binding (PoC) [%]**				**Binding (PoC) [%]**	
**Entry**	**R**	**BRaf**	**MKK4**	**Entry**	**R**	**BRaf**	**MKK4**
**1 (VMF)**	CO	16	14	**10e**	CH=CH	96	64
**10a**	CH_2_	100	94	**10f**	(CO)-(CO)	100	100
**10b**	S	100	100	**10g**	(COH)-(COH)	100	100
**10c**	SO	100	100	**10h**	CONH	100	99
**10d**	SO_2_	100	100	**10i**	NHCO	20	97

a Ligands were modified in position “c”.

To explore whether a modification of the presumed solvent-exposed region b (Figure 3) would lead to an increase in potency and selectivity, a series of derivatives with mono- and disubstituted phenyl rings were synthesized, and the affinity to MKK4, BRaf, JNK1 and ZAK was determined. Results have been described in detail by Pfaffenrot et al.,^20^ and Kloevekorn et al.^21^

Compound **11a** (Table 4) showed a high binding affinity to MKK4 with improved selectivity against BRaf and ZAK. Furthermore, **11a** was highly selective against JNK1 and therefore considered a first tool compound to investigate the efficacy in *in vivo* models. Further characterization of this compound as well as the 1*H*-pyrazolo­[3,4*-b*]­pyridine analog **11b** included the investigation of the oral bioavailability in mice, a kinome profile (functional activity assay with 320 wild-type kinases) and a pharmacological safety screen on 44 safety relevant targets^22^). At test concentrations of 0.3 μM and 3 μM, kinome selectivity scores of 0.003 and 0.113 (**11a**) as well as 0.003 and 0.166 (**11b**) were determined. At the concentration of 3 μM, the activity of 35 kinases was inhibited by >50%. Interestingly, at the test concentration of 3 μM, 77% and 80% inhibition of JNK1 activity was observed for **11a** and **11b**, respectively, and both compounds inhibited MKK7 by some 50%. The inhibition of JNK1 in the functional assay contrasts the results of the binding assay, possibly related to different conformations of the kinases being used in binding and functional assays. The potency against JNK1 and MKK7 activity disqualified both candidates as tool compounds for pharmacological *in vivo* testing. To include an appropriate set of functional kinase assays into the primary screening strategy, compounds **11a** and **11b** as well as the pyrido­[2,3*-b*]­indole **9c** were subjected to IC_50_-value determination on 20 targets, which were selected based on the results of the kinome screening. The results are summarized in Table 5 and demonstrate again that the selectivity against JNK1 and also against BRaf and other protein kinases is not sufficient to allow the use of either **11a** or **11b** in experimental *in vivo* liver regeneration models. Therefore, further modifications of 7-azaindoles and 1*H*-pyrazolo­[3,4*-b*]­pyridines were made, including nonsubstituted and substituted heterocycles as b-modifications according to Figure 3.

**Table 4:**
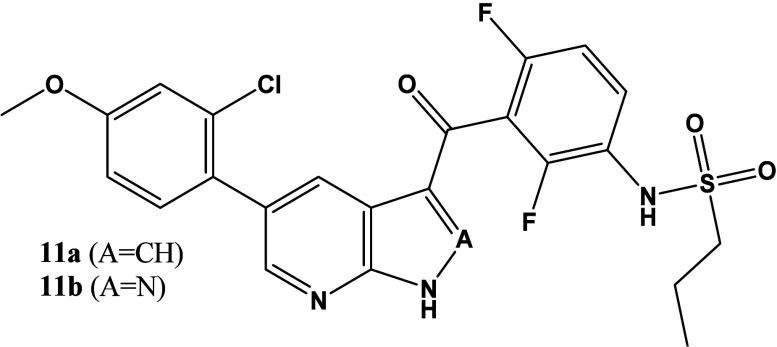
Binding Affinity of **11a** to MKK4, BRaf, and JNK1 and Selectivity, Calculated Based on K_d_-Values

**11a**	**MKK4**	**BRaf**	**JNK1**
K_d_ [nM]	4.8	190	1400
Selectivity K_d_(target)/K_d_ (MKK4)	-	40	292

**Table 5:** Inhibition of Selected Protein Kinases by 11a, 11b, and 9c^a^

	**IC**_**50**_ **[μM]**				**IC**_**50**_ **[μM]**		
**Kinase**	**11a**	**11b**	**9c**	**Kinase**	**11a**	**11b**	**9c**
**BLK**	1,04	1,50	0,19	**MKK4**	0,32	0,34	0,07
**B-RAF wt**	0,15	0,19	5,27	**MKK7**	>100	70,9	89,8
**BRK**	0,21	0,34	16,4	**p38-beta**	0,68	1,45	12,8
**FYN wt**	0,80	1,69	0,32	**RAF1 Y340D/Y341D**	0,11	0,18	0,83
**GSK3-alpha**	2,26	3,54	0,81	**RET wt**	2,13	1,26	0,32
**HRI**	2,01	1,36	7,77	**RIPK2**	0,20	0,52	0,24
**JNK1**	1,24	1,19	1,75	**SRMS**	0,03	0,06	15,0
**LCK**	0,42	1,45	0,50	**VEGF-R2**	1,48	0,91	0,98
**LYN**	0,88	2,21	0,57	**YES**	2,30	1,47	0,38
**MAP4K5**	0,05	0,05	<0.003	**ZAK**	0,24	1,25	3,35

a IC_50_-values were calculated based on determination of protein kinase activity in the presence of test compounds at 10 concentrations between 0.003 μM and 100 μM.

Primary screening focused on MKK4 and BRaf binding affinity, as a sufficient MKK4 binding affinity and selectivity against BRaf was considered as a prerequisite for further characterization. Most promising substituents were also transferred to the pyrido­[2,3*-b*]­indole scaffold. Results and detailed information of these studies have been recently published by Juchum et al.,^23^ Pfaffenrot et al.^20^ and Kloevekorn et al.^21^ A graphical representation of most potent compounds on MKK4 (POC range 0–30%) in comparison to BRaf is shown in . An MKK4 binding affinity, which was 20- to almost 40-fold stronger than that to BRaf, was achieved with both bicyclic scaffolds, the 7-azaindoles and 1*H*-pyrazolo­[3,4*-b*]­pyridines, when the phenyl ring was substituted in the para-position with a polar group such as carboxylic acid or sulfonamide. The introduction of *N*-heterocycles also resulted in a selectivity improvement. While the improvement of selectivity with the bicyclic hinge binding motifs was modest only, all but two pyrido­[2,3*-b*]­indole derivatives exhibited exceptional binding preference to MKK4 with PoC-values below 2% at the test concentration of 100 nM, while BRaf-binding was very low (PoC @ 100 nM > 70%).

Next, to address the hydrophobic region as a binding site with selectivity-gain potential, region “e” (Figure 3) was modified. First “e”-modifications were just derivatives of VMF and, based on these results, promising structural elements from previous modifications were combined with most potent “e”-modifications.

The arylsulphonamide moiety in VMF was found to interact optimally with the backbone amide of Asp^594^ (DFG-motif) and the propyl tail group was selected because it fits an interior pocket specific to the mutant BRaf protein.^24^ Further to our findings that substitution of the sulfonamide by an amide eliminates the affinity toward MKK4,^21^ we focused on alkyl chain modifications to potentially escape from the critical BRaf affinity. At first, a linear extension as well as a reduction of the *n*-alkyl residue was carried out, confirming that the n-propylsulfonamide apparently fits best into the BRaf binding pocket. The highest MKK4 binding affinity was determined with the n-butyl (PoC = 4.5), the α-ketopropyl (PoC = 3.5) and the benzyl (PoC = 5.5) substitutions (Table 6). The benzyl derivative **12k** was considered most favorable based on the essential elimination of BRaf affinity and the improved MKK4 affinity. Detailed study results are described by Kloevekorn et al.^21^

**Table 6:**
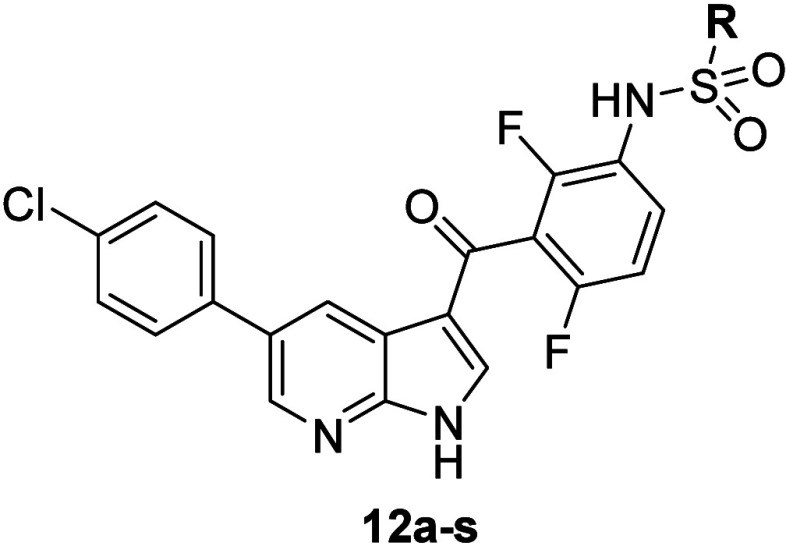
Affinity of e-modified Vemurafenib Analogues to MKK4 and BRaf, Determined at a Concentration of 100 nM and Quantified by Calculation of PoC^a^

		**Binding (PoC) [%]**				**Binding (PoC) [%]**	
**Compound**	**R**	**MKK4**	**BRaf**	**Compound**	**R**	**MKK4**	**BRaf**
**12a**	-CH_3_	16	93	**12j**	-phenyl	3.3	22
**12b**	-CH_2_-CH_3_	20	90	**12k**	-benzyl	5.9	100
**1**	nC_3_H_7_	14	16	**12l**	-ethylphenyl	24	99
**12c**	cyclo-prop	45	87	**12m**	-(CH_2_)_2_-CF_3_	2.8	32
**12d**	nC_4_H_9_	4.5	86	**12n**	-CH_2_-C(O)-CH_3_	3.5	61
**12e**	-CH_2_-iprop	23	56	**12o**	-CH_2_-C(OH)-CH_3_	26	35
**12f**	nC_5_H_11_	29	96	**12p**	-CH_2_-(o)F-phenyl	6	100
**12g**	-(CH_2_)_2_-iprop	31	96	**12q**	-CH_2_-(m)F-phenyl	13	100
**12h**	nC_6_H_13_	45	96	**12r**	-CH_2_-(p)F-phenyl	15	95
**12i**	c-hexyl	21	95				

a Ligands were modified in position “e”.

Consequently, a set of benzylated 7-azaindoles and 1*H*-pyrazolo­[3,4*-b*]­pyridines with the most potent “b”-modifications were synthesized, leading to candidates with tool compound properties. The characteristics of candidates that were short-listed are summarized in Table 7 (structure and binding affinity data) and Table 8 (inhibition potency and oral bioavailability).

**Table 7:**
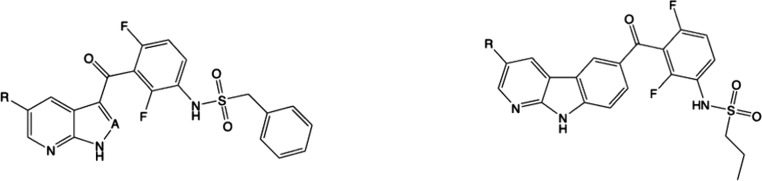

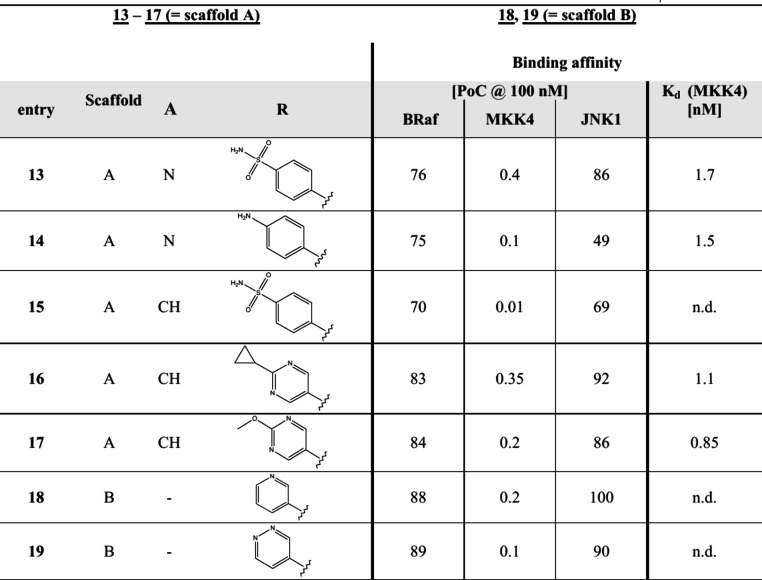
PoC Data at 100 nM and K_d_ Values of Potential Tool Candidates

**Table 8:** Inhibition Potency, Selectivity against BRaf and JNK1 and Oral Bioavailability of MKK4 Inhibitors

	**Inhibition Potency [μM] and Selectivity (IC**_**50**_ **(kinase)/IC**_**50**_ **(MKK4) in Functional Enzyme Assays**					**PK (Mouse; 30 mg/kg p.o.)**		
**Test Compound**	**MKK4**	**BRaf**	**Selectivity**	**JNK1**	**Selectivity**	**C**_**max**_ **[μM]**	AUC [μM*h]	**T**_**1/2**_ **[h]**
**13**	0.15	0.22	1.5	0.22	1.5	0.594	2.88	4.0
**14**	0.15	0.68	4.5	0.16	1.1	N/D	N/D	N/D
**15**	0.17	0.23	1.4	0.21	1.2	N/D	N/D	N/D
**16**	0.21	0.86	4.1	2.06	9.8	63.1	205	2.2
**17**	0.29	2.44	8.4	1.84	6.3	N/D	N/D	N/D
**18**	0.06	48.5	808	1.60	26.7	43.1	108	3.3
**19**	0.11	3.14	28.5	6.23	56.6	1.31	5.02	2.8

All compounds show a strong binding affinity to MKK4 (PoC at 100 nM = 0.01–0.4; K_d_: 0.85–1.7 nM) and an acceptable selectivity when compared to binding data determined for BRaf and JNK1, both at a concentration of 100 nM. In functional enzyme assays, the IC_50_-values for MKK4 varied between 0.056 and 0.286 μM, which was considered acceptable, provided that sufficient blood concentrations are achieved in mice after oral administration. With consideration of the pharmacokinetic data and inhibition profiles of BRaf and JNK1, compounds **16** and **18** were selected for further characterization.

In drug discovery, cell-based assays are generally considered mandatory to characterize the potency of drug development candidates as quantification of intracellular potency covers the characteristics of cellular permeability and intracellular unspecific binding phenomena. To investigate the potency of stress signaling pathway inhibitors, after stress-induction, the extent of phosphorylation of a downstream readout, which is specific for the activity of the target, is quantified. This strategy cannot be applied for MKK4, as the immediate downstream substrates, JNK and p38, are also activated by MKK7 (JNK) and MKK3/MKK6 (p38). As initially demonstrated with VMF, compounds **16** and **18** not only inhibit the activity but also the MEKK2-mediated activation of MKK4 with IC_50_-values of 1.77 μM and 1.06 μM, respectively. Therefore, after stimulation of the stress signaling pathway, the abundance of phospho-MKK4 should be reduced in the presence of test compounds. We employed the HAP1-cell as test system, and after preincubation with **16** and **18** for 60 min, cells were stimulated with anisomycin followed by Western blot detection of p-MKK4 and p-p38 MAPK in cell lysates. The results, shown in Figure 4, demonstrate a concentration-dependent abundance of p-MKK4 in the presence of **16**, while compound **18**, despite its higher potency in biochemical assays, had no impact on MKK4 phosphorylation. Apparently, the membrane permeability of the tricyclic motif of compound **18** is too low to reach the site of action in intact cells. VMF, which was included in this test series, inhibited MKK4 phosphorylation as well but was less potent than **16**.

**Figure 4:**
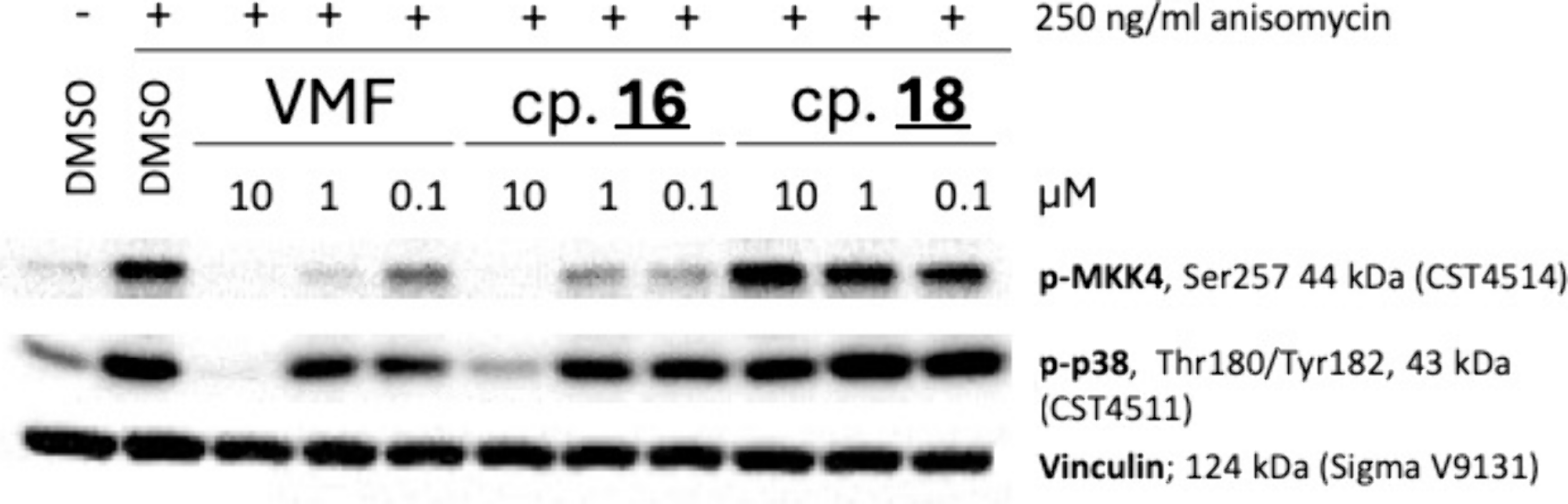
Western-blot detection of phospho-MKK4 and phospho-p38 MAPK in lysates of HAP1 cells after anisomycin-stimulation of the stress signaling pathway in the absence or presence of MKK4-inhibitors. Test compounds were preincubated for 30 min prior to addition of ansiomycin.

### *In Vivo* Proof of Concept with Compound **16**

To investigate the effect of **16** on the kinetics of liver regeneration, the 2/3 hepatectomy model in mice, developed by Mitchell and Willenbring,^25^ was employed. With consideration of the only 10-fold selectivity against JNK1 and, after oral administration, the elimination half-life of 2.2 h, 10 mg/kg was given twice daily, with the first dose administered 1h prior to hepatectomy. After 48 h, mice were sacrificed, and the kinetics of liver regeneration was determined by quantification of Ki67 in liver tissue sections. In samples from animals treated with compound **16**, 21.15 ± 9.30% (N = 6) of Ki67 positive cells were determined compared to 9.14 ± 6.87% (N = 6) in livers from vehicle treated animals (). To verify whether **16** also supports the stabilization of hepatocytes after induction of liver injury, employing the same dose regimen, animals received a single dose of CCl_4_ 1h after the first drug administration, and for investigation of the extent of liver damage, apoptotic hepatocytes were visualized and quantified by TUNEL staining. In livers from animals dosed with compound **16**, the number of TUNEL-positive cells was significantly lower compared to vehicle-treated animals (1.12 ± 0.82% vs 3.52 ± 2.17, N = 6, p = 0.0101). The hepato-protective characteristics is supported by lower AST and ALT enzyme activity, determined in plasma at study termination (AST: 2,643 ± 1,469 vs 1,396 ± 999 U/L (p = 0.0618); ALT: 2,991 ± 1,100 U/L vs 2,164 ± 1,203 (p = 0.1593) ().

In mice, ethanol feeding for 10 days followed by a single binge ethanol administration induces liver injury, inflammation and fatty liver, thus mimicking acute-on-chronic alcoholic liver injury in patients (, NIAAA model^26^). To investigate the efficacy of **16**, dosing commenced on day 5 of chronic ethanol feeding, and the last dose was administered prior to the ethanol binge. Animals were sacrificed 9 h later for determination of fat deposition and liver enzyme activity in plasma. Treatment with **16** resulted in a significantly less pronounced liver steatosis (6.24 ± 1.27% vs 8.32 ± 2.25% white area, p = 0.0308, ) while the effect on liver enzymes was moderate only (). Thus, in experimental models in mice, the administration of **16** resulted in an accelerated liver regeneration after hepatectomy, protected hepatocytes after acute liver injury, and in a lower steatosis, which was induced by subchronic ethanol feeding. However, to translate the preclinical proof-of-concept into the clinic, a candidate with higher MKK4 inhibition potency and an improved selectivity profile needed to be discovered.

### Discovery of Compound **42** (Darizmetinib)

Compound **16** was designed based on the optimization of the molecular moieties a, b, c and e (Figure 3), while group d (the difluorophenyl moiety) remained unchanged. Further to the observation that **20a**, the nonfluorintated equivalent of VMF showed no BRaf binding affinity, the monofluorinated derivatives **20b**-**20e** were synthesized (Table 9). A substantial gain in MKK4-affinity was not achieved, but encouraged by the preferential MKK4-binding of **20b** and **20c** over BRaf, difluorinated **20f** was prepared which showed a highly promising MKK4 affinity and selectivity against BRaf.

**Table 9:**
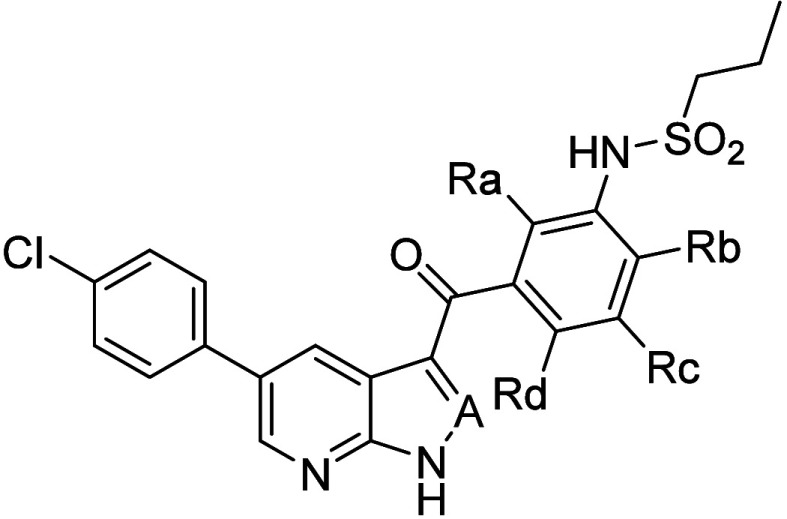
Affinity of 7-Azaindoles (A = CH) and 1H-Pyrazolo­[3,4-*b*]­pyridines (A = N) to MKK4 and BRaf, Determined at a Concentration of 100 nM and Quantified by Calculation of PoC^a^

						**Binding Affinity [PoC @ 100 nM]**	
**Compound**	**A**	**Ra**	**Rb**	**Rc**	**Rd**	**MKK4**	**BRaf**
**VMF**	CH	-F	-H	-H	-F	14	16
**20a**	CH	-H	-H	-H	-H	53	100
**20b**	CH	-F	-H	-H	-H	16	31
**20c**	CH	-H	-F	-H	-H	32	94
**20d**	CH	-H	-H	-F	-H	100	91
**20e**	CH	-H	-H	-H	-F	45	82
**20f**	CH	-F	-F	-H	-H	6	87
**20g**	CH	-F	-H	-F	-H	17	4
**21**	N	-F	-F	-H	-H	N/D^a^	N/D^a^
**22**	CH	-F	-F	-F	-H	N/D^a^	N/D^a^
**23**	CH	-F	-H	-F	-F	N/D^a^	N/D^a^
**24**	CH	-F	-F	-H	-F	N/D^a^	N/D^a^
**25**	N	-F	-F	-H	-F	2.8	N/D^a^

a Ligands were modified in position “d”.

b Functional biochemical assays only (see Table 10).

The results of functional biochemical assays with **20f** and the 1*H*-pyrazolo­[3,4*-b*]­pyridine analog **21** confirmed the advantage of the 2,6-difluorophenyl pattern (Table 10), which was even more pronounced for **21**. Introduction of a third fluorine (**22**–**25**) had no additional beneficial effect regarding the potency and selectivity. The comparison of data from 7-azaindoles (**20f**, **22**–**24**) with those of **21** and **25** suggest that the beneficial biochemical profile of the 2,6-difluorophenyl pattern is more pronounced in combination with the 1*H*-pyrazolo­[3,4*-b*]­pyridine hinge binding motif.

**Table 10:** Inhibition (IC_50_) of MKK4, BRaf and JNK1 by Compounds **20f** and **21**–**25** and Selectivity Factors Derived from IC_50_-Values

	**MKK4**	**BRaf**	**selectivity**	**JNK1**	**selectivity**
**Compound**	**IC**_**50**_ **[μM]**	**IC**_**50**_ **[μM]**	$\frac{{\mathrm{IC}}_{50}\left(\mathrm{BRaf}\right)}{{\mathrm{IC}}_{50}\left(\mathrm{MKK}4\right)}$	**IC**_**50**_ **[μM]**	$\frac{{\mathrm{IC}}_{50}\left(\mathrm{JNK}1\right)}{{\mathrm{IC}}_{50}\left(\mathrm{MKK}4\right)}$
**20f**	0.09	0.92	10.2	2.72	30.2
**21**	0.04	8.20	205	3.31	82.8
**22**	0.11	0.91	8.3	1.89	17.2
**23**	0.36	0.02	0.1	1.47	4.1
**24**	0.11	1.32	12.0	0.96	8.7
**25**	0.09	24.4	271	3.76	41.2

Based on these significant findings, the “b” and “e” modifications described above were combined with the optimized 2,6-difluorophenyl 7-azaindole and 1*H*-pyrazolo­[3,4*-b*]­pyridine structure. In addition, an MKK4 protein NMR structural biology project was launched to eventually elucidate the binding mode of the new ligands to MKK4.

For all synthesized compounds, the 2,6-difluorophenyl substitution resulted in a substantially higher MKK4 binding affinity () and inhibition potency () when compared to those of the corresponding 2,4-difluorophenyl counterparts. Furthermore, as presented in Table 11, BRaf activity is inhibited, if at all, at substantially higher concentrations only, while the selectivity improvement against JNK1 is influenced by the sulfonamide substituent as well. In particular, the benzylsulfonamide group leads to a moderate JNK1-inhibition potency (Table 11, compounds **30** and **40**). The most favorable balance between MKK4 potency and selectivity against BRaf and JNK1 was observed with n-propyl sulfonamide derivatives. Compared to the kinase inhibition profile of tool compound **16**, lead optimization led to MKK4 inhibitors with some 10-fold potency, while the very weak inhibition of BRaf and JNK1 was no longer considered critical. Further kinase inhibition testing included MKK7, the MAP2 kinase upstream of JNK1, which may not be inhibited, but for all compounds given in Table 11, if at all, only very weak inhibition was observed with IC_50_-values above 100 μM.

**Table 11:**
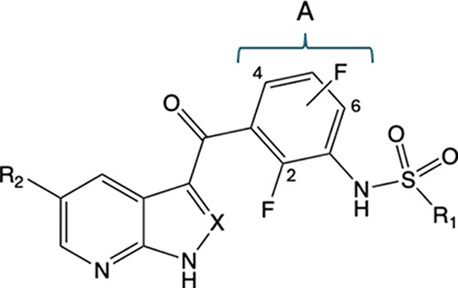

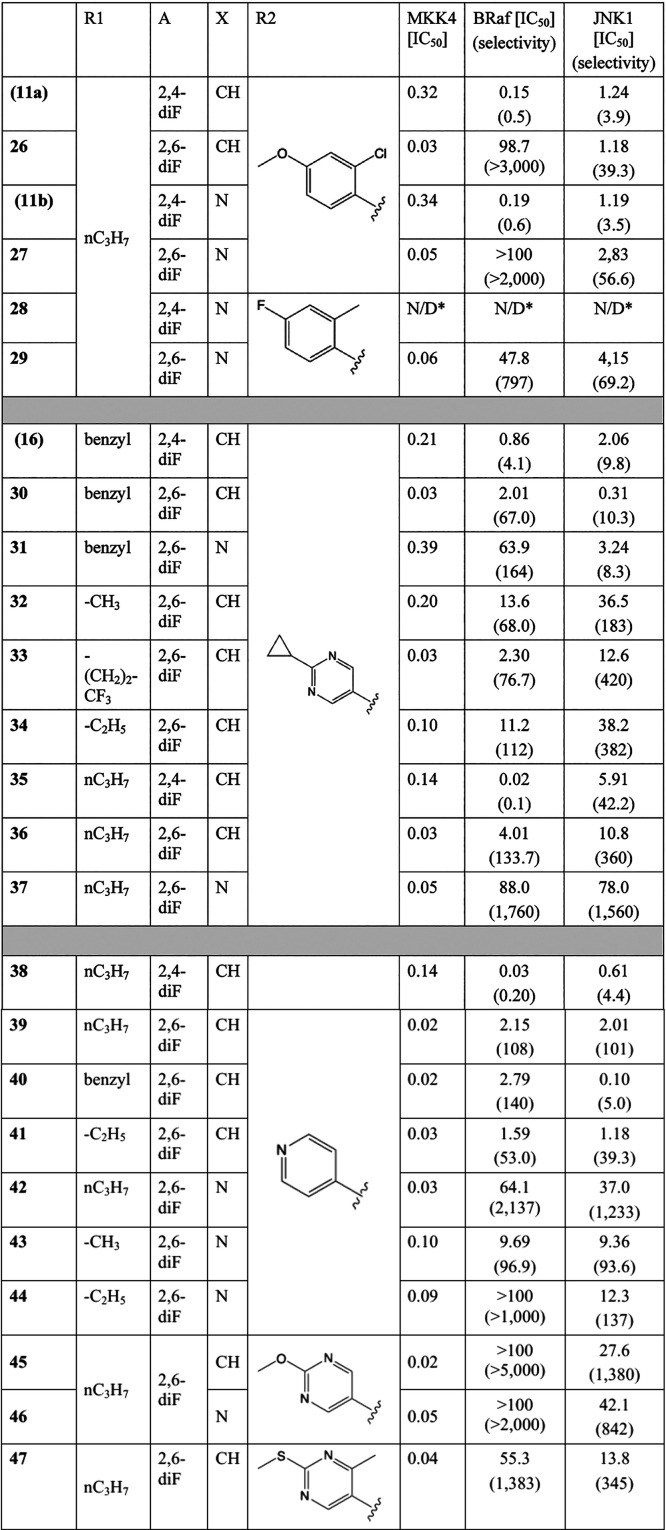
Effect of 2,6- vs. 2,4-Difluorphenyl Substitution on the Potency and Selectivity of MKK4 Inhibitors^a^

a Selectivity factors derived from IC_50_-values as defined in Table 10.

Based on the MKK4/BRaf/JNK1-inhibition profiles, 33 7-azaindole and 23 1*H*-pyrazolo­[3,4*-b*]­pyridine derivatives were selected to assess the oral bioavailability in mice, and based on the results, six potential candidates were shortlisted followed by investigation of the metabolic stability in liver microsomes from mice, rats, dogs and humans (Table 12). Except for **47**, which showed a very low oral bioavailability and high intrinsic clearance, the pharmacokinetic and metabolic stability characteristics of the remaining five compounds were acceptable.

**Table 12:** ADME Profiling of Shortlisted Candidates^a^

	**Mouse PK (Dose: 30 mg/kg p.o.)**			***In Vitro* Metabolism** **CL_int_ [mL/min/kg][Bibr ref1]**			
**Test compound**	***C*_max_** **[μM]**	**AUC_0‑t_** [μM*h]	***T*_1/2_** **[h]**	MLM*	RLM*	DLM*	HLM*
**36**	36.5	237	4.0	36.0	10.0	7,0	4,0
**37**	25.4	156	4.4	143	365	4.5	5.1
**42**	67.4	122	3.9	228	658	33.8	10.8
**45**	13.3	162	4.8	21,3	12.2	–1.5	0.3
**46**	31.0	229	6.4	143	1,503	4.8	4.6
**47**	0.7	2.9	2.2	600	197	159	51.0

a Oral bioavailability was investigated by single administration of test compounds at a dose of 30 mg/kg followed by serial blood micro sampling and quantification of parent compounds by LC-MS/MS. Metabolic stability was investigated by incubation of test compounds in pooled liver microsomes from mice (MLM), rats (RLM), dogs (DLM) and humans (HLM). Incubations were performed in the presence of the co-factor NADPH.

A first safety assessment was performed by investigating potential inhibition of the hERG-ion channel and inhibition of cytochrome P450 enzymes. The results of the hERG channel assay, performed by whole-cell patch-clamp technique are given in Table 13. For three compounds, at the highest possible test concentrations of 10 and 30 μM, the ion current was inhibited by less than 50%. For **36** (IC_50_ = 6.72 μM) and for **42** (IC_50_ = 24.4 μM), a sufficient safety margin was concluded, and therefore, all compounds were subjected to the CYP-inhibition panel.

**Table 13:** Effect of Short-Listed Candidates on the hERG-Channel Current^a^

	hERG IC_50_ [μM]	$safety\;factor:\frac{{IC}_{50\left(hERG\right)}}{{IC}_{50\left(MKK4\right)}}$
**36**	6.72	236
**37**	>30	>700
**42**	24.4	840
**45**	>10	>450
**46**	8.71	223
**47**	>30	>600

a The safety factor was calculated based on IC_50_-values.

The inhibition of CYPs was investigated without and with preincubation of test compounds in the absence or presence of NADPH, so that potential mechanism-based inhibition would be detected (). A weak to moderate inhibition potency with IC_50_-values in the μM-range was observed for **36** and **47** (CYPs 2C8 and 2C9), as well as for **42** (CYPs 2B6, 2C8, 2C9, 2C19, and 2D6), which, however, would not exclude any of the candidates for further development. Thus, based on biochemical MKK4 inhibition potency, protein kinase selectivity, oral bioavailability, hERG and CYP-inhibition, a ranking of the short-listed candidates was not possible. Therefore, all candidates were subjected to the experimental 2/3-hepatectomy model in mice, which was employed with tool compound **16** to validate the preclinical concept. As a minor modification, compounds were administered orally only 12h and 1h prior to hepatectomy and animals were sacrificed 42h after surgery. The results of the quantification of Ki67-positive cells in liver tissue are summarized in Figure 5. When compared to vehicle-treated animals, all five compounds induced an accelerated proliferation of hepatocytes, the extent of this effect, however, was different. Compound **37** induced a strong and dose-dependent pro-proliferative effect with a mean of 35.0 ± 4.3% Ki67-positive cells compared to 4.8 ± 1.8% cells counted in liver tissue from vehicle-treated animals. A nice dose-dependent effect was also recorded for compound **36**, although at a lower level compared with **37**. With compound **42**, a strong and significant effect was observed in animals at the low dose level of 0.4 mg/kg when compared with the vehicle group (15.4 ± 2.0% vs 4.9 ± 0.6% Ki67-positive cells, *p* < 0.0001).

**Figure 5:**
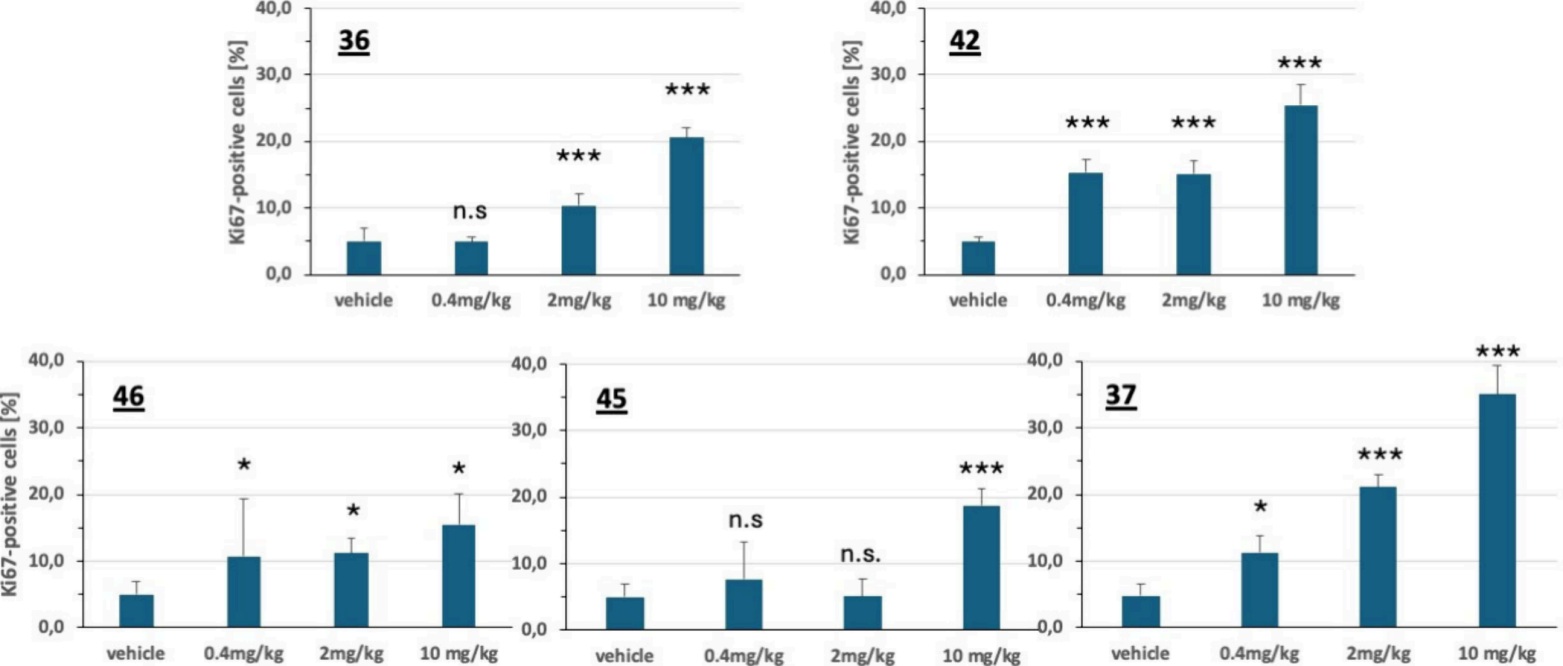
Induction of liver regeneration by treatment of C57BL/6N mice with MKK4-inhibitors prior to 2/3-hepatectomy. Quantification of Ki67-expressing cells performed 42h after hepatectomy.

To investigate whether compounds **36**, **42** and **37** also stabilize hepatocytes in acute liver injury, the test articles were prophylactically administered to mice at 12h and 1h prior to and 24h after i.p. injection of carbon tetrachloride (CCl_4_). 48 h after liver injury induction, the extent of apoptosis was investigated either histologically (TUNEL-Assay, compound **37**) or by quantification of cleaved caspase 3 in liver homogenates (compounds **36** and **42**). In addition, for drug monitoring, blood samples were collected 0.5h after the second dose and 24 h after the third dose. When compared to control animals, which received vehicle only, the administration of MKK4-inhibitors resulted in a reduced number of apoptotic cells (Figure 6). The most pronounced and statistically significant hepatocyte stabilization was observed with compounds **37** and **42**, while for compound **36**, the reduction of cleaved caspase 3 was not significant. For all compounds, blood concentrations, determined at two different time points, increased with increasing doses in a dose-proportional manner ().

**Figure 6:**
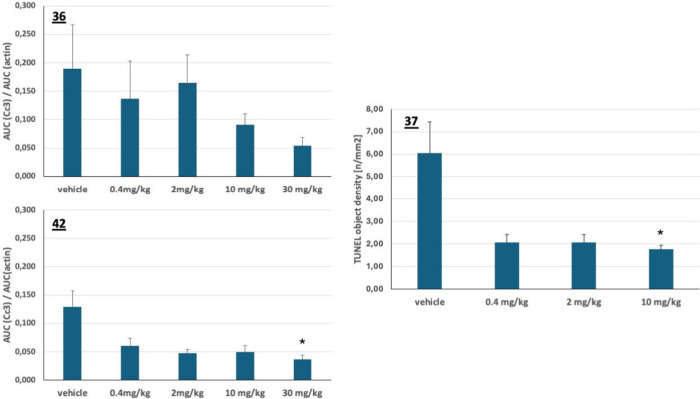
Prevention of Hepatocyte Apoptosis by Treatment of C57BL/6N mice with MKK4-inhibitors. Test compounds were administered prior to and after induction of acute liver injury (*= *p* < 0.01).

Based on the pharmacological profiles, both, compound **42** (HRX215, darizmetinib) and compound **37** were considered as potential clinical candidates. In a 14-day pharmacokinetic study in Wistar rats, both compounds were administered at doses of 10, 30, and 100 mg/kg/day. To investigate the pharmacokinetics, serial blood samples were collected by microsampling on days 1 and day 14. During the treatment period, no adverse clinical signs were recorded, and clinical chemistry remained unchanged for all treatment groups. As shown in , the rate and extent of exposure of animals to compound **42** increased with ascending doses in a slightly subproportional manner. For compound **37** (), an increased exposure was observed between 10 and 30 mg/kg but at the dose of 100 mg/kg, the exposure, quantified by the pharmacokinetic parameters *C*_max_ and AUC, was lower when compared to the 30 mg/kg group. Therefore, it is anticipated that the exposure of Wistar rats to compound **37** will be limited when administered by the oral route even at very high doses. However, nonclinical safety studies are designed to determine the safety profile and identification of target organ toxicity. During the 14-day treatment with both compounds, no adverse events were observed and no signs of toxicity in mice with either liver-specific^3^ or ubiquitous,^9^ shRNA-mediated, knockdown of MKK4, were seen. Therefore, it was hypothesized that a high systemic exposure of the MKK4-inhibitor will be required to detect a target organ toxicity, which led to the nomination of compound **42** (darizmetinib) as a clinical development candidate and not compound **37**.

### Binding Mode by NMR

To elaborate the binding mode in more detail, protein NMR analysis was performed using compound **48** as described by Zwirner et al.^9^ (Figure 7). The binding mode derived from protein NMR analysis confirmed our initial hypothesis regarding the similar orientation of VMF based inhibitors in MKK4, with 7-azaindole as hinge binder to Met^181^ NH and Glu^179^ C=O. The proposed interaction of the sulfonamide with Phe^248^, based on our structural alignment of 3ALO with 4RZV was also confirmed. Protein-NMR analysis gave additional insights into the SARs such as the interaction of the sulfonamide with the Gly^249^ backbone NH, which serves as an H-bond donor to the sulfoxide oxygen. Furthermore, the sulfonamide nitrogen NH also interacts with Asp^247^ and Lys^131^ through a water bridged interaction. Asp^247^ is further engaged by a water mediated interaction involving the carbonyl oxygen of the joint region. Additionally, the 2-F atom interacts with Cys^246^ SH, accompanied by a Cys^246^ sulfonamide interaction. No distinct interaction was observed for the pyridazine ring, suggesting that the pyridazine ring is relatively exposed and dynamic.

**Figure 7:**
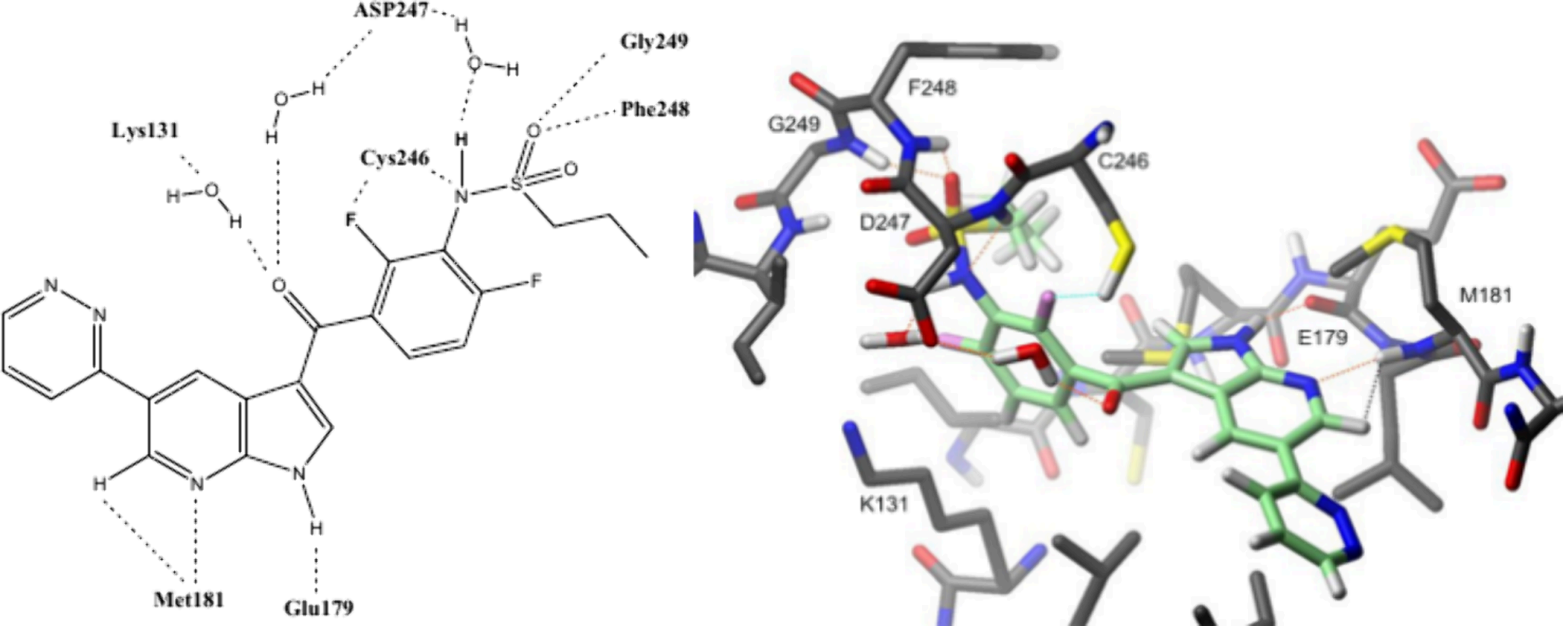
MKK4 binding mode of cp. **48**, from protein-NMR analysis.

### Chemistry

The synthesis of 7-azaindole derivatives (Scheme 1) commenced with difluoronitrobenzoic acid, which was converted into its corresponding benzoic acid chloride with oxalyl chloride. The resulting acid chloride **A0** was then subjected to a Friedel–Crafts acylation using 5-bromo-1*H*-pyrrolo­[2,3-*b*]­pyridine as the nucleophile, resulting in the formation of intermediate **A2**. Intermediate **A2** was subsequently reduced using tin­(II) chloride to yield the corresponding aniline, **A3**. Attempts to perform direct Suzuki coupling reactions with unprotected **A4** proved inefficient on a larger scale, and also on small scale, the reactions only succeeded under harsh conditions. To address this limitation, **A3** was protected with dichlorobenzoyl chloride to yield **A4**. The sulfonamidation of **A4** with propanesulfonyl chloride produced a mixture of mono- and disulfonamides. This crude mixture was treated with sodium hydroxide, which selectively hydrolyzed the undesired disulfonamide, affording the monosulfonamide **A5** in high purity. At this stage, two synthetic routes were followed depending on the availability of boronic acids. If aryl-heteroarylboronic acids were preferred, direct Suzuki coupling of **A5** was performed to obtain the protected product **A7**. If aryl/heteroaryl bromides were preferred, a Miyaura borylation reaction was carried out to synthesize the corresponding boronic ester **A6**. **A6** was then used in a subsequent Suzuki coupling with the corresponding aryl/heteroaryl bromides. Both pathways led to the desired protected target molecule **A7**. Finally, deprotection of the dichlorobenzoyl group was achieved under basic conditions, yielding the corresponding 7-azaindole derivative in its final form. This multistep process allowed for a scalable synthesis of the target compounds with flexibility depending on reagent availability.

**Scheme 1:**
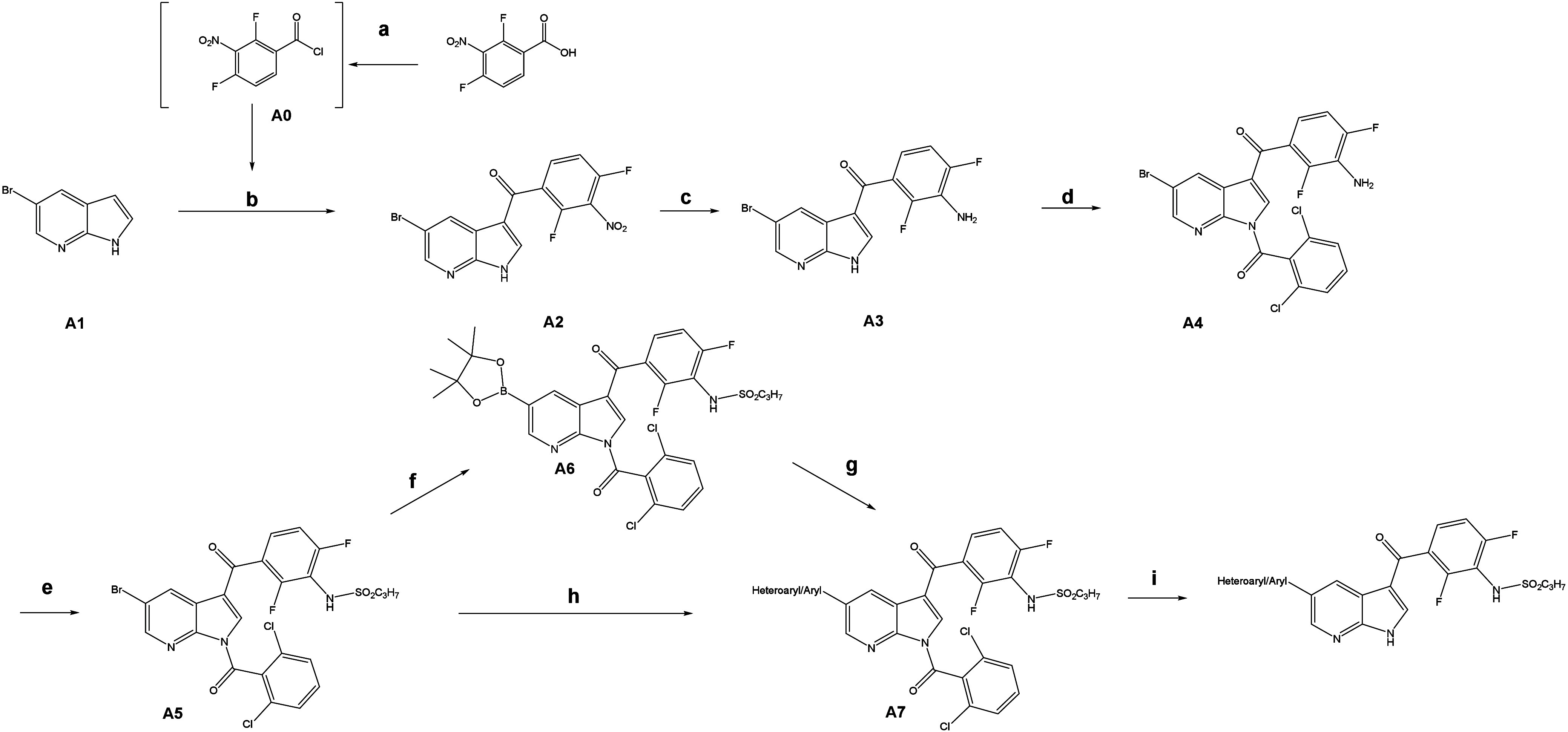
(a) Nitromethane, Oxalylchloride, DMF, RT; (b) AlCl_3_, Nitromethane, RT–55 °C, MeOH, Water (75%); (c) SnCl_2_, EtOAc/THF, 60 °C, NaHCO_3_, MeCN (80%); (d) THF, 2,6-Dichlorobenzoyl Chloride, TEA 0° C–RT (80%); (e) 1. THF, TEA, 1-Propanesulfonyl Chloride −10 °C 2. NaOH, RT, HCl, *n*-Pentane (70–80%) (f) B_2_Pin_2_, Dioxane, Pd­(dppf)­Cl_2_, KOAc, 60–80 °C MeCN (70%); (g) Pd­(dppf)­Cl_2_, Aryl Bromide Dioxane/Water KF or K_2_CO_3_, 60–90 °C (50–70%); (h) Boronic Acid or Pinacol Boronate, Pd­(dppf)­Cl_2_, K_2_CO_3_, Dioxane/Water (4/1) 60–90 °C (i) MeOH, K_2_CO_3_, RT (50–70%)

The synthesis of 1H-pyrazolo­[3,4-*b*]­pyridine derivatives (Scheme 2) needed a different strategy than that for the 7-azaindoles, as the reactivity of the core structure differs significantly. In the 7-azaindole synthesis in Scheme 1, the Friedel–Crafts reaction served as a central step early in the process. However, this reaction was ineffective for pyrazolopyridine **B1** in Scheme 2 due to the unfavorable electronic effects of the 1H-pyrazolo­[3,4-*b*]­pyridine, which substantially decreases the required reactivity. Consequently, an alternative synthetic strategy was necessary to address this limitation. This alternative strategy (Scheme 2) involved the use of the Weinreb amide **B5** as a key intermediate, in combination with a metalated aniline. The metalated aniline was generated through the reaction of its corresponding bromide with isopropylmagnesium chloride, producing the reactive Grignard reagent. The synthesis of Weinreb amide **B5** was performed via iodination of the pyrazolopyrimidine, followed by a carbonylation step to form the corresponding carboxylic acid **B3**. **B3** was then converted to the imidazolid derivative. The imidazolid was further transformed into Weinreb amide **B5**. Generation of **B6** needed deprotonation of the pyrazolopyridine **B5** and *in situ* protection of the aniline prior to reaction of both intermediates. The ongoing synthesis was similar to that of the 7-azaindoles, just the protecting group of the pyrazolopyridine was switched to tetrahydropyranyl. Also here, dependent on the availability of boronic acids and halogenides, either a direct Suzuki coupling was performed or a Miyaura borylation, followed by Suzuki coupling to yield the protected target compound **B9**, which was then deprotected with hydrochloric acid. Both approaches provided flexibility in adapting the synthesis to the availability of key starting materials while ensuring efficient assembly of the final structure.

**Scheme 2:**
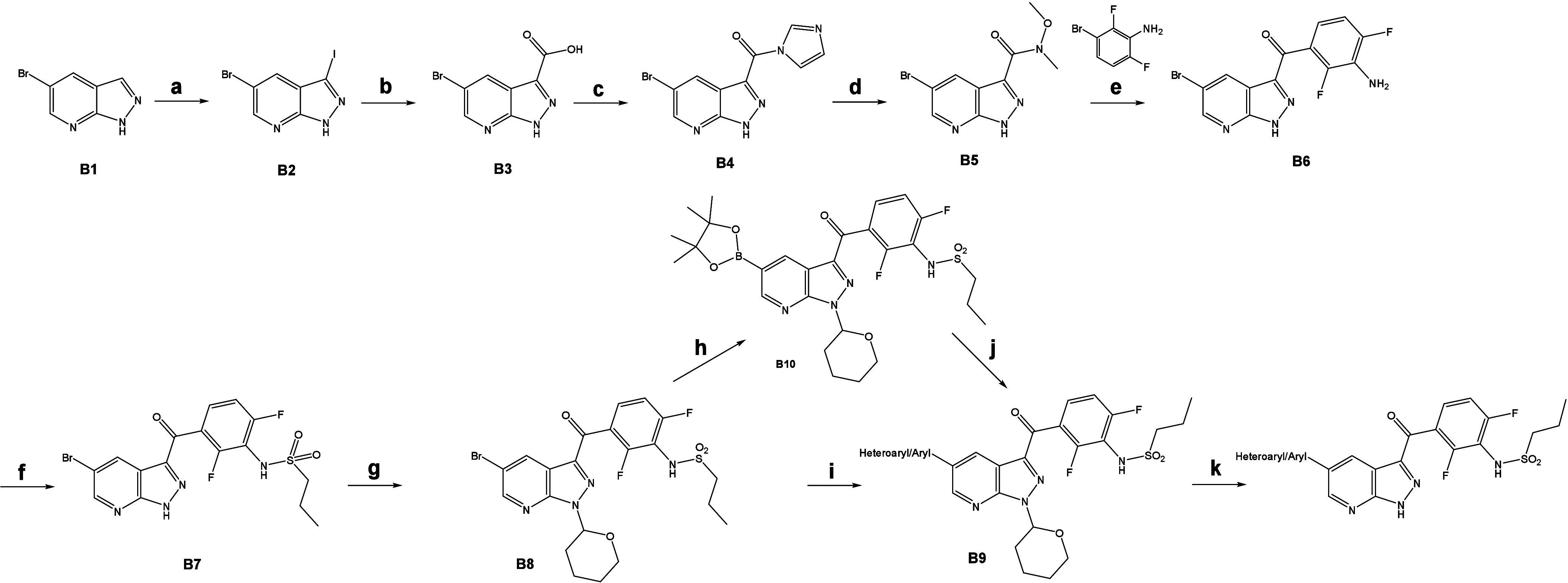
Synthesis of 1H-Pyrazolo­[3,4-*b*]­pyridine Derivatives (a) I_2_, KOH, DMF, RT (92%); (b) 1.) CO­(g), Xantphos, Pd­(OAc)_2_, Et_3_N, MeOH, DMF, 60 °C, 2.) NaOH, H_2_O, 95 °C–RT (quant.); (c,d) CDI, N,O-Dimethylhydroxylamine Hydrochloride, 60 °C (85%); (e) 1.) 2,4-Difluoroaniline, n-BuLi, TMS-Cl, NaH −78 °C – −15 °C, 2.) conc. HCl, RT (70%); (f) Propanesulfonyl Chloride, Pyridine, 65 °C (65%); (g) DHP, p-TsOH, DCM, reflux (75%); (h) B_2_Pin_2_, AcOK, Pd­(dppf)­Cl_2_, 1,4-Dioxane, 85 °C (91%); (i) Corresponding Boronic Acid or Pinacol Boronate, PdCl_2_(dppf), Cs_2_CO_3_, 1,4-Dioxane, H_2_O,Rf; (j) 1.) Corresponding aryl Bromide, Pd­(PPh_3_)_4_ or XPhos Pd G3, K_2_CO_3_, 1,4-dioxane, H2O, 55 °C, 2.) HCl, i-PrOH, 70 °C

## Conclusions

Wuestefeld et al.^3^ identified the protein kinase MKK4 as a key regulator of liver regeneration and verified the therapeutic potential in disease models. In experimental, acute, and chronic liver disease models, genetic downregulation of MKK4-expression resulted in a rewiring of stress signaling leading to prevention of hepatocyte death and accelerated liver regeneration. At that time, a small molecule inhibitor of MKK4 was not available to verify whether this highly attractive therapeutic concept can be exploited for development of a new medicine. VMF, a BRaf-inhibitor approved for treatment of BRaf^V600E^-driven melanoma, is also a moderate inhibitor of MKK4 and we successfully applied a kinase-tuning strategy to create darizmetinib, a MKK4-inhibitor, which is currently in clinical development. As described, based on a hypothetical binding orientation of VMF in MKK4, the hinge binding motif and the substituents were sequentially modified. Through such iterative explorations, valuable insights into structure–activity relationships (SAR) were obtained. These efforts culminated in the development of **16**, our first tool compound, which demonstrated suitable selectivity and promising *in vivo* efficacy in a 2/3 hepatectomy model.

Further optimization, particularly fluorination patterns, led to a series of some 50 (33 7-azaindole and 23 1H-pyrazolo­[3,4*-b*]­pyridine) 2,6-difluorophenyl derivatives with significantly improved selectivity and potency. From these, based on pharmacokinetic properties, six compounds were shortlisted as potential clinical candidates.

The selection of compound **42** (darizmetinib, HRX215) as first-in-class MKK4 inhibitor was based on the potency in experimental *in vivo* models and dose-linear pharmacokinetics in mice and rats. After completion of the nonclinical, IND-enabling safety program, a first-in-human Phase 1 study has been completed, which demonstrated an excellent safety of darizmetinib and a dose-linear pharmacokinetics up to the highest dose explored. Details are disclosed in Zwirner et al.^9^ The safety and efficacy of darizmetinib are currently being investigated in a clinical Phase 1*b*/2a study in colorectal cancer patients with liver metastases undergoing a minor and major hepatectomy (ClinicalTrials.gov ID NCT06638502).

## Experimental Section

### Chemistry

Reagents and solvents were purchased from commercial sources and used without purification. The purity of all compounds was determined by HPLC to be greater than 95%. ^1^H NMR and ^13^C NMR spectra were recorded on a Bruker Avance 400 spectrometer, operating at 400 MHz for ^1^H NMR and 101 MHz for ^13^C NMR or a Bruker Avance 200 MHz spectrometer. Chemical shifts (δ) and multiplicity data are given according to the ACS NMR guidelines. Chemical shifts were expressed in parts per million relative to tetramethylsilane (TMS) as an internal standard, and J values were reported in Hertz (Hz). Flash chromatography was carried out on Interchim PuriFlash XS420 flash chromatography system and Grace Davison Davisil LC60A 20e45 mm silica. Purity of the compounds was determined by HPLC analysis on Agilent 1100 Series Liquid Chromatograph using a Phenomenex Luna C8 150 × 4.6 mm, 5 mm column with gradient elution (MeOH/0.01 M KH_2_PO_4_ buffer, pH 2.3, flow rate 1.5 mL/min) and detection at λ = 230 and 254 nm. All final compounds were determined with >95% purity if not stated otherwise.

Detailed procedures can be found in the .

Details to Binding Affinity assays, Functional kinase inhibition assays, Experimental *in vivo* models and Pharmacokinetic studies are described in detail in the .

### Synthesis of **42** (darizmetinib, HRX215)

#### 5-Bromo-3-iodo-1*H*-pyrazolo­[3,4*-b*]­pyridine (**B2**)

To a stirred mixture of 5-bromo-1H-pyrazolo­[3,4*-b*]­pyridine (6.81 g, 34.4 mmol) and KOH (6.75 g, 120.4 mmol) in DMF (45 mL) was added iodine (9.60 g, 37.8 mmol) in one portion at room temperature (RT). After a short induction period, the exothermic reaction began. After 1 h, about 1 g of iodine was added and the mixture stirred at 45 °C for 1 h. The mixture was poured into a dilute solution of Na_2_SO_3_ and then acidified with 2 N HCl. The solids were collected by suction filtration, washed with water, and dried in an oven at 110 °C. Yield: 10.92 g (92%). ^1^H NMR (200 MHz, DMSO-*d*_6_) δ: 14.29 (s, 1H), 8.62 (s, 1H), 8.17 (s, 1H); ^13^C NMR (50 MHz, DMSO-*d*_6_) δ: 150.53, 150.17, 131.86, 120.58, 112.43, 91.95; [M-H]^−^ = 322.0/324.0.

#### 5-Bromo-1*H*-pyrazolo­[3,4*-b*]­pyridine-3-carboxylic acid (**B3**)

5-Bromo-3-iodo-1*H-*pyrazolo­[3,4*-b*]­pyridine **B2** (10.44 g, 32.2 mmol) was dissolved in DMF, MeOH and triethylamine (TEA, 75 mL each). The vessel was evacuated and backfilled with argon (4x). XantPhos (1.12 g, 1.93 mmol) and Pd­(OAc)_2_ (270 mg, 0.97 mmol) were added, and carbon monoxide (generated from formic acid and sulfuric acid) was bubbled through the solution while heating to 60 °C. The mixture was stirred under an atmosphere of carbon monoxide (balloon) for 8 h. Every 1.5 h carbon monoxide was bubbled through the solution for 5 min. The mixture was concentrated under reduced pressure, and the residue was triturated with 2 N HCl. The solids were heated at 95 °C in about 100 mL 1N NaOH overnight. After cooling to RT, the mixture was acidified with conc. HCl, and the precipitate collected by suction filtration and washed with water. The solids were dried in an oven at 110 °C. After complete drying, the product was sonicated in 100 mL of toluene for 5 min and stirred for 30 min. The product was filtered, washed with an additional 20 mL of toluene and dried at 110 °C. Yield: 7.92 g (quant.). ^1^H NMR (200 MHz, DMSO-*d*_6_) δ: 8.64 (d, J = 7.9 Hz, 2H), 5.69 (bs, 1H); ^13^C NMR (50 MHz, DMSO-*d*_6_) δ: 163.27, 150.97, 149.67, 136.69, 132.65, 115.73, 113.6; [M-H]^−^= 239.9/241.9.

#### 5-Bromo-*N-*methoxy-*N-*methyl-1*H-*pyrazolo­[3,4*-b*]­pyridine-3-carboxamide (**B5**)

5-bromo-1*H-*pyrazolo­[3,4*-b*]­pyridine-3-carboxylic acid **B3** (7.91 g, 32.7 mmol) and 1,1′-carbonyldiimidazole (5.83 g, 35.9 mmol) were stirred in 200 mL of DMF at 60 °C for 45 min. *N*,*O*-dimethylhydroxylamine hydrochloride (3.51 g, 35.9 mmol) was added to the resulting suspension, and the mixture was stirred for 4 h at 65 °C. Most of the solvent was removed under vacuum and half sat. NaHCO_3_-solution was added to the residue. The solids were collected by suction filtration, washed with water, and dried at 110 °C. Yield: 7.94 g (85%). ^1^H NMR (200 MHz, DMSO-*d*_6_) δ: 14.46 (s, 1H), 8.62 (d, J = 20.4 Hz, 2H), 3.76 (s, 3H), 3.44 (s, 3H), [M-H]^−^= 283.0/285.0.

#### (3-Amino-2,6-difluorophenyl)­(5-bromo-1*H-*pyrazolo­[3,4*-b*]­pyridin-3-yl)­methanone (**B6**)

To a solution of 2,4-difluoroaniline (7.99 g, 61.9 mmol, 2.2 equiv) in THF (55 mL), 2N *n*-BuLi in *n*-hexane (24.8 mL, 61.9 mmol, 2.2 equiv) was added dropwise at −78 °C, followed by the dropwise addition of trimethylsilyl chloride (7.85 mL, 61.9 mmol) in THF (10 mL). The mixture was warmed to −50 °C and stirred for 10 min. After cooling to −78 °C, 2N *n*-BuLi in *n*-hexane (24.8 mL, 61.9 mmol, 2.2 equiv) and trimethylsilyl chloride (7.85 mL, 61.9 mmol) in THF (10 mL) were added consecutively dropwise, and the reaction was stirred for 30 min at RT. The mixture was cooled to −78 °C, 2N *n*-BuLi in *n*-hexane (24.8 mL, 61.9 mmol, 2.2 equiv) was added dropwise, and the mixture was stirred for 30 min. This is considered reaction solution A. In a separate flask, 60% NaH in mineral oil (1.24 g, 30.9 mmol, 1.05 equiv) was added in portions to a suspension of 5-Bromo-*N*-methoxy-*N*-methyl-1H-pyrazolo­[3,4-*b*]­pyridine-3-carboxamide **B5** in THF (55 mL) at 0 °C, and the mixture was stirred for 30 min at RT. This is considered reaction solution B. Reaction solution B was added to reaction solution A, warmed to −15 °C and stirred for 15 min. Conc. HCl (12 mL) was carefully added, and the mixture stirred for 5 min at RT. Saturated NaHCO_3_-solution was slowly added until phase separation occurred. The phases were separated, the aqueous phase extracted with THF, and the extracts were dried and filtered. Isopropanol (i-PrOH) (150 mL) was added to the extract, and the extract was concentrated to a volume of about 100 mL. The product was collected by suction filtration, washed with i-PrOH and dried. Yield:7.14 g (70%). ^1^H NMR (200 MHz, DMSO-*d*_6_) δ: 14.91 (s, 1H), 8.77 (dd, J = 5.4, 2.1 Hz, 2H), 7.18–6.59 (m, 2H), 5.25 (s, 2H); ^13^C NMR (50 MHz, DMSO-*d*_6_) δ: 183.95, 151.04, 150.79, 150.27 (dd, J = 161.0, 6.8 Hz), 145.50 (dd, J = 167.3, 6.8 Hz), 141.34, 133.35 (dd, J = 12.8, 2.6 Hz), 132.28, 117.45 (dd, J = 8.4, 6.5 Hz), 116.24 (dd, J = 22.7, 19.1 Hz), 115.55, 114.81, 111.26 (dd, J = 21.7, 3.5 Hz); [M–H]^−^ = 351.1/353.1.

#### *N-*(3-(5-Bromo-1*H-*pyrazolo­[3,4*-b*]­pyridine-3-carbonyl)-2,4-difluorophenyl)­propane-1-sulfonamide (**B7**)

(3-amino-2,6-difluorophenyl)­(5-bromo-1*H-*pyrazolo­[3,4*-b*]­pyridin-3-yl)­methanone **B6** (2.00 g, 5.66 mmol) and 4-DMAP (35 mg, 0.28 mmol, 5%) were heated in 9 mL of pyridine to 65 °C and 1-propanesulfonyl chloride (1.21 g, 0.96 mL, 8.50 mmol) was added. After 2 h another 0.19 mL of 1-propanesulfonyl chloride were added. The warm solution was added to about 80 mL 2 N HCl, the solids collected and washed with water. The solids were taken up in ethyl acetate (EtOAc) and washed with 2 N HCl and brine, dried over sodium sulfate (Na_2_SO_4_) and evaporated. The product was purified by flash chromatography (SiO_2_, DCM/EtOAc 0% to 20%) and triturated with *n*-hexane. Yield: 1.68 g (65%). ^1^H NMR (200 MHz, DMSO-*d*_6_) δ: 9.86 (s, 1H), 8.79 (dd, J = 5.3, 2.0 Hz, 3H), 7.64 (td, J = 9.0, 6.0 Hz, 1H), 7.30 (t, J = 8.9 Hz, 1H), 3.17–2.96 (m, 3H), 1.87–1.62 (m, 2H), 0.96 (t, J = 7.4 Hz, 4H); ^13^C NMR (50 MHz, DMSO-*d*_6_) δ: 182.75, 157.39 (dd, J = 177.1, 7.4 Hz), 152.42 (dd, J = 180.3, 7.3 Hz), 151.56, 151.34, 141.39, 132.59, 130.78–130.05 (m), 122.20 (dd, J = 13.5, 3.6 Hz), 117.19 (dd, J = 23.0, 20.9 Hz), 116.14, 115.18, 112.59 (dd, J = 22.2, 3.8 Hz), 54.14, 17.22 12.97; [M–H]^−^ = 457.1/459.1.

#### *N-*[3-[5-Bromo-1-(oxan-2-yl)­pyrazolo­[3,4*-b*]­pyridine-3-carbonyl]-2,6-difluorophenyl]­propane-1-sulfonamide (**B8**)

To a suspensions of *N-*[3-(5-bromo-1*H-*pyrazolo­[3,4*-b*]­pyridine-3-carbonyl)-2,4-difluorophenyl]­propane-1-sulfonamide **B7** (0.33 g, 0.73 mmol) in dichloromethane (DCM, 3 mL) dihydropyran (0.13 mL, 1.45 mmol, 2 equiv) and p-toluenesulfonic acid monohydrate (27 mg, 0.145 mmol, 0.2 equiv) were added, and the mixture was heated to reflux temperature for 45 min. After cooling, the mixture was washed with sat. NaHCO_3_-solution, dried over Na_2_SO_4_, filtered, and the solvent was removed under reduced pressure. The residue was dissolved in a minimum amount of DCM and added dropwise to *n*-hexane with stirring. After 5 min the solids were collected by suction filtration and dried. Yield: 297 mg (75%), used without further purification. ^1^H NMR (200 MHz, CDCl_3_) δ: 8.83 (s, 1H), 8.65 (s, 1H), 7.70 (dd, J = 13.6, 8.1 Hz, 1H), 7.02 (d, J = 9.6 Hz, 2H), 6.14 (d, J = 9.2 Hz, 1H), 4.14–3.64 (m, 2H), 3.22–2.90 (m, 2H), 2.61–2.31 (m, 1H), 2.15–1.16 (m, 10H); ^13^C NMR (50 MHz, CDCl_3_) δ: 182.4, 151.0, 149.7, 140.7, 133.7, 127.33 (d, J = 8.6 Hz), 121.36 (dd, J = 13.1, 3.8 Hz), 116.8, 116.6, 112.44 (dd, J = 22.6, 3.7 Hz), 83.3, 77.2, 68.2, 54.1, 28.9, 24.8, 22.4, 17.3, 12.9; [M–1]^−^ = 540.7.

#### *N-*(3-(5-(2-Cyclopropylpyrimidin-5-yl)-1*H-*pyrazolo­[3,4*-b*]­pyridine-3-carbonyl)-2,6-difluorophenyl)­propane-1-sulfonamide (42/HRX215)

A vessel was charged with N-[3-[5-bromo-1-(oxan-2-yl)­pyrazolo­[3,4-*b*]­pyridine-3-carbonyl]-2,6-difluorophenyl]­propane-1-sulfonamide (**B8**) (39.8 g, 73.2 mmol), bis­(triphenylphosphine)­palladium­(II) dichloride (514 mg, 0.732 mmol), and 4-pyridinylboronic acid (9.90 g, 80.5 mmol). Degassed 1,4-dioxane (183 mL) and degassed 2 M potassium carbonate (110 mL, 220 mmol) were added and the vessel was evacuated and filled with argon. The reaction was stirred at 80 °C overnight. After cooling, the reaction mixture was diluted with EtOAc and aqueous NH_4_Cl solution. The layers were separated and the aqueous layer was extracted with EtOAc. The extracts were dried and the solvent was removed. Residual water was removed azeotropically with toluene and ethanol (EtOH). The oily residue was taken up in 150 mL EtOH, 2.5 N HCl in EtOH (117 mL, 293 mmol) was added and the mixture was heated to reflux temperature for 8 h, then cooled to RT and stirred overnight. The solids were collected by suction filtration, suspended in i-PrOH (250 mL) then filtrated again. The filtrated product was taken up in MeOH (200 mL) and THF (200 mL) and then slowly added to sodium hydrogen carbonate (12.3 g, 146 mmol) in water (250 mL). After stirring for 10 min, the pH was adjusted to pH = 7, the organic solvents were removed under reduced pressure. The product was collected by filtration, washed with water, and dried at elevated temperature to yield N-[2,6-difluoro-3-(5-pyridin-4-yl-1H-pyrazolo­[3,4-*b*]­pyridine-3-carbonyl)­phenyl]­propane-1-sulfonamide (**42**) (27.9 g, 60.4 mmol, 82% yield) as off white solid.

The compound was synthesized in analogy to step i. ^1^H NMR (400 MHz, DMSO-*d*_6_) δ: 14.89 (s, 1H), 9.66 (s, 1H), 9.12 (d, J = 2.1 Hz, 1H), 8.91 (d, J = 2.3 Hz, 1H), 8.72 (d, J = 5.9 Hz, 2H), 7.91–7.83 (m, 3H), 7.40 (t, J = 8.8 Hz, 1H), 3.18 (q, J = 7.3 Hz, 2H), 1.33 (t, J = 7.3 Hz, 3H). ^13^C NMR (101 MHz, DMSO-*d*_6_) δ: 185.1, 160.8 (dd, J = 255, 3 Hz), 157.1 (dd, J = 257, 4 Hz), 152.7, 150.2, 149.4, 144.7, 142.0, 130.5 (dd, J = 10, 4 Hz), 129.5, 128.9, 123.6 (dd, J = 13, 3 Hz), 121.9, 114.3 (t, J = 17 Hz), 114.0, 112.0 (dd, J = 22, 3 Hz), 54.9, 16.9, 12.6.

### Synthesis of Compound **37**

#### *N-*[2,6-Difluoro-3-[1-(oxan-2-yl)-5-(4,4,5,5-tetramethyl-1,3,2-dioxaborolan-2-yl)­pyrazolo­[3,4*-b*]­pyridine-3-carbonyl]­phenyl]­propane-1-sulfonamide (**B10**)

A vessel was charged with *N-*[3-[5-bromo-1-(oxan-2-yl)­pyrazolo­[3,4*-b*]­pyridine-3-carbonyl]-2,4-difluorophenyl]­propane-1-sulfonamide (271 mg, 0.499 mmol), bis­(pinacolato)­diboron (139 mg, 0.55 mmol, 1.1 equiv), and anhydrous potassium acetate (147 mg, 1.50 mmol, 3 equiv). Degassed, dry 1,4-dioxane (5 mL) was added, and the vessel was evacuated and refilled with argon (3x). Pd­(dppf)­Cl_2_ (9.12 mg, 0.0125 mmol) was added, and the mixture was stirred at 85 °C overnight. After cooling to RT, the mixture was diluted with EtOAc, filtered over Celite, and the solvent was removed. The residue was dissolved in DCM, petrol ether (60/90) was added, and DCM removed under reduced pressure. After cooling for 1 h at 4 °C, the solids were collected by suction filtration and dried. Yield: 267 mg (91%), which was used without further purification. ^1^H NMR (200 MHz, CDCl_3_) δ: 9.11 (s, 1H), 8.94 (s, 1H), 7.68 (d, J = 5.9 Hz, 1H), 7.12–6.83 (m, 2H), 6.22 (d, J = 9.0 Hz, 1H), 4.19–3.60 (m, 2H), 3.07 (s, 2H), 2.57–1.11 (m)

#### *N*-(3-(5-(2-Cyclopropylpyrimidin-5-yl)-1*H*-pyrazolo­[3,4-*b*]­pyridine-3-carbonyl)-2,6-difluorophenyl)­propane-1-sulfonamide (**37**)

A vessel was charged with N-[2,6-difluoro-3-[1-(oxan-2-yl)-5-(4,4,5,5-tetramethyl-1,3,2-dioxaborolan-2-yl)­pyrazolo­[3,4-*b*]­pyridine-3-carbonyl]­phenyl]­propane-1-sulfonamide (**B10**) (48.1 g, 81.5 mmol), 5-bromo-2-cyclopropylpyrimidine (17.8 g, 89.6 mmol), potassium fluoride (14.2 g, 244 mmol), and 1,4-dioxane/water (4/1, 400 mL) and heated to 65 °C. The vessel was evacuated and filled with argon. Bis­(triphenylphosphine)­palladium­(II) dichloride (286 mg, 0.407 mmol) was added and the mixture was stirred at 65 °C for 18h. After cooling to RT, the layers were separated and the aqueous layer was extracted with EtOAc. The combined organic layers were washed with brine, dried over sodium sulfate and evaporated. The residual sticky oil was dissolved in EtOH (500 mL) under gentle heating. *n*-Hexane (50 mL) was added and the mixture was stirred at RT for 45 min. The precipitated intermediate was collected by filtration and washed with i-PrOH (200 mL). The filtrated product was resuspended in EtOH (250 mL) stirred for 10 min, then collected by filtration and dried at 60 °C. The product was taken up in MeOH (200 mL) and 2.5 N HCl in EtOH (200 mL), then heated to a gentle reflux, forming a solution. The mixture was heated to reflux temperature for 18 h. After cooling to RT, i-PrOH (250 mL) was added to the formed suspension and stirring was continued for 30 min. The product was collected by suction filtration, washed with i-PrOH (100 mL) and dried at 60 °C to yield N-[3-[5-(2-cyclopropylpyrimidin-5-yl)-1H-pyrazolo­[3,4-*b*]­pyridine-3-carbonyl]-2,6-difluorophenyl]­propane-1-sulfonamide hydrochloride (31.4 g, 58.0 mmol, 71% yield). ^1^H NMR (400 MHz, DMSO-*d*_6_) δ: 14.92 (s, 1H), 9.69 (s, 1H), 9.13 (s, 2H), 9.05 (d, J = 2.2 Hz, 1H), 8.87 (d, J = 2.2 Hz, 1H), 7.86 (dd, J = 14.8, 7.6 Hz, 1H), 7.40 (t, J = 8.6 Hz, 2H), 7.40 (t, J = 8.6 Hz, 1H), 3.19–3.11 (m, 2H), 2.32 (ddd, J = 12.7, 8.0, 4.8 Hz, 1H), 1.86–1.75 (m, 2H), 1.18–1.06 (m, 4H), 0.99 (t, J = 7.4 Hz, 3H).

### Synthesis of Compound **46**

#### *N-*(2,6-Difluoro-3-(5-(2-methoxypyrimidin-5-yl)-1*H-*pyrazolo­[3,4*-b*]­pyridine-3-carbonyl)­phenyl)­propane-1-sulfonamide (46)

The compound was synthesized by analogy to compound **37** using 5-bromo-2-methoxypyrimidine. ^1^H NMR (400 MHz, DMSO-*d*_6_) δ: 14.87 (s, 1H), 9.67 (s, 1H), 9.09 (s, 2H), 9.04 (d, J = 1.7 Hz, 1H), 8.86 (d, J = 1.6 Hz, 1H), 7.86 (dd, J = 14.3, 7.6 Hz, 1H), 7.40 (t, J = 8.8 Hz, 1H), 4.00 (s, 3H), 3.19–3.09 (m, 2H), 1.81 (td, J = 14.8, 7.3 Hz, 2H), 0.99 (t, J = 7.3 Hz, 3H).

### Synthesis of Compound **36**

#### Synthesis of (5-bromo-1*H-*pyrrolo­[2,3*-b*]­pyridin-3-yl)­(2,4-difluoro-3-nitrophenyl)­methanone (**A2**)

In one flask, 2,4-difluoro-3-nitrobenzoic acid (12.4 g, 60.9 mmol) was suspended in nitromethane (50 mL), and oxalyl chloride (5.44 mL, 63.4 mmol) was added, followed by a catalytical amount of DMF (0.197 mL, 2.54 mmol) which was added dropwise. In a second flask nitromethane (204 mL) was added to 5-bromo-1*H-*pyrrolo­[2,3*-b*]­pyridine (10 g, 50.8 mmol) and aluminum chloride (33.8 g, 254 mmol), the mixture was stirred at least for 1h. After complete activation of the carboxylic acid, the solution of flask 1 (the acid chloride) was added to flask 2 (AlCl_3_/7-azaindole flask) over a period of 10 min at RT. The mixture was warmed to 55 °C and heated overnight. After completion of the reaction (monitored by TLC and HPLC), the mixture was cooled to 0 °C, and MeOH (102 mL) was added dropwise under stirring, the product started precipitating. Water (169 mL) was added, stirring was continued for 1 h at RT and then the precipitate was filtered off and dried in vacuo. The product was obtained as a beige solid (15.2 g; 75% yield; 95% purity). ^1^H NMR (400 MHz, DMSO-*d*_6_) δ: 13.12 (br s, 1H), 8.64 (d, J = 2.45 Hz, 1H), 8.52–8.48 (m, 1H), 8.38–8.34 (m, 1H), 8.07–7.99 (m, 1H), 7.64 (t, J = 9.29 Hz, 1H).

#### Synthesis of (3-amino-2,4-difluorophenyl)-(5-bromo-1*H-*pyrrolo­[2,3*-b*]­pyridin-3-yl)­methanone (**A3**)

(5-bromo-1*H-*pyrrolo­[2,3*-b*]­pyridin-3-yl)-(2,4-difluoro-3-nitrophenyl)­methanone **A2** (15.1 g, 37.5 mmol) was suspended in EtOAc (659 mL) and THF (659 mL). The mixture was heated to 60 °C, and tin­(II) chloride dihydrate (29.6 g, 131 mmol) was added portion wise. After 2 days of stirring, TLC and HPLC revealed completion of the reaction. The mixture was cooled to RT and a half saturated NaHCO_3_ solution was added. The formed precipitate (Tin salts) was filtered off over a pad of sand and Celite. The filtrate was washed with brine and dried over sodium sulfate, successively. The solvent was evaporated under reduced pressure. The obtained solid was suspended in MeCN (50 mL), after stirring for 30 min the product was filtrated off, dried in vacuo to yield (3-amino-2,4-difluorophenyl)-(5-bromo-1*H-*pyrrolo­[2,3*-b*]­pyridin-3-yl)­methanone (11.9 g, 31.8 mmol, 85% yield) as light orange solid. ^1^H NMR (400 MHz, DMSO-*d*_6_) δ 12.92 (br s, 1H), 8.59 (d, J = 1.96 Hz, 1H), 8.47 (d, J = 2.45 Hz, 1H), 8.08 (d, J = 1.47 Hz, 1H), 7.03 (t, J = 9.54 Hz, 1H), −6.79–6.70 (m, 1H), 5.45 (br s, 2H).

#### Synthesis of (3-amino-2,4-difluorophenyl)-[5-bromo-1-(2,6-dichlorobenzoyl)­pyrrolo­[2,3*-b*]­pyridin-3-yl]­methanone (**A4**)

To a cooled (0 °C) solution of (3-amino-2,4-difluorophenyl)-(5-bromo-1*H-*pyrrolo­[2,3*-b*]­pyridin-3-yl)­methanone **A3** (11.9 g, 31.7 mmol) in tetrahydrofuran (338 mL) triethylamine (4.65 mL, 33.3 mmol) was added. 2,6-Dichlorobenzoyl chloride (4.60 mL, 32.1 mmol) was added to this solution dropwise. Afterward, 4-dimethylaminopyridine (4-DMAP, 0.194 g, 1.59 mmol) was added successively. The ice bath was removed, and the reaction was stirred at RT until complete consumption of the starting material (HPLC/TLC monitoring). The solvent volume was reduced to 1/10 and the residue was diluted with EtOAc (500 mL). The organic layer was washed twice with 1 M HCl (100 mL each), then with brine (100 mL) and dried afterward over sodium sulfate. The solvent was removed under reduced pressure and the residue redissolved in THF (66 mL). *n*-Heptane (330 mL) was added portion wise under vigorous stirring, stirring was continued for 1h at RT. The formed precipitate was filtered off and dried in vacuo, resulting in the beige (3-amino-2,4-difluorophenyl)-[5-bromo-1-(2,6-dichlorobenzoyl)­pyrrolo­[2,3*-b*]­pyridin-3-yl]­methanone (14.7 g, 26.5 mmol, 84% yield). ^1^H NMR (400 MHz, DMSO-*d*_6_) δ: 8.69 (d, J = 1.96 Hz, 1H), 8.53 (br s, 1H), 8.31 (br s, 1H), 7.67 (m, 3H), 7.11 (t, J = 9.29 Hz, 1H), 6.88–6.96 (m, 1H), 5.59 (s, 2H).

#### Synthesis of *N*-(3-(5-bromo-1-(2,6-dichlorobenzoyl)-1*H*-pyrrolo­[2,3-*b*]­pyridine- 3-carbonyl)-2,6-difluorophenyl)­propane-1 -sulfonamide (**A5**)

To a cooled (−10 °C, MeOH/ice) solution of (3-amino-2,4-difluorophenyl)-[5-bromo- 1-(2,6-dichlorobenzoyl)­pyrrolo­[2,3*-b*]­pyridin-3-yl]­methanone **A4** (8.00 g, 14.5 mmol) in THF (76.2 mL), TEA (15.5 mL, 111 mmol) was added. A solution of 1-propanesulfonyl chloride (3.58 mL, 31.8 mmol) in THF (3.6 mL) was added dropwise and the reaction mixture was stirred at −10 °C until TLC showed completion. To the cold solution, 2N NaOH (8 eq., 61 mL) was added, and the solution was warmed to RT. After TLC revealed complete hydrolysis of the disulfonamide, the mixture was acidified with 1 M HCI (pH = 2) and extracted twice with EtOAc. The combined organic layers were dried over sodium sulfate and the solvent was removed in vacuo. The residue was dissolved in EtOAc (50 mL) and treated with *n*-pentane until no further precipitate was formed. The solid was filtered off and dried in vacuo (main fraction of product). The filtrate was concentrated under reduced pressure and purified via flash chromatography using PE/EE 30% as eluent (second fraction of product). ^1^H NMR (400 MHz, DMSO-*d*_6_) δ: 9.72 (s, 1H), 8.69 (d, J = 2.1 Hz, 1H), 8.65 (s, 1H), 8.31 (s, 1H), 7.82 (dd, J = 14.3, 7.6 Hz, 1H), 7.66 (s, 3H), 7.42 (t, J = 8.8 Hz, 1H), 3.21–3.14 (m, 2H), 1.88–1.76 (m, 2H), 1.00 (t, J = 7.4 Hz, 3H).

#### Synthesis of *N*-(3-(1-(2,6-dichlorobenzoyl)-5-(4,4,5,5-tetramethyl-1,3,2- dioxaborolan-2-yl)-1 *H*-pyrrolo­[2,3-*b*]­pyridine-3-carbonyl)-2,6-difluorophenyl)­propane-1- sulfonamide (**A6**)

To a stirred solution of (3-(3-amino-2,4-difluorobenzoyl)-5-bromo-1*H*-pyrrolo­[2,3-*b*]­pyridin-1- yl)­(2,6-dichlorophenyl)­methanone **A5** (1 equiv, 7.0 g, 13.33 mmol) in dry 1,4-dioxane (100 mL) was added bis­(pinacolato)­diboron (1.20 equiv, 4.06 g, 16.00 mmol) followed by addition of potassium acetate (fused, 2.50 equiv, 3.28 g, 33.33 mmol). Pd­(dppf)­Cl_2_ x DCM (0.05 equiv, 0.54 g, 0.67 mmol) was added to the mixture, and stirring was continued at 60–80 °C for 3 h. The progress of the reaction was monitored by TLC (20% EtOAc in hexane) and LC-MS. After completion, the reaction mixture was cooled to RT and filtered through a pad of Celite. The resulting organic layer was concentrated under reduced pressure. The obtained crude product was dissolved in diethyl ether (200 mL), filtered and the organic layer was concentrated under reduced pressure. Trituration of the crude product with acetonitrile followed by filtration afforded (3-(3-amino-2,4-difluorobenzoyl)-5-(4,4,5,5-tetramethyl-1,3,2-dioxaborolan-2-yl)-1*H*-pyrrolo­[2,3-*b*]­pyridin-1-yl)­(2,6-dichlorophenyl) methanone 5 (7.0 g) as a light brown solid. ^1^H NMR (400 MHz, DMSO-*d*_6_) δ: 9.06 (d, J = 1.6 Hz, 1H), 8.48 (s, 1H), 8.37 (s, 1H), 7.77 (td, J = 8.9, 5.6 Hz, 1H), 7.45–7.32 (m, 3H), 7.09 (t, J = 8.5 Hz, 1H), 6.74 (s, 1H), 3.18–3.05 (m, 2H), 1.90 (dp, J = 10.3, 7.5 Hz, 2H), 1.33 (s, 12H), 1.07 (t, J = 7.4 Hz, 3H).

#### *N*-(2,6-Difluoro-3-(5-(2-methoxypyrimidin-5-yl)-1*H*-pyrrolo­[2,3-*b*]­pyridine-3-carbonyl)­phenyl)­propane-1-sulfonamide (**45**)

(2-methoxypyrimidin-5-yl)­boronic acid (29 mg, 0.19 mmol, 1.2 equiv), *N*-[3-[5-bromo-1-(2,6-dichlorobenzoyl)­pyrrolo­[2,3*-b*]­pyridine-3-carbonyl]-2,6-difluorophenyl]­propane-1-sulfonamide **A5** (144 mg, 0.23 mmol, 1.2 equiv), and potassium carbonate (79 mg, 0.57 mmol, 3 equiv) were suspended in 1,4-dioxane and water and degassed with argon. 1,1′-Bis­(diphenylphosphino)­ferrocene–dichloro palladium (1:1) (8 mg, 0.010 mmol, 0.05 equiv) was added, and the mixture was heated to 70–80 °C until completion of the reaction. The reaction mixture was filtered through a pad of Celite, flushed with EtOAc and solvent was evaporated. The residue was suspended in MeOH and potassium carbonate was added. The mixture was stirred at RT until complete deprotection. The reaction mixture was diluted with water and the pH was adjusted to 6 −7 with 1 M HCl, then the aqueous layer was extracted with EtOAc. The organic layer was separated and dried over sodium sulfate, the solvent was removed under reduced pressure and the product was purified by flash chromatography. ^1^H NMR (400 MHz, DMSO-*d*_6_) δ: 12.95 (s, 1H), 9.64 (s, 1H), 8.99 (s, 2H), 8.69 (d, J = 5.4 Hz), 8.09 (s, 1H), 7.64 (dd, J = 14.1, 7.4 Hz, 1H), 7.34 (t, J = 8.8 Hz, 1H), 4.00 (d, J = 20.7 Hz, 2H), 3.20- 3.06 (m, 2H) 1.79 (dd, J = 15.0, 7.5 Hz), 0.98 (t, J = 7.3 Hz, 3H).

#### *N*-(3-(5-(2-Cyclopropylpyrimidin-5-yl)-1*H-*pyrrolo­[2,3*-b*]­pyridine-3-carbonyl)-2,6-difluorophenyl)­propane-1-sulfonamide (**36**)

N-(3-(1-(2,6-dichlorobenzoyl)-5-(4,4,5,5-tetramethyl-1,3,2-dioxaborolan-2-yl)-1H-pyrrolo­[2,3-*b*]­pyridine-3-carbonyl)-2,6-difluorophenyl)­propane-1-sulfonamide **A6** (100 mg, 0.11 mmol), the 5-bromo-2-cyclopropylpyrimidine (35 mg, 0.18 mmol, 1.2 equiv), and potassium carbonate (61 mg, 0.44 mmol, 3eq) were suspended in 1,4-dioxane (1 mL) and water (0.5 mL) and degassed with argon. Pd­(dppf)­Cl_2_ (26 mg, 0.01 mmol, 0.05 equiv) was added, and the mixture was heated to 60–90 °C until completion. The solvent was evaporated and the residue dissolved in MeOH. Potassium carbonate was added and the mixture was stirred at RT. After complete deprotection, water was added and the pH was adjusted to ∼ 7 with aqueous HCl solution (1N). The aqueous layer was extracted with EtOAc, the combined organics were dried over sodium sulfate and the solvent removed in vacuo. The product was purified by flash chromatography to yield 35 mg (35%) of the target compound. ^1^H NMR (400 MHz, DMSO-*d*_6_) δ: 13.03 (s, 1H), 9.68 (s, 1H), 8.73 (dd, J = 6.6, 2.2 Hz, 2H), 8.11 (d, J = 1.6 Hz, 1H), 7.66 (dd, J = 14.6, 7.7 Hz, 1H), 7.36 (t, J = 8.4 Hz, 1H), 3.21–3.10 (m, 2H), 2.30 (ddd, J = 12.7, 8.0, 4.9 Hz, 1H), 1.87–1.74 (m, 2H), 1.17–1.04 (m, 4H), 1.00 (t, J = 7.4 Hz, 3H).

### Experimental *In Vivo* Models

All in vivo models were used to investigate the pharmacological properties of cp. **16** were performed at the University Hospital Tübingen and were approved by the German legal authority (Regierungspräsidium Tübingen, number M3/18).

The 2/3 hepatectomy studies except that to investigate cp. **16** were performed at A-Star institute, Singapore. The study was carried out according to the standard operating procedure in place at the test facility: Biological Resource Centre *BRC) A*STAR, Singapore. All procedures were performed in accordance with Singaporean laws. The animal study was approved by the A*STAR-IACUC IACUC#: 151054

The experimental acute liver injury (CCl_4_) model, except that to investigate cp. **16**, was performed by Physiogenex S.A.S., 31750 Escalquens (France) and has been carried out according to the standard operating procedure in place at the test facility. All procedures were performed in accordance with the *Guide for the Care and Use of Laboratory Animals (revised 1996)* and French laws

Pharmacokinetic studies in mice and rats were performed by Pharmacelsus GmbH, Saarbrücken (Germany). All experimental procedures were approved by and conducted in accordance with the regulations of the local Animal Welfare authorities (Landesamt für Gesundheit and Verbraucherschutz, Abteilung Lebensmittel- and Veterinärwesen, Saarbrücken, file no. 2.4.2.2-05/2018).

Details of the corresponding *in vivo* studies can be found in the

## Supplementary Material





## Notes

Experimental details for *in vitro* and *in vivo* studies, Supplementary figures on pharmacological data, experimental details of synthetic procedures and analytical data of compounds in this manuscript, HPLC and NMR plots for compounds **46**, **45**, **42**, **37**, **36**, and **16** (PDF)
Molecular formula strings of all compounds reported along with biological potency data (CSV)

## Abbreviations

ALT: alanine aminotransferase
APO: apo form (ligand-free protein structure)
Asp: aspartic acid
AST: aspartate aminotransferase
ATF2: activating transcription factor 2
AUC: area under the curve
BRaf: v-Raf murine sarcoma viral oncogene homologue B
CCl_4_: carbon tetrachloride
CLint: intrinsic clearance
Cmax: maximum plasma concentration
CYP: cytochrome P450
Cys: cysteine
DLM: dog liver microsomes
ELK1: ETS transcription factor ELK1
Gly: glycine
HAP1: human near-haploid cell line
hERG: human ether-a-go-go-related gene
HLM: human liver microsomes
IC_50_: half-maximal inhibitory concentration
IND: investigational new drug
INN: international nonproprietary name
i.p.: intraperitoneal
JNK: c-Jun N-terminal kinase
Ki67: proliferation marker protein *K*_i_-67
Lys: lysine
MAP: mitogen-activated protein
MAPK: mitogen-activated protein kinase
MASH: metabolic dysfunction-associated steatohepatitis
MEKK2: mitogen-activated protein kinase kinase kinase 2
MKK4: mitogen-activated protein kinase kinase 4
MKK7: mitogen-activated protein kinase kinase 7
MLM: mouse liver microsomes
NASH: nonalcoholic steatohepatitis
PoC: percent of control
p-MKK4: phosphorylated MKK4
RAF: rapidly accelerated fibrosarcoma kinase
RLM: rat liver microsomes
RNAi: RNA interference
SAPK: stress-activated protein kinase
SAR: structure–activity relationship
shRNA: short hairpin RNA
THR-β: thyroid hormone receptor beta
TUNEL: terminal deoxynucleotidyl transferase dUTP nick end labeling
VMF: Vemurafenib
ZAK: sterile alpha motif and leucine zipper containing kinase
