# Supplementary material for: Darizmetinib (HRX215): A Promising 1st-in-Class Liver Regenerating Drug in Phase 1b/2a Clinical Development, Targeting the Stress Signaling Protein Kinase MKK4
Source: J Med Chem. 2026 May 6;69(10):11687–704. doi: 10.1021/acs.jmedchem.6c00689 (PMC13224088; doi:10.1021/acs.jmedchem.6c00689)
Supplement: Supplementary file 1 [file jm6c00689_si_001.pdf]

SUPPORTING INFORMATION

Darizmetinib (HRX215): A Promising 1st-in-Class Liver Regenerating Drug in Phase 2 Clinical Development, Targeting the Stress Signalling Protein Kinase MKK4

*Wolfgang Albrecht[1], Roland Selig[1,2], Bent Pfaffenrot[2], Philip Kloevekorn[2], Michael Juchum[2] Stefan Zwirner[1,3], Sabrina Klotz[3], Lars Zender[3, 4, 5, 6], Stefan Laufer[2,4,5, 6 *]*

[1]HepaRegeniX GmbH, Tuebingen 72072, Germany

[2]Department of Pharmaceutical Chemistry, University of Tuebingen, Tuebingen 72076, Germany

[3]Department of Medical Oncology and Pneumology (Internal Medicine VIII), University Hospital Tuebingen, Tuebingen 72076, Germany

[4]iFIT Cluster of Excellence (EXC 2180) ''Image-guided and Functionally Instructed Tumor Therapies'', University of Tuebingen, Tuebingen 72076, Germany

[5]German Cancer Research Consortium (DKTK), German Cancer Research Center (DKFZ), Heidelberg 69120, Germany

[6]Tuebingen Center for Academic Drug Discovery & Development (TueCAD2), Tuebingen 72076, Germany

Table of Contents of Supporting Information

I: Details to binding affinity assay S2
II: Details to functional kinase inhibition assays S2
III: Details to Experimental *in vivo* models S3
IV: Synthetic procedures S6
V: NMR and HPLC Data of lead compounds S29
VI: Supplementary figures on pharmacological data S36
VII: References S45

I. Binding Affinity

Binding affinity of test compounds were measured using KINOMEscan™ Profiling Service at DiscoveRx Corporation, 42501 Albrae St. Fremont, CA 94538, USA which is based on a competition binding assay that quantitatively measures the ability of a compound to compete with an immobilized, active-site directed ligand. The technology is described in detail in Fabian et al.[S1] and in Karaman et al.[S2]

When tested at a single concentration, the affinity of compounds is quantified by determination Percent Control (%Ctrl or PoC), where lower numbers indicate stronger binding.

Percent Control Calculation:

$$\frac{test\ compound\ signal - positive\ control\ signal}{negative\ control\ signal - positive\ control\ signal} * 100$$

negative control = DMSO (100%Ctrl)

positive control = control compound (0%Ctrl)

II. Functional kinase inhibition assays

Novel developed MKK4 inhibitors were profiled in a radiometric protein kinase assay (33PanQinase® Activity Assay) by ProQinase according to their protocols. In short, the assay for all protein kinases contained 70 mM HEPES-NaOH pH 7.5, 3 mM $MgCl_2$, 3 mM $MnCl_2$, 3 µM Na orthovanadate, 1.2 mM DTT, 50 µg/ml PEG20000, ATP (variable concentrations, corresponding to the apparent ATP-Km of the respective kinase), [γ-^{33}P]-ATP (approx. 9 x 10^05 cpm per well), protein kinase and substrate. Incorporation of 33Pi was determined with a microplate scintillation counter (Microbeta, Wallac). IC_{50} values of MKK4 inhibitors were measured by screening 10 distinct concentrations in a semi-logarithmic dilution series ($1*10^{-04}$ M to $3*10^{-09}$ M) on different protein kinases (MKK4: 4.1 nM, JNK1: 2.3 nM, BRaf (wt): 14.5

nM). All protein kinases provided by ProQinase were expressed in Sf9 insect cells or in E. coli as recombinant GST-fusion proteins or His-tagged proteins, either as full-length or enzymatically active fragments. Affinity tags were removed from a number of kinases during purification. The purity of the protein kinases was examined by SDS-PAGE/Coomassie staining, the identity was checked by mass spectroscopy.

III. Experimental *in vivo* models

All *in vivo* models to investigate the pharmacological properties of cp. **16** were performed at the University Hospital Tübingen and were approved by the German legal authority (Regierungspräsidium Tübingen, number M3/18).

The 2/3 hepatectomy studies, except that to investigate cp. **16** were performed at A-star institute, Singapore. The study has been carried out according to the standard operating procedure in place at the test facility: Biological Resource Centre *BRC) A*STAR, Singapore. All procedures were performed in accordance with Singaporean laws. The animal study was approved by the A*STAR-IACUC IACUC#: 151054. Female C57BL/6N mice, 7-9 weeks old, were obtained from InVivos, Singapore. Animals were identified with ear marks and housed in enriched mouse cage (501 cm^2) throughout the experimental phase.

Animals' cages litters was changed after operation. Animals were housed in groups of 5 animals on a normal 12 hours light cycle (at 08:00pm lights off), 22 ± 2 °C and 50 ± 10 % relative humidity. During the at least 7 day acclimation phase, standard diet (Altromin, 1324 Best) and tap water were provided *ad libitum.*

After the acclimation period, mice were randomized based on their body weight and and test compounds or vehicle only were administered 12h and 1h prior to partial hepatectomy, which was performed according to Mitchell and Willenbring, 2008.[S4] 42 hours after partial

hepatectomy mice were sacrificed and livers were explanted and liver samples were dissected snap frozen, fixed in 4% PFA in PBS, or embedded in TissueTek and frozen.

Liver histology and quantification: Sections of TissueTek embedded and flash frozen liver were subjected to Ki67 (1:200, abcam ab15580) stainings. Alexa Fluor 594 and 488 (Thermo Fisher Scientific, R37117, R37121 and

R37120, R37116)-conjugated secondary antibodies were used for signal detection. Microscopic analyses were performed using Observer Z1 microscope (Zeiss). Five high power fields were counted on five liver sections from each mouse liver (200X, >200 counted cells per field). All statistical analyses were performed using GraphPad Prism 8 software. Statistical significance was calculated using the unpaired two-tailed student's t test.

The experimental acute liver injury (CCl_4) model, except that to investigate cp. **16**, was performed by Physiogenex S.A.S., 31750 Escalquens (France) and has been carried out according to the standard operating procedure in place at the test facility. All procedures were performed in accordance with the *Guide for the Care and Use of Laboratory Animals (revised 1996)* and French laws. Male C57BL/6N mice, 8-week old, were obtained from Charles River, France. Animals were identified with electronic chip or ear tags and housed in enriched and ventilated mouse cage GR500 (501 cm^2) throughout the experimental phase. Animals' cages litters were changed at least once a week. Animals were housed in groups of 5 animals on a normal 12 hours light cycle (at 08:00pm lights off), 22 ± 2 °C and 50 ± 10 % relative humidity. During the whole phase / acclimation phase standard diet (RM1 (E) 801492, SDS) and tap water were provided *ad libitum*.

After the acclimation period, mice were randomized into the respective treatment groups based on their body weight and received test items or respective vehicle at -12h and -1h before CCl_4 injection (5µL/g solution of CCl_4 in sunflower oil for a 0.25mL/kg CCl_4 dose). At 48 hours after CCl_4 i.p. injection, blood was collected on EDTA by retro-orbital bleeding and plasma was

isolated to measure plasma ALT and AST. Mice were then exsanguinated with sterile saline, liver was collected and weighted and liver samples were dissected from the left lateral lobe for immunohistochemistry (Caspase 3) or TUNEL assay or eventual additional analysis, as described in the table below.

Pharmacokinetic studies in mice and rats were performed by Pharmacelsus GmbH, Saarbrücken (Germany). All experimental procedures were approved by and conducted in accordance with the regulations of the local Animal Welfare authorities (Landesamt für Gesundheit und Verbraucherschutz, Abteilung Lebensmittel- und Veterinärwesen, Saarbrücken, file no. 2.4.2.2-05/2018).

Adult male C57BL/6JRj mice (8 weeks old at delivery) were purchased from Janvier Labs (France). The animals were housed in a temperature-controlled room (20-24°C) and maintained in a 12h light/12h dark cycle. Food and water were available *ad libitum* throughout the duration of the study. Test item formulations were administered by oral gavage. Seven serial samples samples were obtained from each mouse 15 min, 30 min, 1 h, 2 h, 3 h, 8 h and 24 h after oral administration of test items. At each of the designated time points, 20 µl blood was collected from the tail vein into Li-heparin 20 µl capillaries (Minivette POCT, SARSTEDT). The blood samples were transferred into polypropylene tubes and frozen on dry ice within 1-2 min of sampling. All samples were stored at -20°C until LC-MS analysis. Pharmacokinetic analysis was performed by applying a non-compartment model using the software Kinetica 5.0.

IV: Synthetic procedures

All reagents were of commercial grade and were used as received, without further purification.

NMR-spectra were measured on Bruker Avance 200 or 400 MHz NMR spectrometers. Chemical shifts (δ) for ^{1}H NMR spectra are reported in ppm relative to tetramethylsilane or the appropriate residual solvent peak. Multiplicities are given as singlet (s), doublet (d), doublet of doublets (dd), doublet of doublet of doublets (ddd), doublet of quartets (dq), doublet of triplets (dt), doublet of triplet of doublets (dtd), triplet (t), quartet (q), quintuplet (quin), multiplet (m), and broad (br).

Flash chromatography was carried out on Interchim PuriFlash XS420 flash chromatography system and Grace Davison Davisil LC60A 20e45 mm silica. Purity of the compounds was determined by HPLC analysis on Agilent 1100 Series Liquid Chromatograph using a Phenomenex Luna C8 150 x 4.6 mm, 5-mm column with gradient elution (MeOH/0.01M KH_2PO_4 buffer, pH 2.3, flow rate 1.5 mL/min) and detection at λ = 230 and 254 nm. All final compounds were determined with >95% purity if not stated otherwise. Mass spectra were recorded on Advion DCMS interface (ESI voltage: 3.50 kV, capillary voltage: 187 V, source voltage: 44 V, capillary temperature: 250°C, desolvation gas temperature: 250°C, gas flowrate: 5L/ min N2), elution of the spots with MeOH.

All final compounds reported were analysed using these analytical methods, purities were greater than 95% unless otherwise indicated.

The following substances have been previously described in the literature (compound numbers of the original publications are given)

Compound 2 synthesized acc. to Pfaffenrot et al.[S6], compound no. 10

Compound 3 synthesized acc. to Pfaffenrot et al.[S6], compound no. 9

Compound 7 synthesized acc. to patent literature[S12]

Compound 8 synthesized acc. to patent literature[S13]

Compound 9a synthesized acc. to Juchum et al.[S7], compound no. 16

Compound 9b synthesized acc. to Juchum et al.[S7], compound no. 11

Compound 9c synthesized acc. to Juchum et al.[S7], compound no. 19

Compound 11a synthesized acc. to Kloevekorn et al.[S5], compound no. 46

Compound 11b synthesized acc. to Pfaffenrot et al.[S6], compound no. 39

Compound 13 synthesized acc. to Pfaffenrot et al.[S6], compound no. 58

Compound 14 synthesized acc. to Pfaffenrot et al.[S6], compound no. 75

Compound 28 synthesized acc. to Pfaffenrot et al.[S6], compound no. 38

Compound 35 synthesized acc. to Kloevekorn et al.[S5], compound no. 57

Compound 12a synthesized acc. to Kloevekorn et al.[S5], compound no. 10

Compound 12b synthesized acc. to Kloevekorn et al.[S5], compound no. 11

Compound 12d synthesized acc. to Kloevekorn et al.[S5], compound no. 12

Compound 12f synthesized acc. to Kloevekorn et al.[S5], compound no. 13

Compound 12h synthesized acc. to Kloevekorn et al.[S5], compound no. 14

Compound 12j synthesized acc. to Kloevekorn et al.[S5], compound no. 18

Compound 12k synthesized acc. to Kloevekorn et al.[S5], compound no.19

Compound 12l synthesized acc. to Kloevekorn et al.[S5], compound no. 20

Compound 12m synthesized acc. to Kloevekorn et al.[S5], compound no. 16

Compound 12p synthesized acc. to Kloevekorn et al.[S5], compound no. 23

Compound 12q synthesized acc. to Kloevekorn et al.[S5], compound no. 22

Compound 20a synthesized acc. to Kloevekorn et al.[S5], compound no. 58

Compound 20b synthesized acc. to Kloevekorn et al.[S5], compound no. 59

Compound 20c synthesized acc. to Kloevekorn et al.[S5], compound no. 60

Compound 20d synthesized acc. to Kloevekorn et al.[S5], compound no. 61

Compound 20e synthesized acc. to Kloevekorn et al.[S5], compound no. 62

Synthesis of 7-azaindoles

General procedures for steps g, h, i (Scheme 1 manuscript)

Suzuki-Coupling of N-(3-(1-(2,6-dichlorobenzoyl)-5-(4,4,5,5-tetramethyl-1,3,2-dioxaborolan-2-yl)-1*H*-pyrrolo[2,3-*b*]pyridine-3-carbonyl)-2,6-difluorophenyl)propane-1-sulfonamide (A6) with corresponding aryl bromides (steps g, i)

N-(3-(1-(2,6-dichlorobenzoyl)-5-(4,4,5,5-tetramethyl-1,3,2-dioxaborolan-2-yl)-1*H*-pyrrolo[2,3-*b*]pyridine-3-carbonyl)-2,6-difluorophenyl)propane-1-sulfonamide, the corresponding aryl bromide (1.2-2 eq) and potassium fluoride or potassium carbonate (3eq) were suspended in 1,4-dioxane (0.9 mL) and water (2/1-4/1 v/v) and degassed with argon. Pd(dppf)Cl_2 (5 mol%) was added and the mixture was heated to 60-90°C until completion. The solvent was evaporated and the residue dissolved in methanol. Potassium carbonate was added and the mixture was stirred at RT. After complete deprotection, water was added and the pH was adjusted to ~7 with aqueous HCl solution (1N). The aqueous layer was extracted with EtOAc, the combined organics were dried over sodium sulfate and the solvent removed in vacuo. The product was purified by flash chromatography.

Suzuki-Coupling of aryl boronic acids with N-(3-(5-bromo-1-(2,6-dichlorobenzoyl)-1*H*-pyrrolo[2,3-*b*]pyridine-3-carbonyl)-2,6-difluorophenyl)propane-1-sulfonamide (A5) (steps h, i) The corresponding boronic acid (0.136 g, 0.871 mmol), N-[3-[5-bromo-1-(2,6-dichlorobenzoyl)pyrrolo[2,3-*b*]pyridine-3-carbonyl]-2,6-difluorophenyl]propane-1-sulfonamide (1.2-2 eq) and potassium carbonate (1-3 eq) were suspended in 1,4-dioxane and water (2/1-4/1 v/v) and degassed with argon. 1,1'-Bis(diphenylphosphino)ferrocene -dichloropalladium (1:1) (5 mol%) was added and the mixture was heated to 70-80°C until completetion of the reaction. The reaction mixture was filtered through a pad of Celite, flushed with EtOAc and solvent was evaporated. The residue was suspended in MeOH and potassium carbonat was added. The mixture was stirred at RT until complete deprotection. The reaction mixture was diluted with water and the pH was adjusted to 6 -7 with 1M HCl, then the aqueous layer was extracted with EtOAc. The organic layer was separated and dried over sodium sulfate, the solvent was removed under reduced pressure and the product was purified by flash chromatography.

N-(3-((5-(4-chlorophenyl)-1*H*-pyrrolo[2,3-*b*]pyridin-3-yl)methyl)-2,4-difluorophenyl)propane-1-sulfonamide (10a)

Step a: Vemurafenib was dissolved in THF and cooled to 0°C. 3 equivalents of lithium aluminium hydride were added to the reaction mixture under stirring. After 30 minutes the mixture was slowly warmed to RT and further stirred for 2 hours. The reaction mixture was quenched with water and extracted with EtOAc. The organic layer was evaporated and the crude product was roughly purified via column chromatography (eluent EtOAc/n-hexane 2/1).

Step b: The obtained product from step a was dissolved in dichloromethane and cooled to 0°C. Then 1.5eq triethylsilane was added, the reaction mixture was stirred for 5 hours, then trifluoromethanesulfonic acid was added and the mixture was warmed to RT and stirred for

further 16h. After competition of the reaction, water was added together with DCM. The organic layer was separated and evaporated and the obtained crude product was purified by flash chromatography. ^{1}H NMR (400 MHz, DMSO-d_6) δ: 11.60 (s, 1H), 9.56 (s, 1H), 8.50 (s, 1H), 8.15 (s, 1H), 7.72 (d, *J* = 8.3 Hz, 2H), 7.54 (d, *J* = 8.4 Hz, 2H), 7.36 – 7.25 (m, 1H), 7.22 (s, 1H), 7.10 (t, *J* = 8.6 Hz, 1H), 3.05 – 2.94 (m, 2H), 1.67 (dd, *J* = 15.2, 7.6 Hz, 2H), 0.88 (t, *J* = 7.5 Hz, 3H).

N-(3-((5-(4-chlorophenyl)-1*H*-pyrrolo[2,3-*b*]pyridin-3-yl)thio)-2,4-difluorophenyl)propane-1-sulfonamide (10b)

Synthesis according to related patent literature.[S8]

^{1}H NMR (400 MHz, DMSO-d_6) δ: 12.32 (d, *J* = 2.1 Hz, 1H), 9.63 (s, 1H), 8.57 (d, *J* = 2.1 Hz, 1H), 8.08 (d, *J* = 2.0 Hz, 1H), 7.95 (d, *J* = 2.7 Hz, 1H), 7.74 – 7.67 (m, 2H), 7.58 – 7.52 (m, 2H), 7.36 (td, *J* = 8.9, 5.9 Hz, 1H), 7.17 – 7.09 (m, 1H), 2.98 – 2.90 (m, 2H), 1.61 (dq, *J* = 15.0, 7.5 Hz, 2H), 0.82 (t, *J* = 7.4 Hz, 3H).

N-(3-((5-(4-chlorophenyl)-1*H*-pyrrolo[2,3-*b*]pyridin-3-yl)sulfinyl)-2,4-difluorophenyl)propane-1-sulfonamide (10c)

Synthesis according to related patent literature.[S8] ^{1}H NMR (400 MHz, DMSO-d_6) δ: 12.79 (s, 1H), 9.80 (s, 1H), 8.67 (d, *J* = 2.2 Hz, 1H), 8.22 (d, *J* = 2.1 Hz, 2H), 7.72 – 7.66 (m, 2H), 7.59 – 7.53 (m, 2H), 7.25 (t, *J* = 9.6 Hz, 1H), 3.04 – 2.95 (m, 2H), 1.61 (dq, *J* = 15.1, 7.5 Hz, 2H), 0.84 (t, *J* = 7.4 Hz, 2H).

N-(3-((5-(4-chlorophenyl)-1*H*-pyrrolo[2,3-*b*]pyridin-3-yl)sulfonyl)-2,4-difluorophenyl)propane-1-sulfonamide (10d)

Synthesis according to related patent literature.[S8] ^{1}H NMR (400 MHz, DMSO-d_6) δ: 13.81 – 13.46 (m, 1H), 9.79 (s, 1H), 9.79 (s, 1H), 8.66 (d, *J* = 1.3 Hz, 1H), 8.11 (s, 1H), 7.83 (t, *J* = 3.6 Hz, 1H), 7.75 – 7.67 (m, 2H), 7.64 – 7.51 (m, 2H), 7.27 (t, *J* = 9.2 Hz, 1H), 3.07 – 2.96 (m, 2H), 1.62 (dq, *J* = 14.8, 7.4 Hz, 2H), 0.89 – 0.81 (m, 2H).

(E)-N-(3-(2-(5-(4-chlorophenyl)-1*H*-pyrrolo[2,3-*b*]pyridin-3-yl)vinyl)-2,4-difluorophenyl)propane-1-sulfonamide (10e)

To a suspension of N-(3-((bromotriphenyl-l5-phosphaneyl)methyl)-2,4-difluorophenyl)propane-1-sulfonamide (2eq) in dry THF was added n-BuLi (3.3eq) at −10 °C under nitrogen atmosphere. The mixture was stirred at −10 °C for 25 minutes, then a solution of 5-(4-chlorophenyl)-1-((2-(trimethylsilyl)ethoxy)methyl)-1*H*-pyrrolo[2,3-*b*]pyridine-3-carbaldehyde (1 g) in dry THF was added. The mixture was stirred at −10 °C for 40 minutes, then warmed to RT and stirred further overnight. The mixture was quenched with water, concentrated in vacuo, and the residue was extracted with EtOAc. The combined organic layers were dried over anhydrous sodium sulfate and concentrated. The residue was purified by a silica gel column chromatography. The obtained roughly purified residue was taken up in a 1/1 mixture of DCM and trifluoro acetic acid at RT and stirred for 3 h. Water was added to the reaction mixture, the mixture was neutralized with aq. NaOH, then extracted with EtOAc. After evaporation, the residue was treated with a MeOH/ aq conc. NH_3 (50/50 v/v) solution for 3h at RT. The mixture was neutralized with aq HCl, then extracted with EtOAc and purified via flash chromatography. ^{1}H NMR (400 MHz, DMSO-d_6) δ: 12.16 (s, 1H), 9.61 (s, 1H), 8.59 (d, *J* = 1.9 Hz, 1H), 8.48 (d, *J* = 2.0 Hz, 1H), 8.02 (s, 1H), 7.82 (d, *J* = 8.5 Hz, 2H), 7.61 – 7.52 (m, 3H), 7.26 (dd, *J* = 14.4, 8.6 Hz, 1H), 7.18 – 7.10 (m, 1H), 7.05 (d, *J* = 16.9 Hz, 1H), 3.13 – 3.04 (m, 2H), 1.76 (dd, *J* = 15.2, 7.6 Hz, 2H), 0.99 (t, *J* = 7.4 Hz, 3H).

N-(3-(2-(5-(4-chlorophenyl)-1*H*-pyrrolo[2,3-*b*]pyridin-3-yl)-2-oxoacetyl)-2,4-difluorophenyl)propane-1-sulfonamide (10f)

300 mg SEM protected N-(3-(2-(5-(4-chlorophenyl)-1*H*-pyrrolo[2,3-*b*]pyridin-3-yl)-1,2-dihydroxyethyl)-2,4-difluorophenyl)propane-1-sulfonamide (10g) was dissolved in dioxane and 20 eq DDQ were added. The mixture was heated to 110°C for 18 hours, then cooled to RT. After adding water and EtOAc, the organic layer was separated, the aq layer was extracted another two times with EtOAc. The combined organic layers were dried over Na_2SO_4, then evaporated. The crude product was purified via flash chromatography to obtain 170 mg of the SEM protected product which was deprotected as described for 10e to yield 92 mg of the product. ^1H NMR (400 MHz, DMSO-d_6) δ: 13.21 (s, 1H), 9.85 (s, 1H), 9.02 – 8.37 (m, 3H), 7.97 – 7.46 (m, 4H), 7.32 (s, 1H), 3.19 – 3.01 (m, 2H), 1.82 – 1.60 (m, 2H), 0.95 (s, 3H).

N-(3-(2-(5-(4-chlorophenyl)-1*H*-pyrrolo[2,3-*b*]pyridin-3-yl)-1,2-dihydroxyethyl)-2,4-difluorophenyl)propane-1-sulfonamide (10g)

SEM protected (E)-N-(3-(2-(5-(4-chlorophenyl)-1*H*-pyrrolo[2,3-*b*]pyridin-3-yl)vinyl)-2,4-difluorophenyl)propane-1-sulfonamide (10e) was dissolved in a mixture of THF and water, then OsO_4 (0.5eq), NMO (5eq) and 1eq methane sulfonamide were added at RT and stirred for 18 hours. Ongoing deprotection and purification was performed as described for 10e. ^1H NMR (400 MHz, DMSO-d_6) δ: 11.47 (s, 1H), 9.41 (s, 1H), 8.42 (d, J = 2.2 Hz, 1H), 8.06 (d, J = 2.1 Hz, 1H), 7.65 (d, J = 8.6 Hz, 1H), 7.55 (d, J = 8.6 Hz, 1H), 7.16 (d, J = 2.4 Hz, 1H), 7.16 – 7.09 (m, 1H), 6.88 (t, J = 9.4 Hz, 1H), 5.87 (d, J = 3.9 Hz, 1H), 5.57 (d, J = 3.7 Hz, 1H), 5.21 (d, J = 3.3 Hz, 1H), 2.71 – 2.59 (m, 2H), 1.56 (dd, J = 12.5, 5.2 Hz, 2H), 0.85 (d, J = 5.6 Hz, 2H).

5-(4-chlorophenyl)-N-(2,6-difluoro-3-(propylsulfonamido)phenyl)-1*H*-pyrrolo[2,3-*b*]pyridine-3-carboxamide (10h)

Synthesis according to related patent literature.[S8] ^{1}H NMR (400 MHz, DMSO-d_6) δ: 12.48 (s, 1H), 9.85 (s, 1H), 9.71 (s, 1H), 8.63 (s, 1H), 8.46 (s, 1H), 7.79 – 7.72 (m, 1H), 7.59 – 7.51 (m, 1H), 7.41 – 7.32 (m, 1H), 7.21 (t, *J* = 9.4 Hz, 1H), 3.13 – 3.04 (m, 2H), 1.75 (dt, *J* = 15.1, 7.7 Hz, 2H), 0.98 (t, *J* = 7.4 Hz, 2H).

N-(5-(4-chlorophenyl)-1*H*-pyrrolo[2,3-*b*]pyridin-3-yl)-2,6-difluoro-3-(propylsulfonamido)benzamide (10i)

Synthesis according to related patent literature.[S8] ^{1}H NMR (400 MHz, DMSO-d_6) δ: 11.69 (s, 1H), 11.03 (s, 1H), 9.82 (s, 1H), 8.58 (d, *J* = 2.2 Hz, 1H), 8.50 (d, *J* = 2.1 Hz, 1H), 8.00 (d, *J* = 2.5 Hz, 1H), 7.73 – 7.69 (m, 1H), 7.58 – 7.50 (m, 2H), 7.24 (t, *J* = 8.9 Hz, 1H), 3.14 – 3.05 (m, 2H), 1.75 (dt, *J* = 15.2, 7.6 Hz, 2H), 0.98 (t, *J* = 7.4 Hz, 2H).

***N*-(3-(5-(4-chlorophenyl)-1*H*-pyrrolo[2,3-*b*]pyridine-3-carbonyl)-2,4-difluorophenyl)cyclopropanesulfonamide (12c)**

Synthesis according to related patent literature[S11] ^{1}H NMR (400 MHz, DMSO-d_6) δ: 13.02 (s, 1H), 9.76 (s, 1H), 8.71 (d, J = 2.2 Hz, 1H), 8.65 (s, 1H), 8.22 (s, 1H), 7.79 (d, J = 8.5 Hz, 2H), 7.65 – 7.52 (m, 3H), 7.29 (t, J = 8.4 Hz, 1H), 2.76 – 2.68 (m, 1H), 0.92 (dt, J = 16.4, 6.7 Hz, 4H).

***N*-(3-(5-(4-chlorophenyl)-1*H*-pyrrolo[2,3-*b*]pyridine-3-carbonyl)-2,4-difluorophenyl)butane-1-sulfonamide (12d)**

Synthesis according to related patent literature[S11] ^{1}H NMR (400 MHz, DMSO-d_6) δ: 13.03 (s, 1H), 9.78 (s, 1H), 8.71 (d, J = 2.1 Hz, 1H), 8.64 (s, 1H), 8.26 (s, 1H), 7.79 (d, J = 8.5 Hz, 2H), 7.64 – 7.51 (m, 3H), 7.29 (t, J = 8.6 Hz, 1H), 3.17 – 3.07 (m, 2H), 1.70 (dt, J = 15.2, 7.6 Hz, 2H), 1.43 – 1.30 (m, 2H), 0.85 (t, J = 7.3 Hz, 3H).

N-(3-(5-(4-chlorophenyl)-1H-pyrrolo[2,3-b]pyridine-3-carbonyl)-2,4-difluorophenyl)-2-methylpropane-1-sulfonamide (12e)

Synthesis according to Kloevekorn et al.[S5] ^{1}H NMR (400 MHz, DMSO-d_6) δ: 13.03 (s, 1H), 9.79 (s, 1H), 8.71 (d, J = 2.2 Hz, 1H), 8.65 (s, 1H), 8.26 (d, J = 1.6 Hz, 1H), 7.79 (d, J = 8.5 Hz, 1H), 7.64 – 7.53 (m, 1H), 7.29 (t, J = 8.6 Hz, 1H), 3.05 (d, J = 6.4 Hz, 1H), 2.26 – 2.09 (m, 1H), 1.02 (d, J = 6.7 Hz, 1H).

N-(3-(5-(4-chlorophenyl)-1*H*-pyrrolo[2,3-*b*]pyridine-3-carbonyl)-2,4-difluorophenyl)-2-methylpropane-1-sulfonamide (12f)

The compound was synthesized in analogy to Kloevekorn et al.[S5] using 2,6-difluoro-3-((2-methylpropyl)sulfonamido)benzoic acid. ^{1}H NMR (DMSO-d_6, 400 MHz, ppm) δ: 13.03 (s, 1H), 9.79 (s, 1H), 8.71 (d, J = 2.2 Hz, 1H), 8.65 (s, 1H), 8.26 (d, J = 1.6 Hz, 1H), 7.79 (d, J = 8.5 Hz, 1H), 7.64 – 7.53 (m, 1H), 7.29 (t, J = 8.6 Hz, 1H), 3.05 (d, J = 6.4 Hz, 1H), 2.26 – 2.09 (m, 1H), 1.02 (d, J = 6.7 Hz, 1H).

N-(3-(5-(4-chlorophenyl)-1*H*-pyrrolo[2,3-*b*]pyridine-3-carbonyl)-2,4-difluorophenyl)-3-methylbutane-1-sulfonamide (12g)

The compound was synthesized in analogy to Kloevekorn et al.[S5] using 2,6-difluoro-3-((3-methylbutyl)sulfonamido)benzoic acid. ^{1}H NMR (DMSO-d_6, 400 MHz, ppm) δ: 13.03 (s, 1H),

9.79 (s, 1H), 8.71 (d, J = 1.9 Hz, 1H), 8.64 (s, 1H), 8.26 (s, 1H), 7.79 (d, J = 8.4 Hz, 2H), 7.67 – 7.47 (m, 3H), 7.29 (t, J = 8.6 Hz, 1H), 3.23 – 3.02 (m, 2H), 1.69 – 1.51 (m, 3H), 0.83 (d, J = 5.7 Hz, 6H).

***N*-(3-(5-(4-chlorophenyl)-1*H*-pyrrolo[2,3-*b*]pyridine-3-carbonyl)-2,4-difluorophenyl)hexane-1-sulfonamide (12h)**

Synthesis according to related patent literature[S11] ^{1}H NMR (400 MHz, DMSO-d_6) δ: 13.04 (s, 1H), 9.78 (s, 1H), 8.71 (d, J = 2.1 Hz, 1H), 8.63 (s, 1H), 8.25 (d, J = 1.8 Hz, 1H), 7.79 (d, J = 8.4 Hz, 2H), 7.66 – 7.50 (m, 3H), 7.29 (t, J = 8.7 Hz, 1H), 3.18 – 3.07 (m, 2H), 1.76 – 1.63 (m, 2H), 1.40 – 1.27 (m, 2H), 1.26 – 1.15 (m, 4H), 0.79 (t, J = 6.7 Hz, 3H).

N-(3-(5-(4-chlorophenyl)-1*H*-pyrrolo[2,3-*b*]pyridine-3-carbonyl)-2,4-difluorophenyl)cyclohexanesulfonamide (12i)

The compound was synthesized in analogy to Kloevekorn et al.[S5] using cyclohexanesulfonyl chloride and (3-amino-2,6-difluorophenyl)(5-(4-chlorophenyl)-1*H*-pyrrolo[2,3-*b*]pyridin-3-yl)methanone. ^{1}H NMR (400 MHz, DMSO-d_6) δ: 13.05 (s, 1H), 9.76 (s, 1H), 8.72 (d, J = 2.1 Hz, 1H), 8.63 (s, 1H), 8.24 (s, 1H), 7.79 (d, J = 8.5 Hz, 2H), 7.66 – 7.51 (m, 3H), 7.28 (t, J = 8.5 Hz, 1H), 3.05 (t, J = 11.7 Hz, 1H), 2.09 (d, J = 11.2 Hz, 2H), 1.76 (d, J = 12.7 Hz, 2H), 1.60 (d, J = 12.3 Hz, 1H), 1.40 (qd, J = 12.1, 2.2 Hz, 2H), 1.31 – 1.03 (m, 3H).

***N*-(3-(5-(4-chlorophenyl)-1*H*-pyrrolo[2,3-*b*]pyridine-3-carbonyl)-2,4-difluorophenyl)benzenesulfonamide (12j)**

Synthesis according to related patent literature[S11] ^{1}H NMR (400 MHz, DMSO-d_6) δ: 13.03 (s, 1H), 10.29 (s, 1H), 8.71 (d, J = 2.2 Hz, 1H), 8.60 (s, 1H), 7.86 (s, 1H), 7.78 (d, J = 8.5 Hz, 2H),

7.73 (d, J = 7.2 Hz, 2H), 7.66 (t, J = 7.4 Hz, 1H), 7.61 – 7.53 (m, 4H), 7.45 (td, J = 8.9, 6.0 Hz, 1H), 7.26 (t, J = 8.3 Hz, 1H).

_N_-(3-(5-(4-chlorophenyl)-1_H_-pyrrolo[2,3-_b_]pyridine-3-carbonyl)-2,4-difluorophenyl)-2-hydroxypropane-1-sulfonamide (12o)

The compound was synthesized in analogy to Kloevekorn et al.[S5] using 2-oxopropane-1-sulfonyl chloride. ^{1}H NMR (400 MHz, DMSO-d_6) δ: 13.03 (s, 1H), 10.06 (s, 1H), 8.72 (d, *J* = 2.1 Hz, 1H), 8.66 (s, 1H), 8.25 (s, 1H), 7.80 (d, *J* = 8.4 Hz, 2H), 7.80 (d, *J* = 8.4 Hz, 2H), 7.59 (t, *J* = 10.2 Hz, 2H), 7.59 (t, *J* = 10.2 Hz, 2H), 7.30 (t, *J* = 8.6 Hz, 1H), 7.30 (t, *J* = 8.6 Hz, 1H), 4.45 (s, 2H), 2.27 (s, 3H).

_N_-(3-(5-(4-chlorophenyl)-1_H_-pyrrolo[2,3-_b_]pyridine-3-carbonyl)-2,4-difluorophenyl)-1-(2-fluorophenyl)methanesulfonamide (12p)

Synthesis according to related patent literature[S11] ^{1}H NMR (400 MHz, DMSO) δ: 13.04 (s, 1H), 10.02 (s, 1H), 8.72 (d, J = 2.1 Hz, 1H), 8.67 (s, 1H), 8.21 (s, 1H), 7.80 (d, J = 8.5 Hz, 2H), 7.61 – 7.52 (m, 3H), 7.51 – 7.38 (m, 2H), 7.31 – 7.15 (m, 3H), 4.60 (s, 2H).

N-(3-(5-(4-chlorophenyl)-1_H_-pyrrolo[2,3-_b_]pyridine-3-carbonyl)-2,4-difluorophenyl)-2-oxopropane-1-sulfonamide (12n)

Dichlorobenzoyl protected N-(3-(5-(4-chlorophenyl)-1*H*-pyrrolo[2,3-*b*]pyridine-3-carbonyl)-2,4-difluorophenyl)-2-hydroxypropane-1-sulfonamide (12o) was dissolved in a mixture of THF/MeOH and cooled to 0°C, then $NaBH_4$ (5eq) was added under stirring, after 30 minutes, the reaction mixture was quenched with water and the product was extracted with EtOAc. The crude product was deprotected with aq. ammonia in a mixture of MeOH/THF at RT within 4 h.

The mixture was neutralized with aq. HCl, extracted with EtOAc, then purified via flash chromatography. ^{1}H NMR (400 MHz, DMSO-d_6) δ: 13.02 (s, 1H), 9.66 (s, 1H), 8.71 (d, *J* = 2.1 Hz, 1H), 8.64 (s, 1H), 8.22 (s, 1H), 7.80 (d, *J* = 8.5 Hz, 2H), 7.66 – 7.53 (m, 3H), 7.28 (t, *J* = 8.7 Hz, 1H), 5.01 (d, *J* = 4.7 Hz, 1H), 4.12 (d, *J* = 5.7 Hz, 1H), 3.23 (dd, *J* = 10.2, 6.0 Hz, 2H), 1.20 (d, *J* = 6.3 Hz, 3H).

N-(3-(5-(4-chlorophenyl)-1H-pyrrolo[2,3-b]pyridine-3-carbonyl)-2,4-difluorophenyl)-1-(4-fluorophenyl)methanesulfonamide (12r)

Synthesis according to Kloevekorn et al.[S5] ^{1}H NMR (400 MHz, DMSO-d_6) δ: 13.04 (s, 1H), 9.83 (s, 1H), 8.72 (s, 1H), 8.67 (s, 1H), 8.24 (s, 1H), 7.80 (d, J = 7.8 Hz, 2H), 7.61 – 7.48 (m, 3H), 7.46 – 7.39 (m, 2H), 7.25 (t, J = 8.8 Hz, 1H), 7.19 (t, J = 8.4 Hz, 2H), 4.55 (s, 2H).

4-(3-(2,6-difluoro-3-((phenylmethyl)sulfonamido)benzoyl)-1*H*-pyrrolo[2,3-*b*]pyridin-5-yl)benzenesulfonamide (15)

The compound was synthesized in analogy to Kloevekorn et al.[S5] using N-(3-(5-bromo-1*H*-pyrrolo[2,3-*b*]pyridine-3-carbonyl)-2,4-difluorophenyl)-1-phenylmethanesulfonamide and (4-sulfamoylphenyl)boronic acid.^{1}H NMR (DMSO-d6, 400 MHz, ppm) δ: 13.10 (s, 1H), 9.85 (s, 1H), 8.80 (s, 1H), 8.75 (s, 1H), 8.27 (d, J = 1.8 Hz, 1H), 7.98 (q, J = 8.5 Hz, 4H), 7.52 (dd, J = 14.9, 9.0 Hz, 1H), 7.46 (s, 2H), 7.43 – 7.32 (m, 5H), 7.25 (t, J = 8.7 Hz, 1H), 4.55 (s, 2H).

N-(3-(5-(2-cyclopropylpyrimidin-5-yl)-1*H*-pyrrolo[2,3-*b*]pyridine-3-carbonyl)-2,4-difluorophenyl)-1-phenylmethanesulfonamide (16)

The compound was synthesized in analogy to Kloevekorn et al.[S5] ^{1}H NMR (DMSO-d_6, 400 MHz, ppm) δ: 9.93 (s, 1H), 8.88 (s, 1H), 8.75 (d, J = 1.8 Hz, 1H), 8.34 (s, 1H), 7.66 (s, 3H),

7.60 (dd, J = 14.6, 8.6 Hz, 1H), 7.41 (d, J = 3.7 Hz, 2H), 7.36–7.33 (m, 3H), 7.28 (t, J = 9.0 Hz, 1H), 4.57 (s, 2H).

N-(2,4-difluoro-3-(5-(2-methoxypyrimidin-5-yl)-1*H*-pyrrolo[2,3-*b*]pyridine-3-carbonyl)phenyl)-1-phenylmethanesulfonamide (17)

The compound was synthesized in analogy to Kloevekorn et al.[S5] ^{1}H NMR (600 MHz, DMSO-d_6) δ: 13.07 (d, J = 1.5 Hz, 1H), 9.82 (s, 1H), 9.02 (s, 2H), 8.74 (d, J = 2.2 Hz, 1H), 8.70 (s, 1H), 8.24 (d, J = 2.1 Hz, 1H), 7.51 (td, J = 9.0, 5.8 Hz, 1H), 7.40–7.38 (m, 2H), 7.36–7.33 (m, 3H), 7.24 (t, J = 8.5 Hz, 1H), 4.54 (s, 2H), 4.00 (s, 3H).

N-(3-(5-(4-chlorophenyl)-1*H*-pyrrolo[2,3-*b*]pyridine-3-carbonyl)-2,6-difluorophenyl)propane-1-sulfonamide (20f)

The compound was synthesized in analogy to Kloevekorn et al.[S5] ^{1}H NMR (400 MHz, DMSO-d_6) δ: 12.92 (s, 1H), 9.64 (s, 1H), 8.37 (dd, *J* = 10.3, 2.1 Hz, 2H), 8.09 (s, 1H), 7.69 – 7.60 (m, 1H), 7.46 (s, 1H), 7.41 – 7.26 (m, 3H), 3.17 – 3.11 (m, 2H), 1.81 (dd, *J* = 15.1, 7.6 Hz, 2H), 1.00 (t, *J* = 7.4 Hz, 3H).

N-(3-(5-(4-chlorophenyl)-1*H*-pyrrolo[2,3-*b*]pyridine-3-carbonyl)-2,5-difluorophenyl)propane-1-sulfonamide (20g)

The compound was synthesized in analogy to Kloevekorn et al.[S5] ^{1}H NMR (400 MHz, DMSO-d_6) δ: 12.95 (s, 1H), 10.09 (s, 1H), 8.68 (dt, J = 11.5, 5.8 Hz, 2H), 8.18 (s, 1H), 7.77 (t, J = 10.0 Hz, 2H), 7.58 (d, J = 8.5 Hz, 2H), 7.43 (ddd, J = 9.3, 6.0, 3.1 Hz, 1H), 7.34 – 7.25 (m, 1H), 3.25 – 3.18 (m, 2H), 1.75 (dq, J = 14.9, 7.4 Hz, 2H), 0.98 (t, J = 7.4 Hz, 3H).

N-(3-(5-(4-chlorophenyl)-1*H*-pyrrolo[2,3-*b*]pyridine-3-carbonyl)-2,5,6-trifluorophenyl)propane-1-sulfonamide (22)

The compound was synthesized in analogy to Kloevekorn et al.[S5] ^{1}H NMR (400 MHz, DMSO-d_6) δ: 8.66 (d, J = 2.0 Hz, 2H), 8.12 (s, 1H), 7.79 – 7.65 (m, 3H), 7.60 – 7.51 (m, 2H), 3.20 – 3.10 (m, 2H), 1.84 – 1.69 (m, 2H), 0.96 (t, J = 7.4 Hz, 3H).

N-(3-(5-(4-chlorophenyl)-1*H*-pyrrolo[2,3-*b*]pyridine-3-carbonyl)-2,4,5-trifluorophenyl)propane-1-sulfonamide (23)

The compound was synthesized in analogy to Kloevekorn et al.[S5] ^{1}H NMR (400 MHz, DMSO-d_6) δ: 13.09 (s, 1H), 10.03 (s, 1H), 8.70 (d, J = 18.1 Hz, 2H), 8.41 (s, 1H), 7.81 (d, J = 8.1 Hz, 2H), 7.61 (dd, J = 25.9, 8.9 Hz, 3H), 3.24 – 3.13 (m, 2H), 1.74 (d, J = 7.3 Hz, 2H), 0.97 (t, J = 7.2 Hz, 3H).

N-(3-(5-(4-chlorophenyl)-1*H*-pyrrolo[2,3-*b*]pyridine-3-carbonyl)-2,4,6-trifluorophenyl)propane-1-sulfonamide (24)

The compound was synthesized in analogy to Kloevekorn et al.[S5] ^{1}H NMR (400 MHz, DMSO-d_6) δ: 13.04 (s, 1H), 9.63 (s, 1H), 8.72 (d, J = 2.2 Hz, 1H), 8.65 (s, 1H), 8.29 (s, 1H), 7.80 (d, J = 8.5 Hz, 2H), 7.55 (dd, J = 20.8, 8.6 Hz, 3H), 3.18 – 3.09 (m, 2H), 1.79 (dq, J = 14.9, 7.4 Hz, 2H), 0.98 (t, J = 7.4 Hz, 3H).

N-(3-(5-(2-chloro-4-methoxyphenyl)-1*H*-pyrrolo[2,3-*b*]pyridine-3-carbonyl)-2,6-difluorophenyl)propane-1-sulfonamide (26) The compound was synthesized in analogy to

step h ^{1}H NMR (600 MHz, DMSO-d_6) δ: 12.91 (s, 1H), 9.64 (s, 1H), 8.49 (d, J = 2.2 Hz, 1H), 8.33 (d, J = 2.2 Hz, 1H), 8.08 (s, 1H), 7.65 (dd, J = 14.4, 7.7 Hz, 1H), 7.46 (t, J = 8.8 Hz, 1H), 7.37 (t, J = 8.5 Hz, 2H), 3.85 (s, 1H), 3.10 – 3.03 (m, 2H), 1.86 – 1.77 (m, 2H), 1.00 (t, J = 7.4 Hz, 3H). Calculated mass: 519.1 MS(ESI): 517.9 for $[M+H]^+$

N-(3-(5-(2-cyclopropylpyrimidin-5-yl)-1*H*-pyrrolo[2,3-*b*]pyridine-3-carbonyl)-2,6-difluorophenyl)-1-phenylmethanesulfonamide (30) The compound was synthesized in analogy to step g ^{1}H NMR (400 MHz, DMSO-d_6) δ: 12.99 (s, 1H), 9.02 (s, 1H), 8.73 (s, 2H), 8.12 (s, 1H), 7.69-7.59 (m, 2H), 7.47-7.42 (m, 2H), 4.52 (s, 2H) 2.35-2.22 (m, 1H), 1.15-1.02 (m, 4H) Calculated mass: 545.13MS(ESI): 545.95 for $[M+H]^+$

N-(3-(5-(2-cyclopropylpyrimidin-5-yl)-1H-pyrrolo[2,3-b]pyridine-3-carbonyl)-2,6-difluorophenyl)methanesulfonamide (32)

Synthesis according to related patent literature.[S8]

^{1}H NMR (400 MHz, DMSO-d_6) δ: 12.97 (s, 1H), 9.72 (s, 1H), 9.01 (s, 2H), 8.72 (s, 2H), 8.12 (d, J = 2.6 Hz, 1H), 7.66 (q, J = 7.6 Hz, 1H), 7.36 (t, J = 8.9 Hz, 1H), 3.12 (s, 3H), 2.27 (td, J = 8.0, 4.0 Hz, 1H), 1.13 – 1.01 (m, 4H).

N-(3-(5-(2-cyclopropylpyrimidin-5-yl)-1H-pyrrolo[2,3-b]pyridine-3-carbonyl)-2,6-difluorophenyl)-3,3,3-trifluoropropane-1-sulfonamide (33)

Synthesis according to related patent literature.[S8] ^{1}H NMR (400 MHz, DMSO-d_6) δ: 12.97 (d, J = 3.1 Hz, 1H), 10.03 (s, 1H), 9.01 (s, 2H), 8.75–8.66 (m, 2H), 8.14 (d, J = 2.6 Hz, 1H), 7.69 (td, J = 8.2, 6.1 Hz, 1H), 7.39 (t, J = 8.8 Hz, 1H), 3.52–3.43 (m, 2H), 2.84 (dd, J = 11.6, 8.5, 4.4 Hz, 2H), 2.28 (t, J = 7.8, 4.9 Hz, 1H), 1.12–1.04 (m, 4H, 4H).

N-(3-(5-(2-cyclopropylpyrimidin-5-yl)-1H-pyrrolo[2,3-b]pyridine-3-carbonyl)-2,6-difluorophenyl)ethanesulfonamide (34)

Synthesis according to related patent literature.[S8] ^{1}H NMR (400 MHz, DMSO-d_6) δ: 12.97 (s, 1H), 9.64 (s, 1H), 9.02 (s, 2H), 8.72 (d, J = 6.0 Hz, 2H), 8.11 (s, 1H), 7.65 (dd, J = 14.5, 7.5 Hz, 1H), 7.35 (t, J = 8.8 Hz, 1H), 3.18 (dd, J = 13.5, 6.2 Hz, 2H), 2.35 – 2.22 (m, 1H), 1.32 (t, J = 7.2 Hz, 2H), 1.16 – 1.00 (m, 3H).

***N*-(2,4-difluoro-3-(5-(pyridin-4-yl)-1*H*-pyrrolo[2,3-*b*]pyridine-3-carbonyl)phenyl)propane-1-sulfonamide (38)**

Synthesis according to related patent literature[S9] ^{1}H NMR (400 MHz, DMSO-d_6) δ: 8.84 (d, J = 2.0 Hz, 1H), 8.77 (s, 1H), 8.68 (d, J = 5.8 Hz, 2H), 8.29 (s, 1H), 7.83 (d, J = 5.7 Hz, 2H), 7.63 – 7.55 (m, 1H), 7.28 (t, J = 8.4 Hz, 1H), 3.17 – 3.08 (m, 2H), 1.74 (dd, J = 15.3, 7.6 Hz, 2H), 0.96 (t, J = 7.4 Hz, 3H).

***N*-(2,6-difluoro-3-(5-(pyridin-4-yl)-1*H*-pyrrolo[2,3-*b*]pyridine-3-carbonyl)phenyl)propane-1-sulfonamide (39)**

Synthesis according to related patent literature[S9] ^{1}H NMR (400 MHz, DMSO-d_6) δ: 13.28 – 12.77 (m, 2H), 8.82 (dd, J = 9.5, 2.2 Hz, 2H), 8.68 (d, J = 5.9 Hz, 2H), 8.13 (s, 1H), 7.82 (d, J = 6.0 Hz, 2H), 7.64 (dd, J = 14.5, 7.5 Hz, 1H), 7.35 (t, J = 8.7 Hz, 1H), 3.20 – 3.09 (m, 2H), 1.81 (dd, J = 15.1, 7.6 Hz, 2H), 0.99 (t, J = 7.4 Hz, 3H).

***N*-(2,6-difluoro-3-(5-(pyridin-4-yl)-1*H*-pyrrolo[2,3-*b*]pyridine-3-carbonyl)phenyl)-1-phenylmethanesulfonamide (40)**

Synthesis according to related patent literature[S9] ^{1}H NMR (600 MHz, DMSO-d_6) δ: 13.01 (s, 1H), 9.75 (s, 1H), 8.83 (dd, J = 5.4, 2.2 Hz, 2H), 8.68 (dd, J = 4.5, 1.6 Hz, 2H), 8.14 (s, 1H), 7.82 (dd, J = 4.5, 1.6 Hz, 2H), 7.68 (dd, J = 14.4, 7.7 Hz, 1H), 7.45 (dd, J = 7.8, 1.5 Hz, 2H), 7.41 – 7.35 (m, 4H), 4.54 (s, 2H).

***N*-(2,6-difluoro-3-(5-(pyridin-4-yl)-1*H*-pyrrolo[2,3-*b*]pyridine-3-carbonyl)phenyl)ethanesulfonamide (41)**

Synthesis according to related patent literature[S9] ^{1}H NMR (600 MHz, DMSO-d_6) δ: 13.00 (s, 1H), 9.66 (s, 1H), 8.83 (d, J = 2.2 Hz, 1H), 8.80 (d, J = 2.3 Hz, 1H), 8.68 (d, J = 4.8 Hz, 2H), 8.12 (s, 1H), 7.81 (dd, J = 4.6, 1.5 Hz, 2H), 7.67 (dd, J = 14.4, 7.7 Hz, 1H), 7.36 (t, J = 8.8 Hz, 1H), 3.19 (q, J = 7.3 Hz, 2H), 1.33 (t, J = 7.3 Hz, 3H).

N-(2,6-difluoro-3-(5-(4-methyl-2-(methylthio)pyrimidin-5-yl)-1*H*-pyrrolo[2,3-*b*]pyridine-3-carbonyl)phenyl)propane-1-sulfonamide (47) The compound was synthesized in analogy to step g ^{1}H NMR (400 MHz, DMSO-d_6) δ: 12.97 (s, 1H), 9.64 (s, 1H), 8.53 (s, 1H), 8.48-8.46 (m, 1H) 8.45-8.43 (m, 1H), 8.11 (s, 1H), 7.68-7.58 (m, 1H), 7.34 (t, 1H), 3.19-3.08 (m, 1H), 2.54 (s, 2H), 2.41 (s, 3H), 1.86-1.70 (m, 2H), 0.98 (t, J = 7.4Hz, 3H) MS (ESI$^+$): 518.05 $[M+H]^+$

N-(2,6-difluoro-3-(5-(pyridazin-3-yl)-1*H*-pyrrolo[2,3-*b*]pyridine-3-carbonyl)phenyl)propane-1-sulfonamide (48) via step g ^{1}H NMR (400 MHz, DMSO-d_6) δ 13.09 (s, 1H), 9.77 (d, J = 1.3 Hz, 1H), 9.67 (s, 1H), 9.33 (d, J = 5.5 Hz, 1H), 8.93 (dd, J = 8.2, 2.2 Hz, 2H), 8.22 – 8.14 (m, 2H), 7.67 (dd, J = 14.2, 7.7 Hz, 1H), 7.36 (t, J = 8.8 Hz, 1H), 3.21

– 3.10 (m, 2H), 1.86 (dd, J = 14.9, 7.4 Hz, 2H), 1.00 (t, J = 7.4 Hz, 3H). Calculated mass: 457.10 MS(ESI^+): 458.1 for $[M+H]^+$

***Synthesis of** 1H-pyrazolo[3,4-b]pyridines*

N-(3-(4-chloro-5-(4-chlorophenyl)-1*H*-pyrrolo[2,3-*b*]pyridine-3-carbonyl)-2,4-difluorophenyl)propane-1-sulfonamide (4)

The compound was synthesized in analogy to Kloevekorn et al.[S5] using 5-bromo-4-chloro-1*H*-pyrrolo[2,3-*b*]pyridine. ^{1}H NMR (400 MHz, DMSO-d_6) δ: 8.41 (s, 1H), 8.32 (s, 1H), 7.56 (s, 4H), 7.28 (t, J = 8.7 Hz, 1H), 3.16 – 3.06 (m, 2H), 1.82 – 1.66 (m, 2H), 0.96 (t, J = 7.4 Hz, 3H).

N-(3-(5-(4-chlorophenyl)-4-cyano-1*H*-pyrrolo[2,3-*b*]pyridine-3-carbonyl)-2,4-difluorophenyl)propane-1-sulfonamide (5)

The compound was synthesized in analogy to Kloevekorn et al.[S5] using 5-Bromo-1*H*-pyrrolo[2,3-*b*]pyridine-4-carbonitrile.^{1}H NMR (400 MHz, DMSO-d_6) δ: 13.19 (s, 1H), 9.78 (s, 1H), 8.41 (s, 1H), 8.33 (s, 1H), 7.57 (s, 4H), 7.28 (t, J = 8.4 Hz, 1H), 3.16 – 3.08 (m, 2H), 1.74 (dq, J = 15.1, 7.5 Hz, 2H), 0.96 (t, J = 7.4 Hz, 3H).

N-(3-(5-(4-chlorophenyl)-6-methoxy-1*H*-pyrrolo[2,3-*b*]pyridine-3-carbonyl)-2,4-difluorophenyl)propane-1-sulfonamide (6)

Step a: to a solution of Vemurafenib (1 eq., 1 g, 2.04 mmol) in THF (20 mL) was added m-CPBA (1.2 eq., 422.61 mg, 2.45 mmol) at 0 °C. The resulting suspension was allowed to react at RT and stirred overnight. The reaction mixture was concentrated to dryness using rotary evaporator under reduced pressure. The obtained crude product was triturated with DCM to

provide a solid which was filtered and dried to obtain 5-(4-chlorophenyl)-3-(2,6-difluoro-3-(propylsulfonamido)benzoyl)-1*H*-pyrrolo[2,3-*b*]pyridine 7-oxide 2 (700 mg, 1.38 mmol, 68% yield) as a white solid.

Step b: A suspension of *N*-oxide 2 (1 eq., 500 mg, 0.988 mmol) and dimethyl sulfate (1.1 eq., 137.11 mg, 1.08 mmol) in anhydrous acetonitrile (15 mL) was stirred overnight under nitrogen atmosphere at 60 to 65 °C. After cooling to RT, a solution of NaOMe in MeOH (25 wt%, 4 mL) was added, and the turbid mixture was stirred overnight at 60 to 65 °C. After neutralization with AcOH and diluting with MeOH (5 mL), the mixture was evaporated to dryness. The obtained residue was dissolved in EtOAc (40 mL) and washed with aq. $NaHCO_3$ (5 mL × 2). The aqueous layer was extracted with EtOAc (10 mL). The combined organic layers were dried (Na_2SO_4) and evaporated to dryness. Purification by preparative HPLC yielded the target compound (30 mg, 0.0576 mmol, 5.85%) as a white solid. ^{1}H NMR (400 MHz, DMSO-d_6) δ: 12.80 (s, 1H), 9.75 (s, 1H), 8.30 (s, 1H), 7.94 (s, 1H), 7.60 (d, J = 8.5 Hz, 2H), 7.55 (dd, J = 8.9, 3.0 Hz, 1H), 7.51 (d, J = 8.5 Hz, 2H), 7.24 (t, J = 8.6 Hz, 1H), 3.94 (s, 3H), 3.15–3.04 (m, 2H), 1.73 (dq, J = 15.0, 7.5 Hz, 2H), 0.96 (t, J = 7.4 Hz, 3H).

General procedures for steps i, j, k (Scheme 2 manuscript):

Suzuki-Coupling of *N*-(2,6-difluoro-3-(1-(tetrahydro-2*H*-pyran-2-yl)-5-(4,4,5,5-tetramethyl-1,3,2-dioxaborolan-2-yl)-1*H*-pyrazolo[3,4-*b*]pyridine-3-carbonyl)phenyl)propane-1-sulfonamide (**B10**) with corresponding aryl bromides (steps j, k)

A reaction vessel was charged with the protected substance *N*-(2,6-difluoro-3-(1-(tetrahydro-2*H*-pyran-2-yl)-5-(4,4,5,5-tetramethyl-1,3,2-dioxaborolan-2-yl)-1*H*-pyrazolo[3,4-*b*]pyridine-3-carbonyl)phenyl)propane-1-sulfonamide, the corresponding aryl bromide (1.2-2 eq), Pd(dppf)Cl_2 (5 mol%) and K_2CO_3 or KF (1-3 eq). A degassed solvent mixture of 1,4-

dioxane/water (4/1 v/v) was added and the reaction was stirred at 55–65°C until complete conversion was achieved. Subsequently, the mixture was neutralized with NH_4Cl solution, extracted with EtOAc, and the product was purified by flash chromatography to isolate the protected product. For direct deprotection, the mixture was diluted with MeOH, EtOH, or i-PrOH, acidified with concentrated HCl, and stirred at 65–70°C for 16 hours. After neutralization with $NaHCO_3$ solution, the product was extracted (EtOAc or THF) and purified by flash chromatography.

Suzuki-Coupling of aryl boronic acids with *N*-(3-(5-bromo-1-(tetrahydro-2*H*-pyran-2-yl)-1*H*-pyrazolo[3,4-*b*]pyridine-3-carbonyl)-2,6-difluorophenyl)propane-1-sulfonamide (**B8**) (steps i, k) A vessel was charged with *N*-[3-[5-bromo-1-(oxan-2-yl)pyrazolo[3,4-*b*]pyridine-3-carbonyl]-2,6-difluorophenyl]propane-1-sulfonamide (72.0 mg, 0.133 mmol), boronic acid (1.1-1.5 eq), Potassium fluoride or potassium carbonate (1-3 eq) and Pd(dppf)Cl_2 · DCM (5mol%) and purged with argon. Degassed 1,4-dioxane/water (4/1 v/v) was added and the mixture stirred at 80 °C until competition of the reaction. After cooling, the mixture was diluted with methanol, acidified with conc. HCl and stirred at 60 °C for 2h. Water and EtOAc were added and the organic layer was separated, washed with brine, dried over sodium sulfate and evaporated. The product was purified by flash chromatography.

N-(3-(5-(4-chlorophenyl)-1*H*-pyrazolo[3,4-*b*]pyridine-3-carbonyl)-2,6-difluorophenyl)propane-1-sulfonamide (21)

The compound was synthesized in analogy to Pfaffenrot et al.[S6] ^{1}H NMR (200 MHz, DMSO-d_6) δ: 14.81 (s, 1H), 9.66 (s, 1H), 9.01 (d, J = 2.1 Hz, 1H), 8.77 (d, J = 2.1 Hz, 1H), 7.98 – 7.78 (m, 3H), 7.60 (d, J = 8.4 Hz, 2H), 7.40 (t, J = 8.7 Hz, 1H), 3.22 – 3.09 (m, 2H), 1.92 – 1.70 (m, 2H), 1.00 (t, J = 7.5 Hz, 3H).

***N*-(3-(5-(4-chlorophenyl)-1*H*-pyrazolo[3,4-*b*]pyridine-3-carbonyl)-2,4,6-trifluorophenyl)propane-1-sulfonamide (25)**

The compound was synthesized in analogy to Pfaffenrot et al.[S6] ^{1}H NMR (400 MHz, DMSO-d_6) δ: 14.97 (s, 1H), 9.66 (s, 1H), 9.04 (d, J = 2.2 Hz, 1H), 8.77 (d, J = 2.2 Hz, 1H), 8.05 – 7.69 (m, 2H), 7.71 – 7.44 (m, 3H), 3.21 – 3.02 (m, 2H), 1.92 – 1.62 (m, 2H), 0.99 (t, J = 7.4 Hz, 3H).

N-(3-(5-(2-chloro-4-methoxyphenyl)-1H-pyrazolo[3,4-b]pyridine-3-carbonyl)-2,6-difluorophenyl)propane-1-sulfonamide (27)

Synthesis according to related patent literature.[S14]

^{1}H NMR (400 MHz, DMSO-d_6) δ: 14.82 (s, 1H), 9.66 (s, 1H), 8.72 (d, J = 1.8 Hz, 1H), 8.57 (d, J = 1.8 Hz, 1H), 7.86 (dd, J = 15.0, 7.4 Hz, 1H), 7.53 (d, J = 8.6 Hz, 1H), 7.39 (t, J = 8.7 Hz, 1H), 7.25 (d, J = 2.3 Hz, 1H), 7.10 (dd, J = 8.6, 2.4 Hz, 1H), 3.86 (s, 3H), 3.15 (t, J = 7.6 Hz, 2H), 1.81 (dq, J = 15.0, 7.5 Hz, 2H), 1.00 (t, J = 7.4 Hz, 3H).

***N*-(2,6-difluoro-3-(5-(4-fluoro-2-methylphenyl)-1*H*-pyrazolo[3,4-*b*]pyridine-3-carbonyl)phenyl)propane-1-sulfonamide (29)** The compound was synthesized in analogy *to* step i. ^{1}H NMR (400 MHz, DMSO-d_6) δ: 14.80 (s, 1H), 9.65 (s, 1H), 8.67 (d, J = 1.9 Hz, 1H), 8.47 (d, J = 2.0 Hz, 1H), 7.85 (dd, J = 14.8, 7.5 Hz, 1H), 7.43 – 7.34 (m, 1H), 7.25 (dd, J = 10.1, 2.4 Hz, 1H), 7.17 (td, J = 8.5, 2.6 Hz, 1H), 3.20 – 3.08 (m, 1H), 2.27 (s, 1H), 1.88 – 1.73 (m, 1H), 1.00 (t, J = 7.5 Hz, 1H); ^{13}C NMR (101 MHz, DMSO-d_6) δ 185.2, 163.0, 160.6, 151.7, 150.7, 141.6, 138.5, 138.4, 134.1, 134.1, 132.2, 132.1, 132.0, 130.2, 123.5, 117.0, 116.8, 114.2, 113.7, 113.0, 112.8, 112.0, 112.0, 111.8, 111.8, 54.9, 20.1, 16.8, 12.6; m/z: 487.1 $[M-H]^-$.

N-(3-(5-(2-cyclopropylpyrimidin-5-yl)-1H-pyrazolo[3,4-b]pyridine-3-carbonyl)-2,6-difluorophenyl)-1-phenylmethanesulfonamide (31)

Synthesis according to related patent literature.[S14] ^{1}H NMR (400 MHz, DMSO-d_6) δ: 14.88 (s, 1H), 9.76 (s, 1H), 9.10 (s, 1H), 9.05 (d, J = 2.1 Hz, 1H), 8.88 (d, J = 2.1 Hz, 1H), 7.92 – 7.83 (m, 1H), 7.48 – 7.35 (m, 5H), 4.52 (s, 2H), 2.30 (m, 1H), 1.16 – 1.05 (m, 3H).

***N*-(2,6-difluoro-3-(5-(pyridin-4-yl)-1*H*-pyrazolo[3,4-*b*]pyridine-3-carbonyl)phenyl) methanesulfonamide (43)**

Synthesis according to related patent literature[S10] ^{1}H NMR (200 MHz, DMSO-d_6) δ: 15.09 (s, 1H), 9.77 (s, 1H), 9.28 (d, J = 2.3 Hz, 1H), 9.13 (d, J = 2.3 Hz, 1H), 8.99 (d, J = 6.7 Hz, 2H), 8.52 (d, J = 6.7 Hz, 2H), 7.90 (dd, J = 15.0, 7.6 Hz, 1H), 7.42 (td, J = 8.9, 1.2 Hz, 1H), 3.12 (s, 3H).

***N*-(2,6-difluoro-3-(5-(pyridin-4-yl)-1*H*-pyrazolo[3,4-*b*]pyridine-3-carbonyl)phenyl) ethanesulfonamide (44)**

Synthesis according to related patent literature[S10] ^{1}H NMR (400 MHz, DMSO-d_6) δ: 14.89 (s, 1H), 9.66 (s, 1H), 9.12 (d, J = 2.1 Hz, 1H), 8.91 (d, J = 2.3 Hz, 1H), 8.72 (d, J = 5.9 Hz, 2H), 7.91 – 7.83 (m, 3H), 7.40 (t, J = 8.8 Hz, 1H), 3.18 (q, J = 7.3 Hz, 2H), 1.33 (t, J = 7.3 Hz, 3H).

Synthesis of Carbolines

***N*-(2,4-difluoro-3-(3-(pyridin-3-yl)-9*H*-pyrido[2,3-*b*]indole-6-carbonyl)phenyl)propane-1-sulfonamide (18)**

Synthesis according to related patent literature[S7]

^{1}H NMR (400 MHz, DMSO-d_6) δ: 12.58 (s, 1H), 9.83 (s, 1H), 9.13 (d, *J* = 1.4 Hz, 1H), 9.04 (d, *J* = 1.6 Hz, 1H), 8.88 (d, *J* = 2.2 Hz, 1H), 8.82 (s, 1H), 8.60 (d, *J* = 3.7 Hz, 1H), 8.30 – 8.16 (m, 1H), 8.04 (dd, *J* = 8.6, 1.3 Hz, 1H), 7.73 – 7.61 (m, 2H), 7.53 (dd, *J* = 7.8, 4.8 Hz, 1H), 7.35 (t, *J* = 8.7 Hz, 1H), 3.22 – 3.08 (m, 2H), 1.85 – 1.60 (m, 2H), 0.92 (t, *J* = 7.4 Hz, 3H).

N-(2,4-difluoro-3-(3-(pyridazin-4-yl)-9*H*-pyrido[2,3-*b*]indole-6-carbonyl)phenyl)propane-1-sulfonamide (19)

The compound was synthesized in analogy to Juchum et al.[S7] ^{1}H NMR (200 MHz, DMSO-d_6) δ: 12.75 (s, 1H), 10.03 – 9.64 (m, 2H), 9.43 – 9.23 (m, 2H), 9.10 (s, 1H), 8.82 (s, 1H), 8.25 – 8.01 (m, 2H), 7.80 – 7.58 (m, 2H), 7.36 (t, J = 8.1 Hz, 1H), 3.22 – 3.08 (m, 2H), 1.87 – 1.62 (m, 2H), 0.92 (t, J = 7.4 Hz, 3H).

V: NMR and HPLC Data of lead compounds

Compound **16**

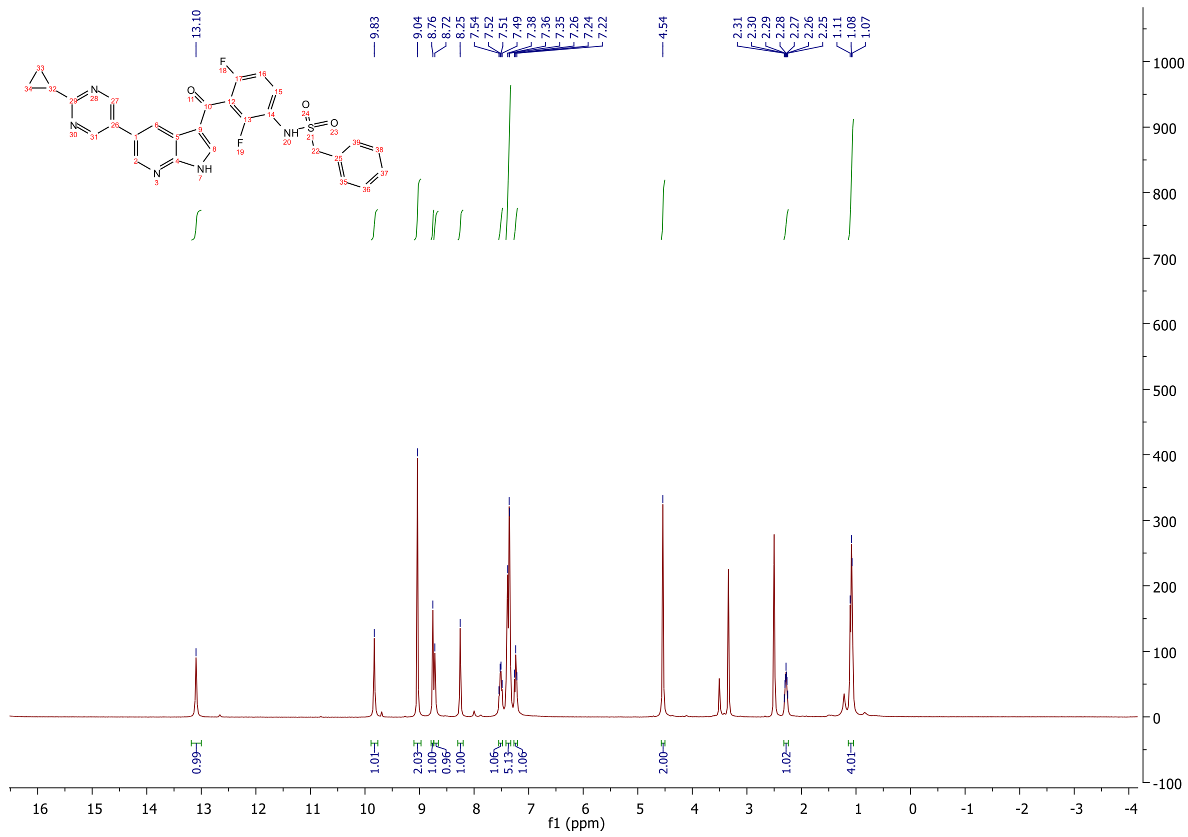

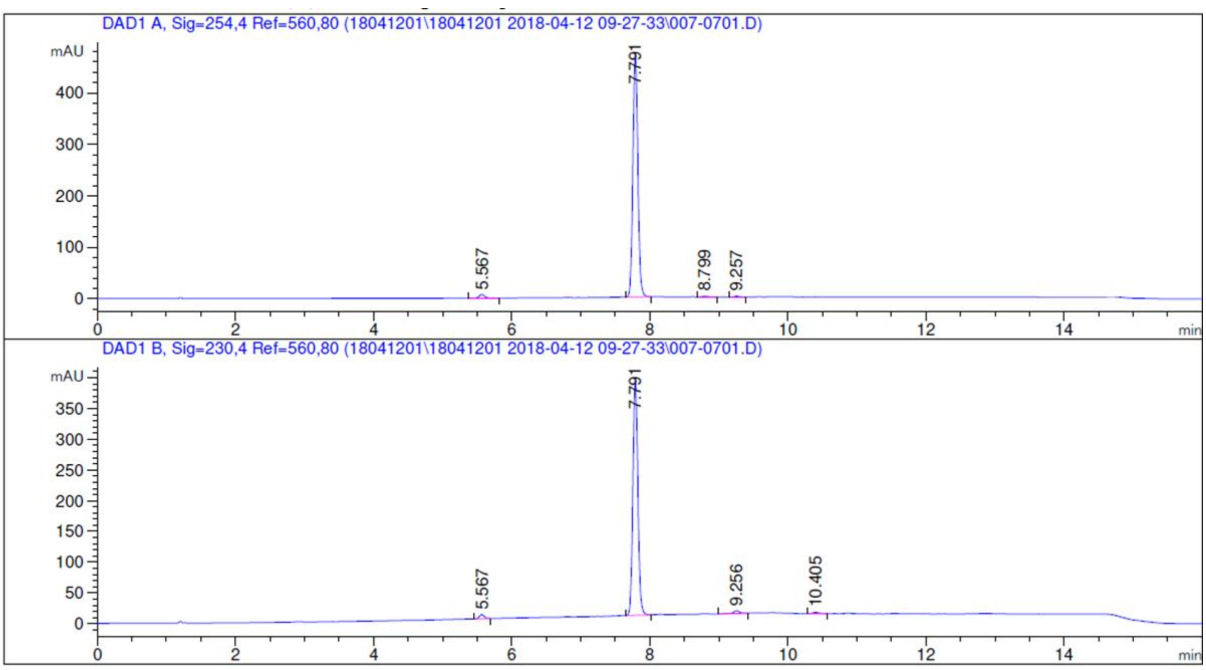

Signal 1: DAD1 A, Sig=254,4 Ref=560,80

Peak #	RetTime [min]	Type	Width [min]	Area [mAU*s]	Height [mAU]	Area %
1	5.567	BB	0.0774	35.24822	6.91807	1.4638
2	7.791	BB	0.0782	2358.99878	472.89938	97.9637
3	8.799	BB	0.0874	7.24125	1.29189	0.3007
4	9.257	BB	0.0792	6.54417	1.28928	0.2718
Totals :				2408.03242	482.39862	

Signal 2: DAD1 B, Sig=230,4 Ref=560,80

Peak #	RetTime [min]	Type	Width [min]	Area [mAU*s]	Height [mAU]	Area %
1	5.567	BB	0.0753	32.43866	6.60752	1.6297
2	7.791	BB	0.0782	1919.02783	384.66284	96.4137
3	9.256	BB	0.0892	25.79954	4.34715	1.2962
4	10.405	BB	0.0929	13.14306	2.16363	0.6603
Totals :				1990.40910	397.78114	

Compound **36**

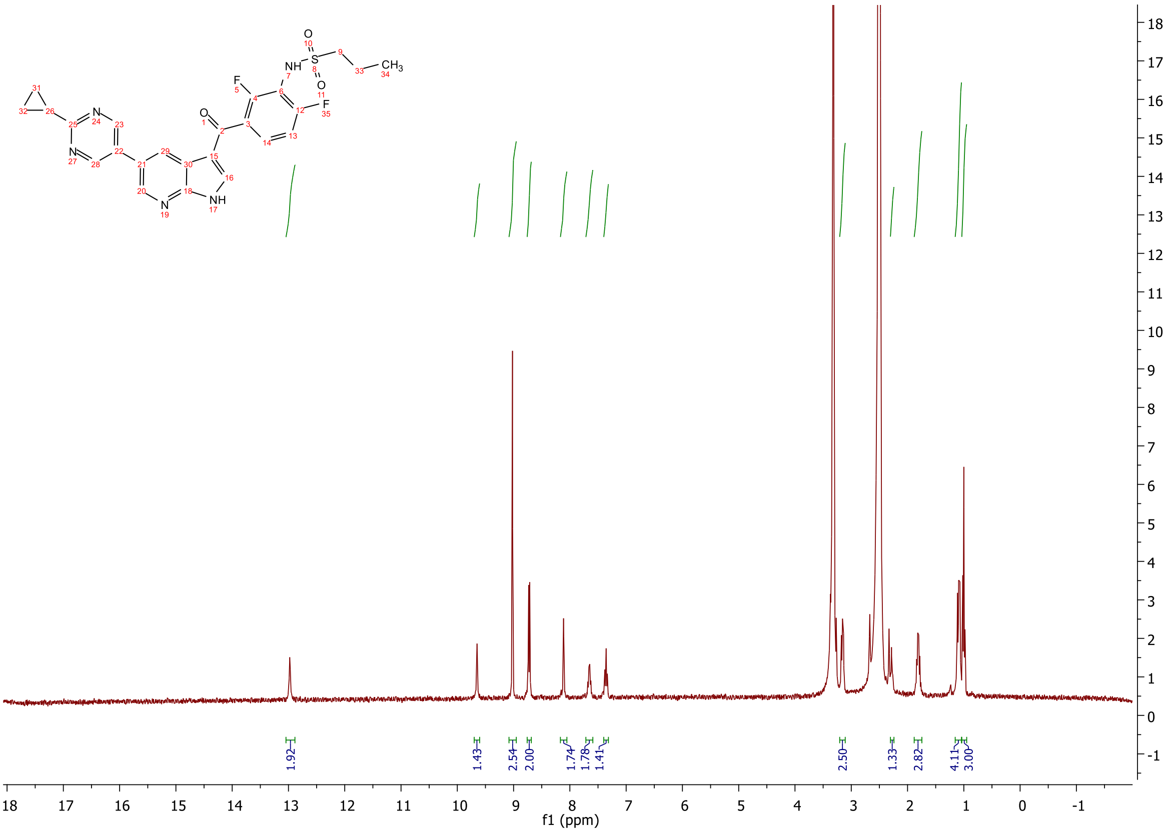

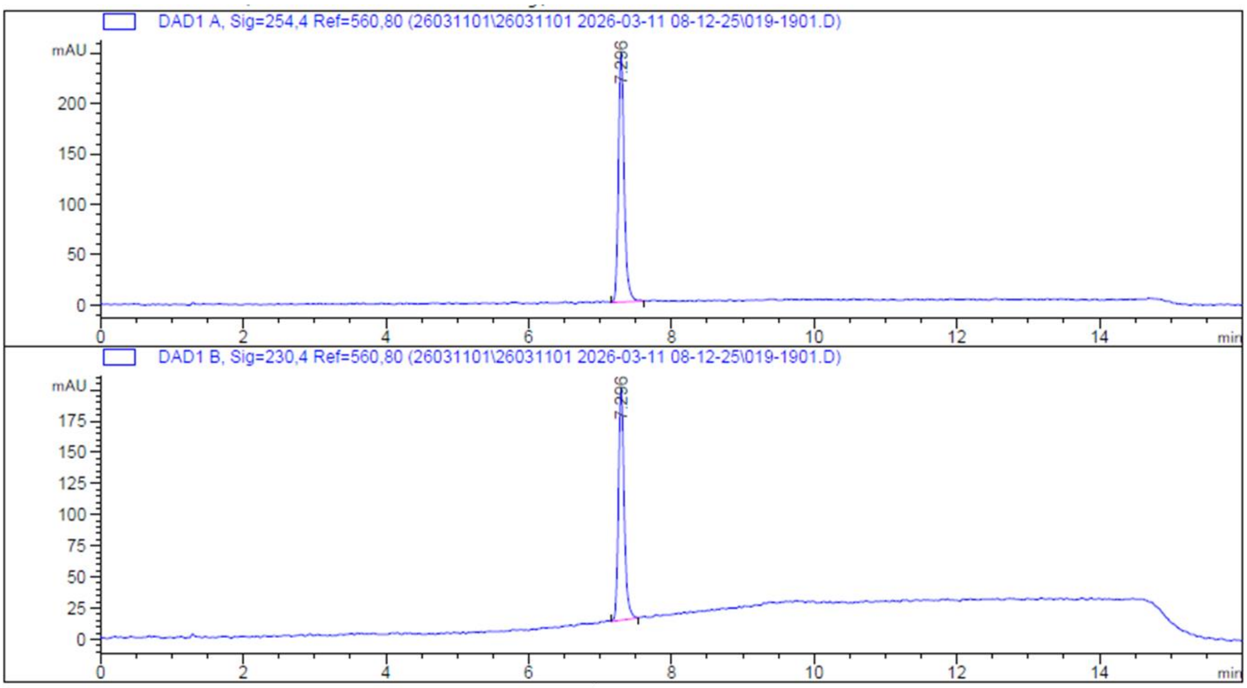

Signal 1: DAD1 A, Sig=254,4 Ref=560,80

Peak #	RetTime [min]	Type	Width [min]	Area [mAU*s]	Height [mAU]	Area %
1	7.296	BB	0.0854	1390.33032	248.12433	100.0000
Totals :				1390.33032	248.12433	

Peak #	RetTime [min]	Type	Width [min]	Area [mAU*s]	Height [mAU]	Area %
1	7.296	BB	0.0848	1034.94592	186.29616	100.0000
Totals :				1034.94592	186.29616	

Compound **37**

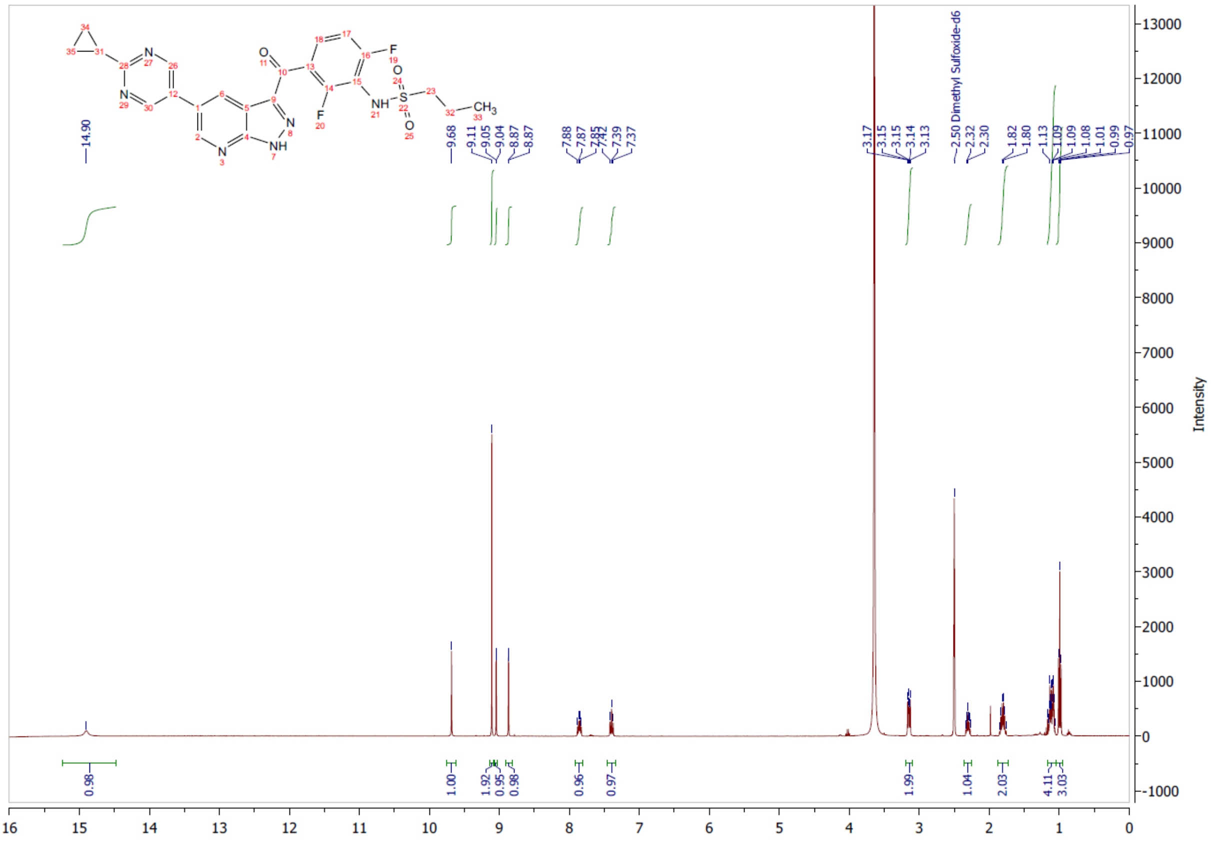

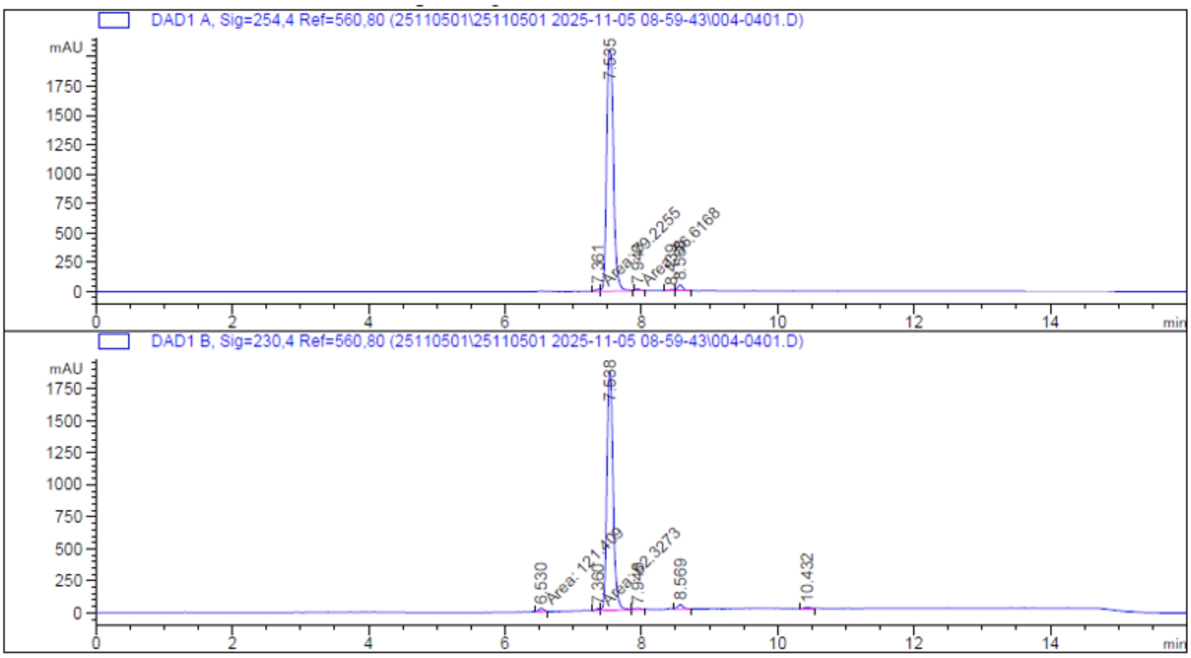

Signal 1: DAD1 A, Sig=254,4 Ref=560,80

Peak #	RetTime [min]	Type	Width [min]	Area [mAU*s]	Height [mAU]	Area %
1	7.361	MM T	0.0800	79.22552	16.51021	0.5351
2	7.535	VV	0.1115	1.43407e4	2051.62866	96.8604
3	7.946	MM T	0.0769	56.61684	12.26940	0.3824
4	8.439	BV	0.0748	48.96695	10.06203	0.3307
5	8.568	VB	0.0852	280.02087	50.09413	1.8913

Totals : 1.48055e4 2140.56443

Signal 2: DAD1 B, Sig=230,4 Ref=560,80

Peak #	RetTime [min]	Type	Width [min]	Area [mAU*s]	Height [mAU]	Area %
1	6.530	MM T	0.0811	121.40860	24.94732	0.9855
2	7.360	MM T	0.0818	62.32732	12.70544	0.5059
3	7.538	VV	0.0998	1.18081e4	1866.73694	95.8539
4	7.946	VV	0.0887	69.86449	11.20442	0.5671
5	8.569	VB	0.0871	201.64027	35.06157	1.6368
6	10.432	BV	0.0909	55.50807	9.40507	0.4506

Totals : 1.23189e4 1960.06075

Compound **42**

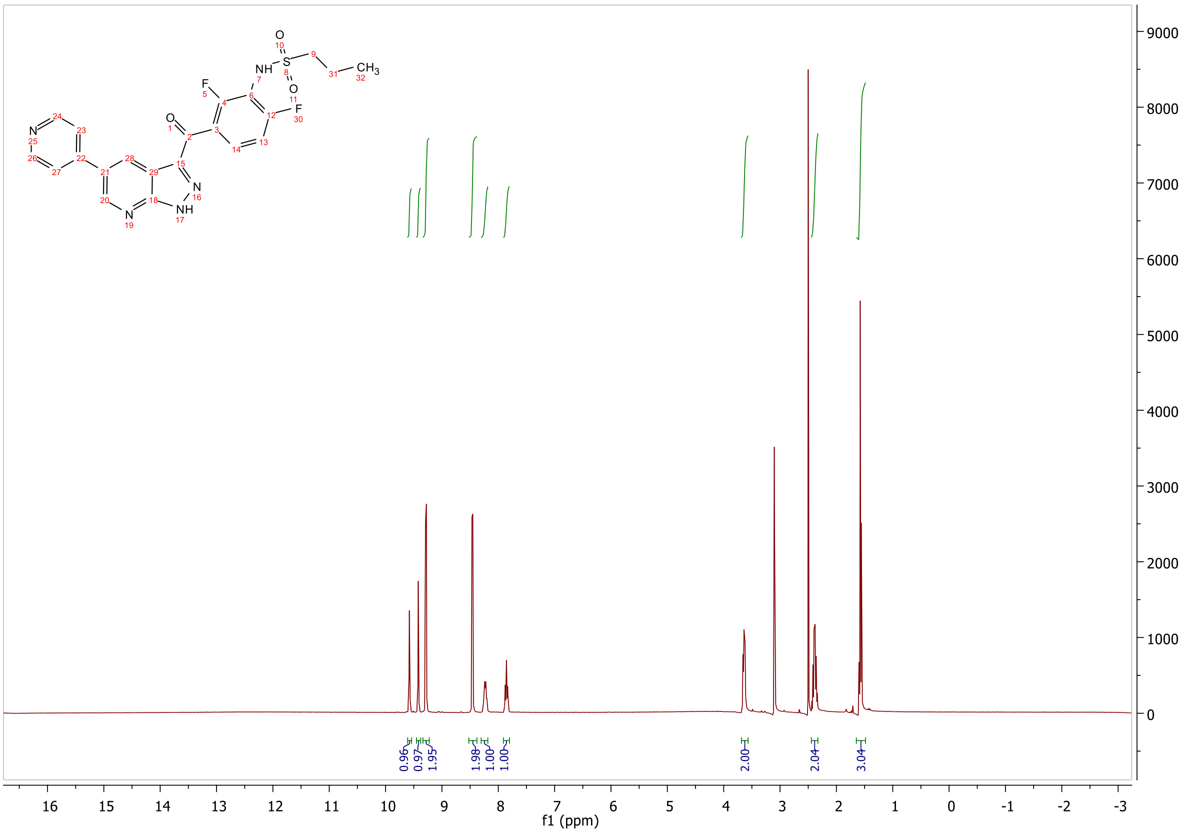

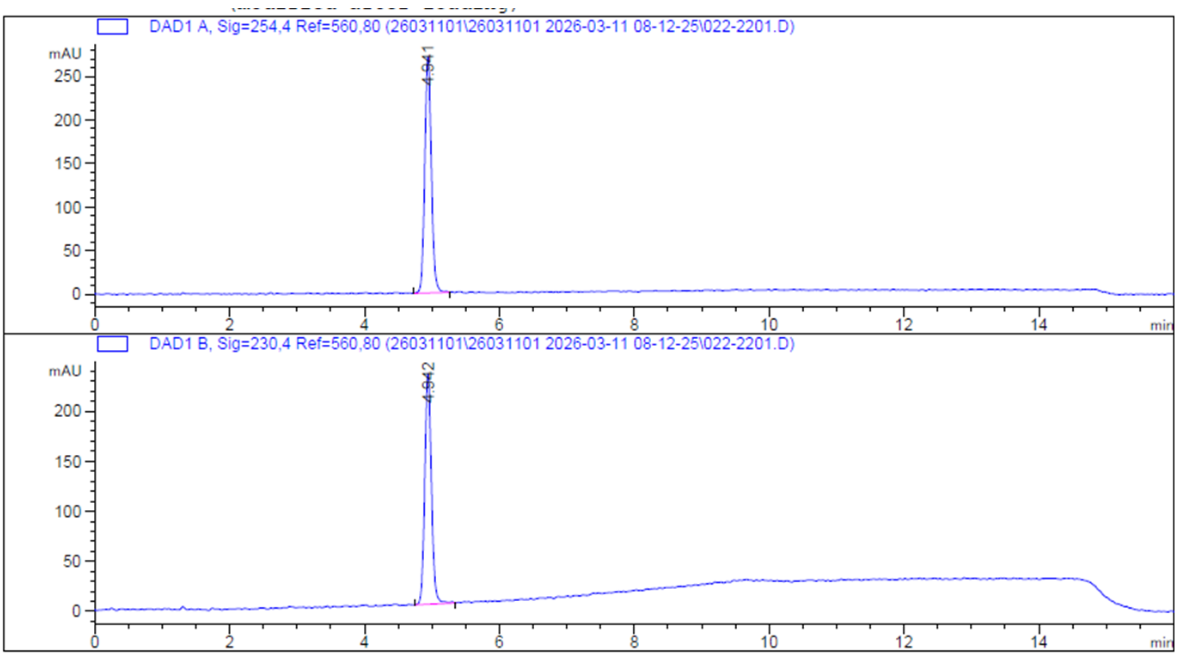

Signal 1: DAD1 A, Sig=254,4 Ref=560,80

Peak #	RetTime [min]	Type	Width [min]	Area [mAU*s]	Height [mAU]	Area %
1	4.941	BB	0.1052	1846.54468	271.91867	100.0000
Totals :				1846.54468	271.91867	

Signal 2: DAD1 B, Sig=230,4 Ref=560,80

Peak #	RetTime [min]	Type	Width [min]	Area [mAU*s]	Height [mAU]	Area %
1	4.942	BB	0.1061	1586.05200	230.87469	100.0000
Totals :				1586.05200	230.87469	

Compound **45**

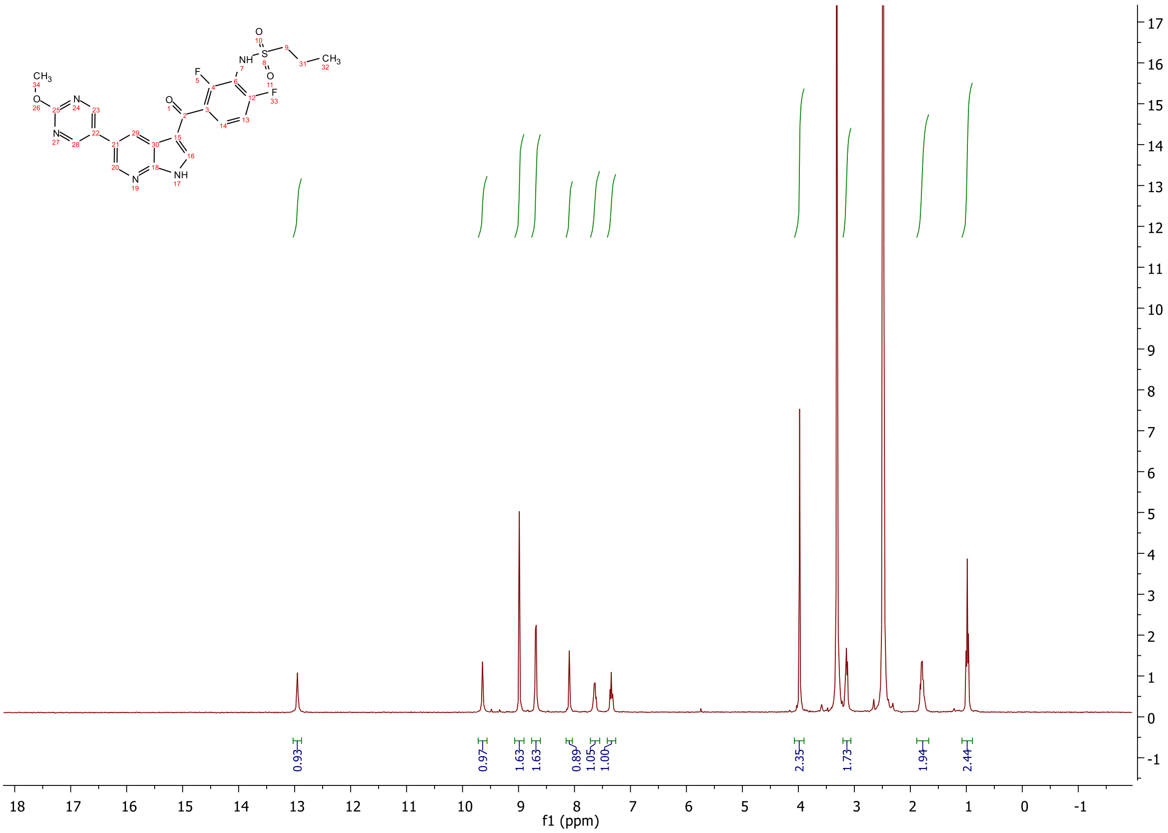

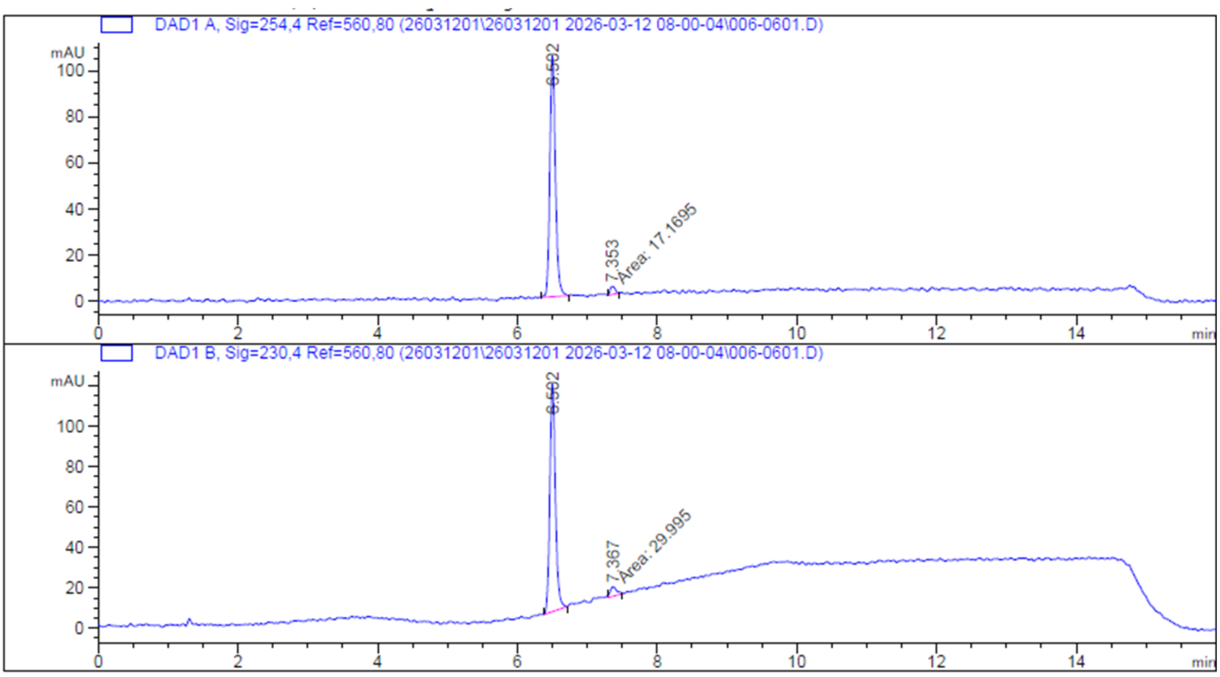

Signal 1: DAD1 A, Sig=254,4 Ref=560,80

Peak #	RetTime [min]	Type	Width [min]	Area [mAU*s]	Height [mAU]	Area %
1	6.502	BB	0.0845	579.70099	104.92824	97.1234
2	7.353	MM T	0.0816	17.16946	3.50655	2.8766
Totals :				596.87045	108.43479	

Signal 2: DAD1 B, Sig=230,4 Ref=560,80

Peak #	RetTime [min]	Type	Width [min]	Area [mAU*s]	Height [mAU]	Area %
1	6.502	BB	0.0844	622.58289	112.85825	95.4036
2	7.367	MM T	0.1051	29.99500	4.75717	4.5964
Totals :				652.57788	117.61542	

Compound **46**

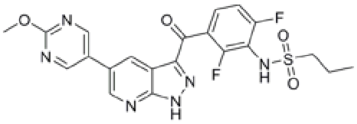

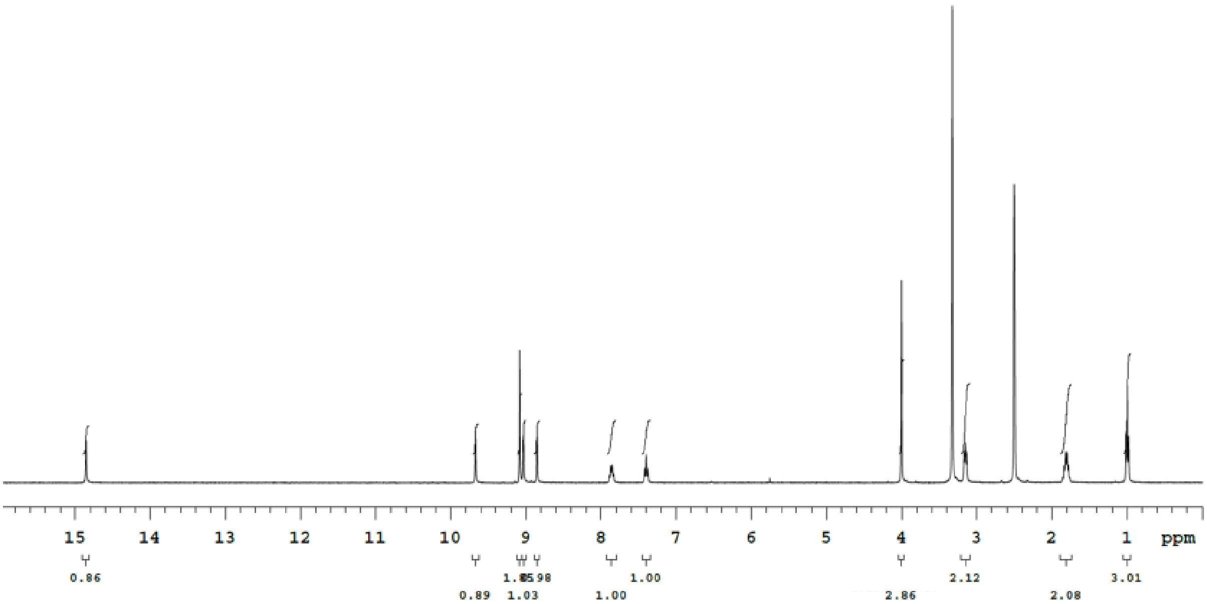

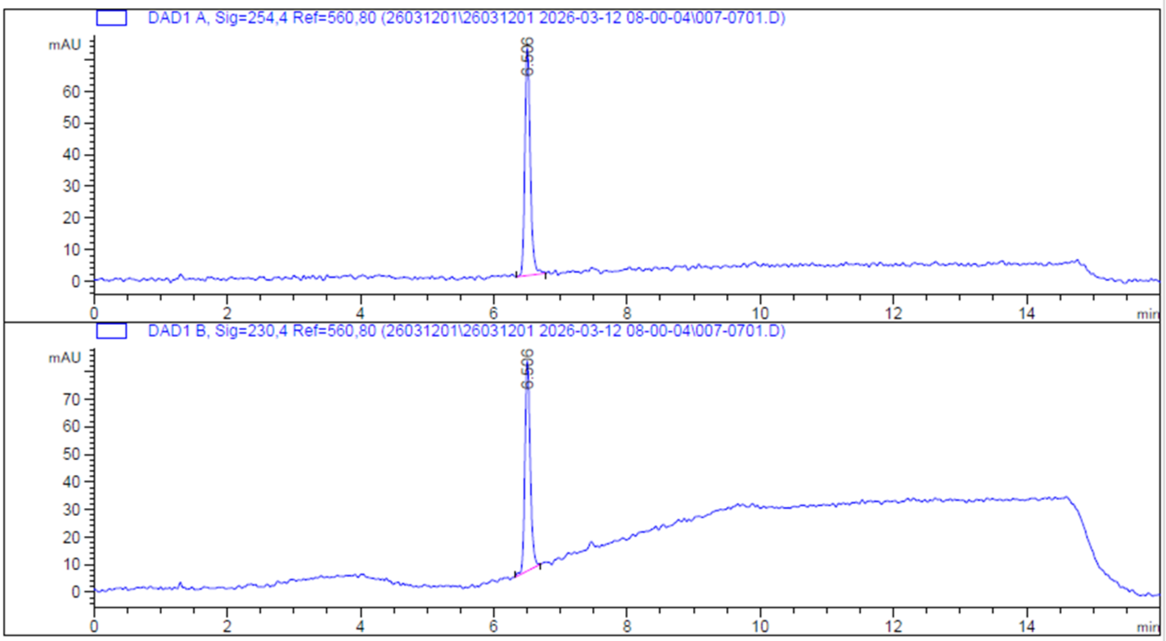

Signal 1: DAD1 A, Sig=254,4 Ref=560,80

Peak #	RetTime [min]	Type	Width [min]	Area [mAU*s]	Height [mAU]	Area %
1	6.506	BB	0.0880	407.52960	72.08212	100.0000
Totals :				407.52960	72.08212	

Signal 2: DAD1 B, Sig=230,4 Ref=560,80

Peak #	RetTime [min]	Type	Width [min]	Area [mAU*s]	Height [mAU]	Area %
1	6.506	BB	0.0849	424.63980	76.30429	100.0000
Totals :				424.63980	76.30429	

VI: Supplementary figures on pharmacological data

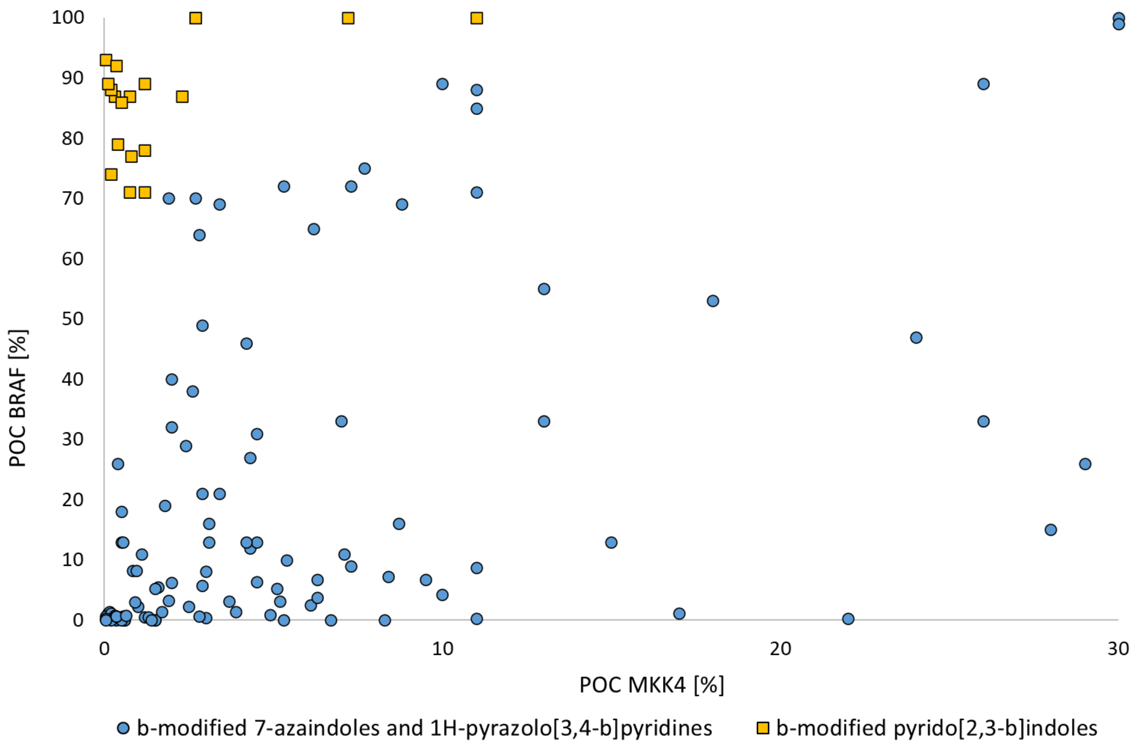

Figure S 1: Affinity of 7-azaindoles, pyrazolo[3,4-b]pyridines and pyrido[2,3-b]indoles to MKK4 (x-axis) and BRaf (y-axis), determined at a concentration of 100 nM and quantified by calculation of PoC (percent of control; 100 = no binding, 0=quantitative occupation); ligands were modified in position "b".

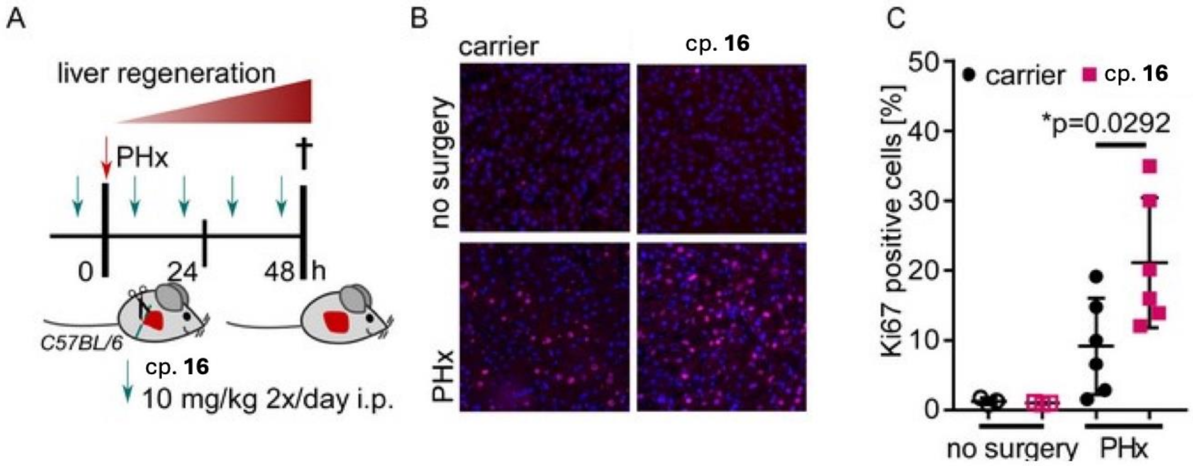

Figure S 2: Effect of cp. ***16*** *in the partial hepatectomy model in C57BL/6 wt mice.*

A: Study design – **16** was administered at a dose of 10 mg/kg i.p. every 12 h, the fist dose given 1 h prior the partial hepatectomy. After 48 h, mice were sacrificed and proliferation was determined with quantification of Ki67 in liver tissue sections. **B:** Representative images of mice that underwent partial hepatectomy, with and without **16** (lower panel) and mice treated with inhibitor or carrier only without surgery (upper panel). **C:** Quantification of KI67 positive cells in liver tissue. (p=0.0292 student's t-test; n=3 (no surgery) and n=6 (PHx) per group).

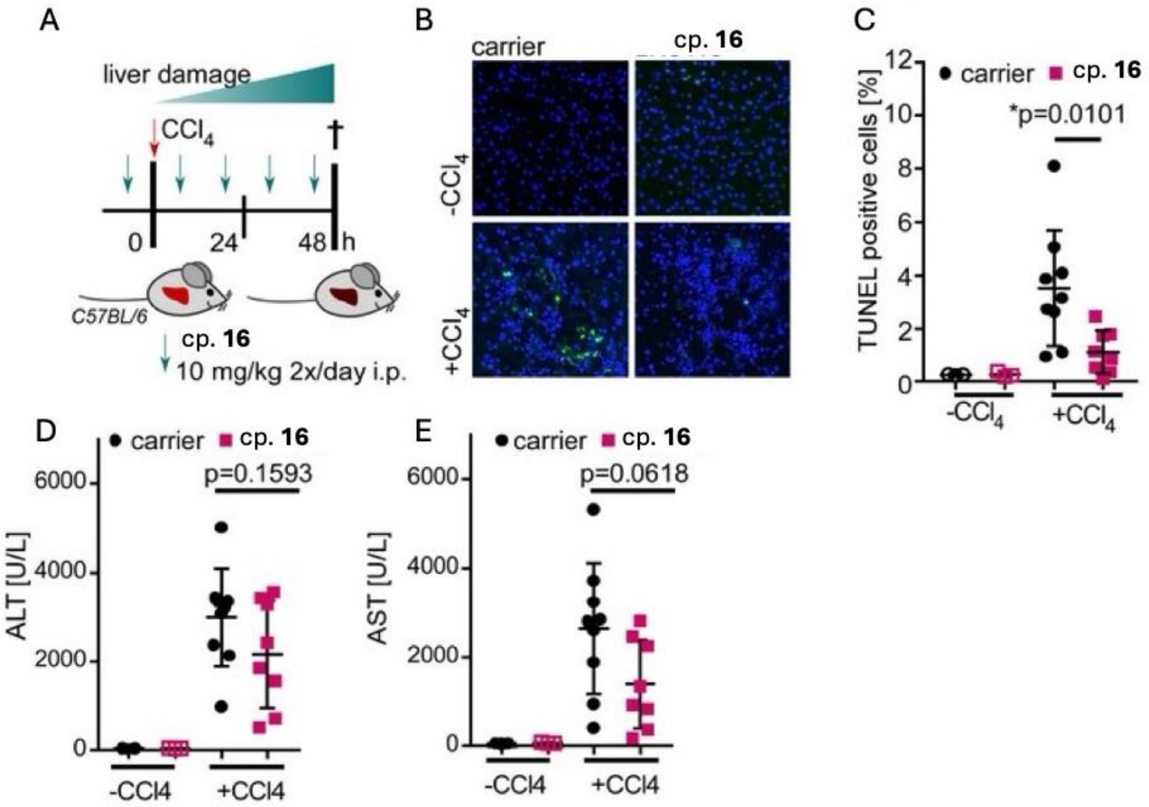

Figure S 3: Effect of cp. ***16*** *in the acute liver injury model in C57BL/6 wt mice.*

A: *Study design –* ***16*** *was administered at a dose of 10 mg/kg i.p. every 12 h, the fist dose given 1 h prior to induction of liver injury by i.p. injection of CCl_4. After 48 h, mice were sacrificed and cell death was determined with quantification of TUNEL (green) in liver tissue sections. Cell nuclei are counterstained with DAPI. Scale bar represents 100 µm.*

B: *Representative images of mice that were treated with 1x CCl_4 with and without* ***16*** *(lower panel) and mice treated with inhibitor or carrier only (upper panel).*

C: *Quantification of TUNEL-positive cells (p=0.0101 student's t-test;n=3 (-CCl_4) and n=8 (+CCl_4) per group)*

D: *Quantification (mean ± s.d., n: see C) of ALT activity in plasma, determined 48 h after CCl_4 treatment in plasma (retrobulbar blood sampling); p=0.1593 (Student's t-test).*

E: *Quantification (mean ± s.d., n: see C) of AST activity in plasma, determined 48 h after CCl_4 treatment in plasma (retrobulbar blood sampling); p=0.0618 (Student's t-test).*

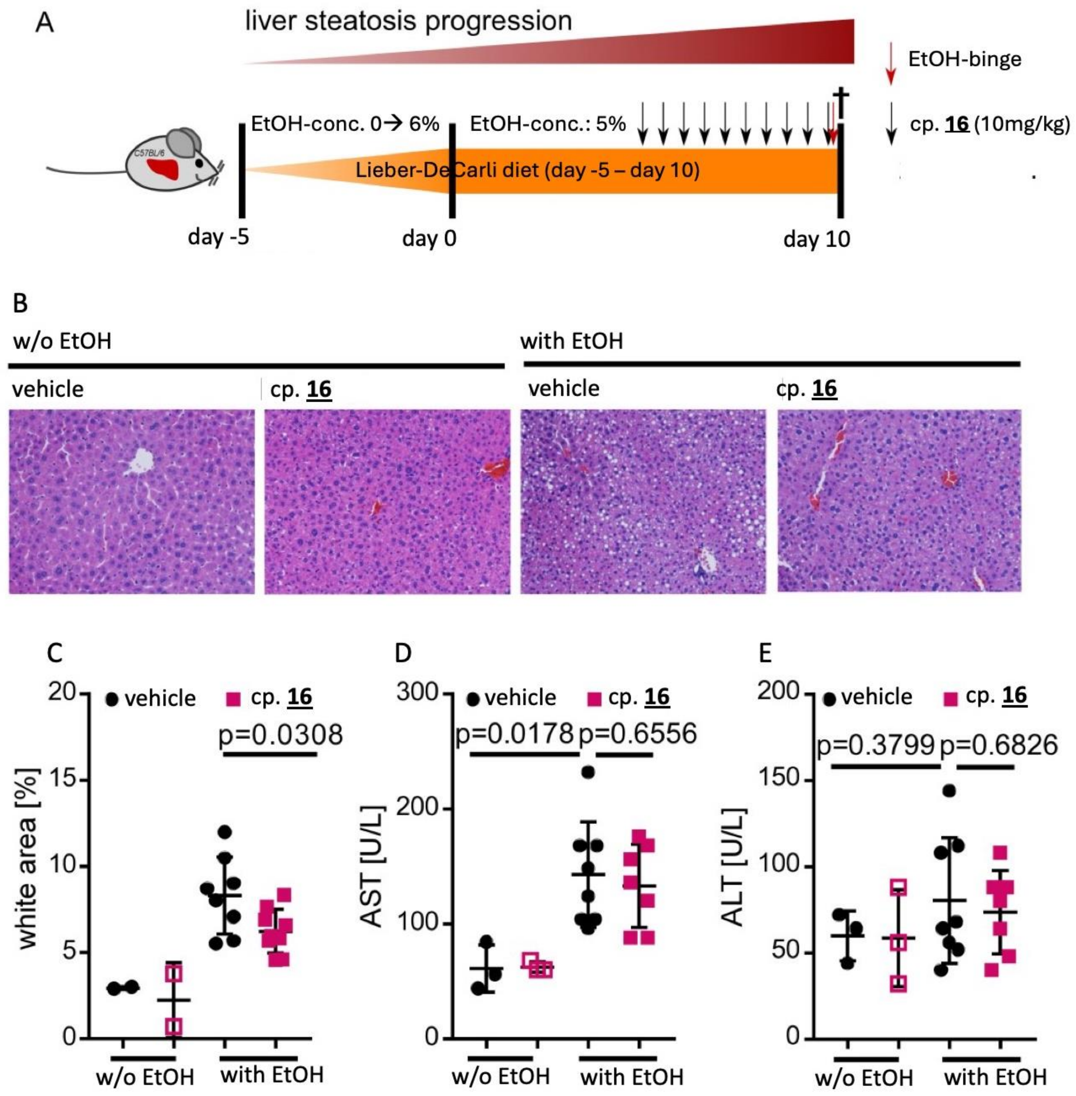

Figure S 4: Effect of cp. ***16*** *in experimental alcoholic steatohepatitis in C57BL/6 wt mice.*

A: *Study design. Mice were fed with Lieber-DeCarli Diet substituted with increasing amount of alcohol until a plateau of 6% alcohol in the food was achieved (= day 0). Between day 5 and day 10, day 5, cp.* ***16*** *(10 mg/kg) or vehicle was administered twice daily. On day 10, 2h after final administration of cp.* ***16*** *or vehicle, animals were dosed with 5g/kg and sacrificed 9 h later.*
B: *H&E staining of liver tissue slices. White vesicles in the H&E staining indicate washed out fat which can be quantified. Scale bar represents 200 µm.*
C: *Quantification of images displayed in B. Quantification of white tissue is shown. (n=2-3 per group (w/o EtOH), n=8 per group (with EtOH); p=0.0308 student's t-test).*
D: *AST concentration is determined 9 h after the binge in plasma from retro bulbar blood sampling. Mean and SD are indicated, n: see C, p=0.6556 (Student's t-test).*
E: *ALT concentration is determined 9 h after the binge in plasma from retro bulbar blood sampling. Mean and SEM are indicated. n: see C, p=0.6826 (Student's t-test).*

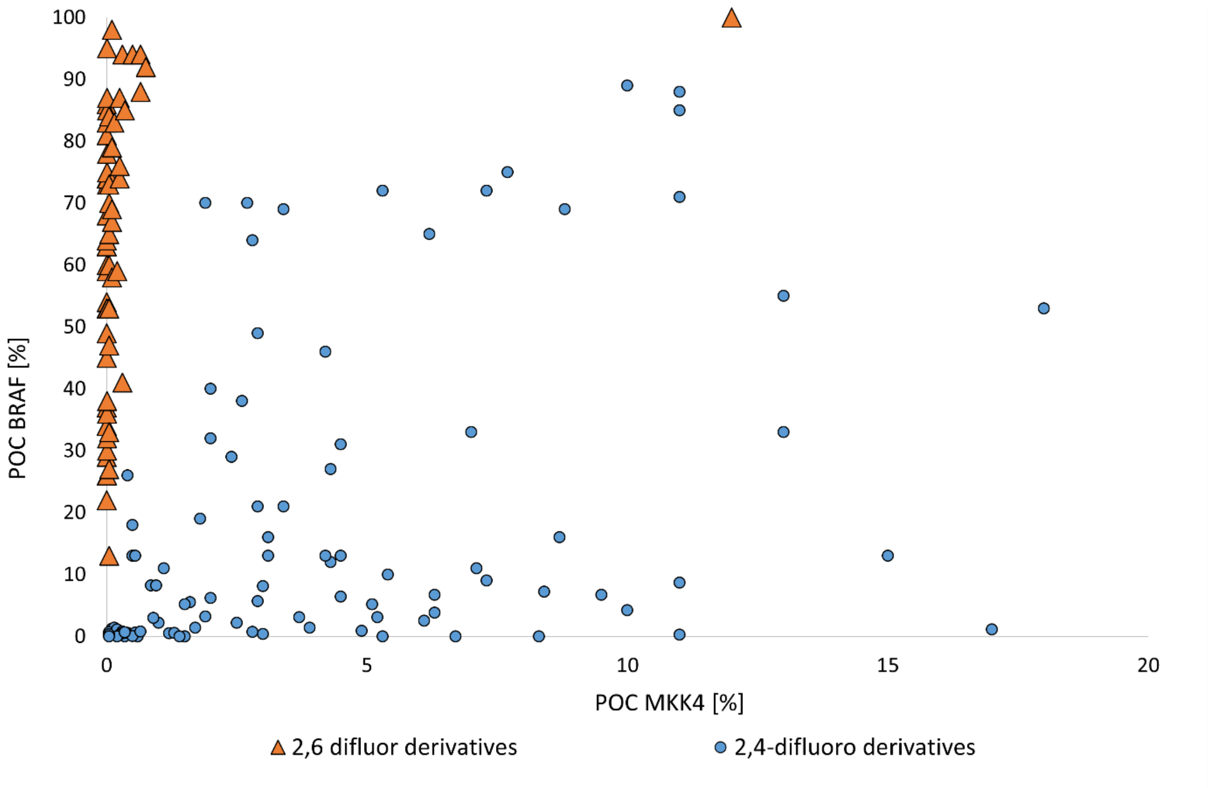

Figure S 5: PoC (percent of control at 100 nM) of 2,4-difluoro b-modified scaffolds 1 and 2 BRaf vs MKK4 compared to 2,6-difluoro b-modified 7-azaindole and pyrazolopyridine scaffolds.

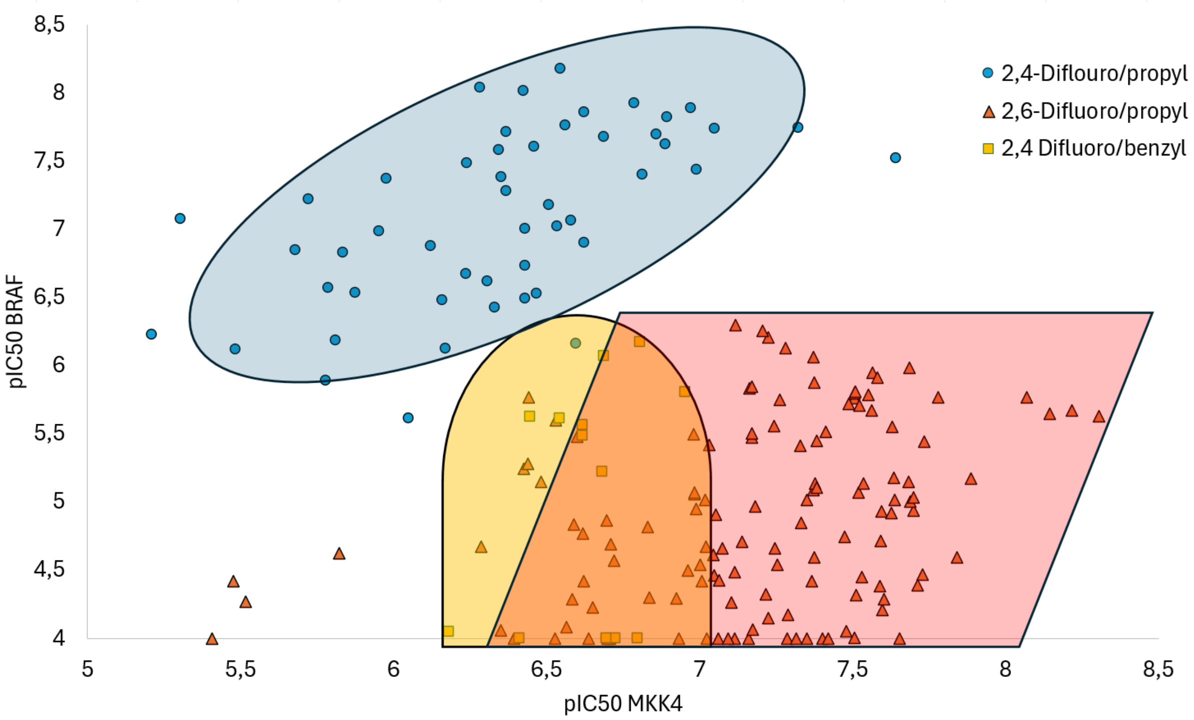

Figure S 6: pIC_{50} BRaf vs MKK4 of 2,4-difluoro derivatives, 2,6-difluoro derivatives and the corresponding benzyl derivatives.

Customer Id	CYP 1A2			CYP 2B6		
	no pre-inc.	30 min. pre-inc. w/o NADPH	30 min. pre-inc. + NADPH	0 minute Pre-incubation	30 min. pre-inc. w/o NADPH	30 min. pre-inc. + NADPH
	IC_{50} (µM)	IC_{50} (µM)	IC_{50} (µM)	IC_{50} (µM)	IC_{50} (µM)	IC_{50} (µM)
**36**	>12.5	>12.5	>12.5	>12.5	>12.5	>12.5
**42**	>12.5	>12.5	>12.5	7.20	9.14	>12.5
**37**	>12.5	>12.5	>12.5	>12.5	>12.5	>12.5
**47**	>12.5	>12.5	>12.5	>12.5	>12.5	>12.5
**46**	>12.5	>12.5	>12.5	>12.5	>12.5	>12.5

Customer Id	CYP 2C8			CYP 2C9			CYP 2C19		
	no pre-inc.	30 min. pre-inc. w/o NADPH	30 min. pre-inc. + NADPH	0 minute Pre-incubation	30 min. pre-inc. w/o NADPH	30 min. pre-inc. + NADPH	no pre-inc.	30 min. pre-inc. w/o NADPH	30 min. pre-inc. + NADPH
	IC_{50} (µM)	IC_{50} (µM)	IC_{50} (µM)	IC_{50} (µM)	IC_{50} (µM)	IC_{50} (µM)	IC_{50} (µM)	IC_{50} (µM)	IC_{50} (µM)
**36**	6.50	7.24	6.02	3.87	4.28	5.91	>12.5	>12.5	>12.5
**42**	7.38	8.81	9.13	4.34	4.93	6.43	4.74	5.03	5.46
**37**	>12.5	>12.5	>12.5	>12.5	10.2	>12.5	>12.5	>12.5	>12.5
**47**	8.27	10.5	10.7	2.42	2.25	5.25	>12.5	>12.5	>12.5
**46**	>12.5	>12.5	>12.5	>12.5	>12.5	>12.5	>12.5	>12.5	>12.5

Customer Id	CYP 3A4(a)			CYP 3A4(b)			CYP 2D6		
	no pre-inc.	30 min. pre-inc. w/o NADPH	30 min. pre-inc. + NADPH	no pre-inc.	30 min. pre-inc. w/o NADPH	30 min. pre-inc. + NADPH	no pre-inc.	30 min. pre-inc. w/o NADPH	30 min. pre-inc. + NADPH
	IC_{50} (µM)	IC_{50} (µM)	IC_{50} (µM)	IC_{50} (µM)	IC_{50} (µM)	IC_{50} (µM)	IC_{50} (µM)	IC_{50} (µM)	IC_{50} (µM)
**36**	>12.5	>12.5	>12.5	>12.5	>12.5	>12.5	>12.5	>12.5	>12.5
**42**	>12.5	>12.5	>12.5	4.20	4.65	5.72	0.757	1.08	1.78
**37**	>12.5	>12.5	>12.5	>12.5	>12.5	>12.5	>12.5	>12.5	>12.5
**47**	>12.5	>12.5	>12.5	>12.5	>12.5	>12.5	>12.5	>12.5	>12.5
**46**	>12.5	>12.5	>12.5	>12.5	>12.5	>12.5	>12.5	>12.5	>12.5

(a) Substrate = testosterone; (b) substrate = midazolam

Table S 1: Inhibition of cytochrome P450 (CYP) isoforms in the presence of shortlisted MKK4 inhibitors.

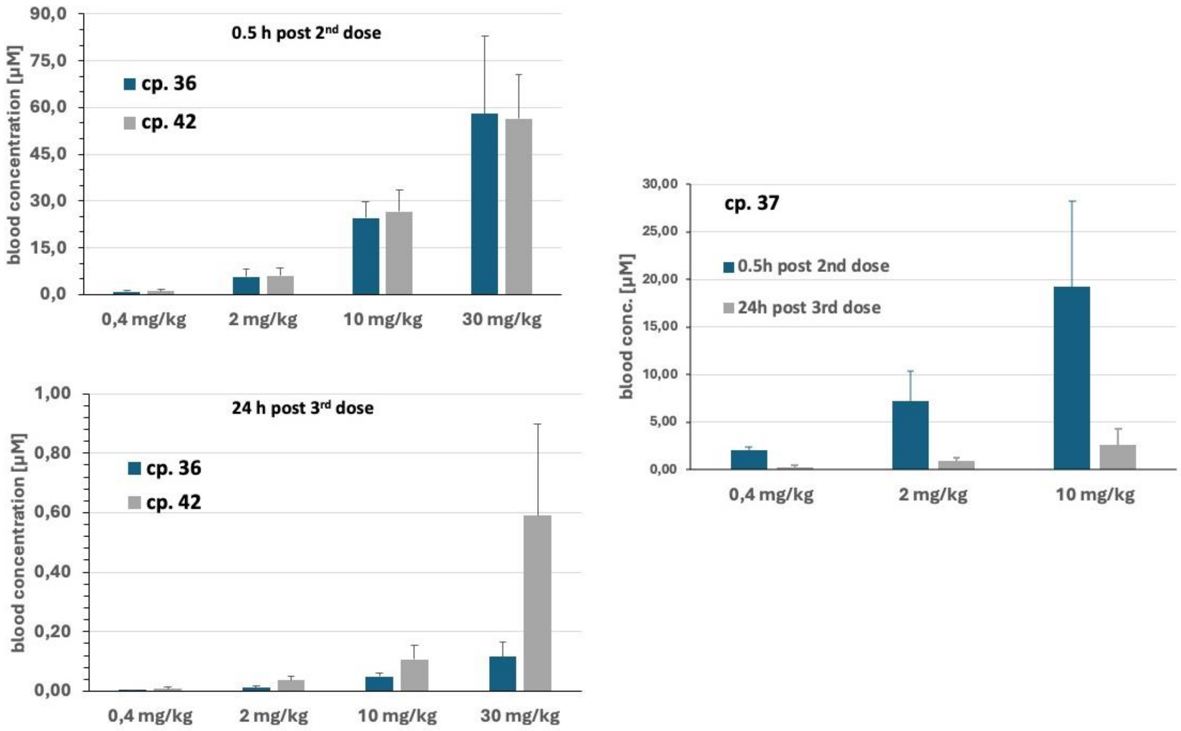

Figure S 7: Blood concentrations of MKK4-inhibitors. Test compounds were administered 12 h and 1h prior to and 24 h after induction of liver injury. Whole blood samples were collected 0.5h after the 2nd dose and 24h after the 3rd dose.

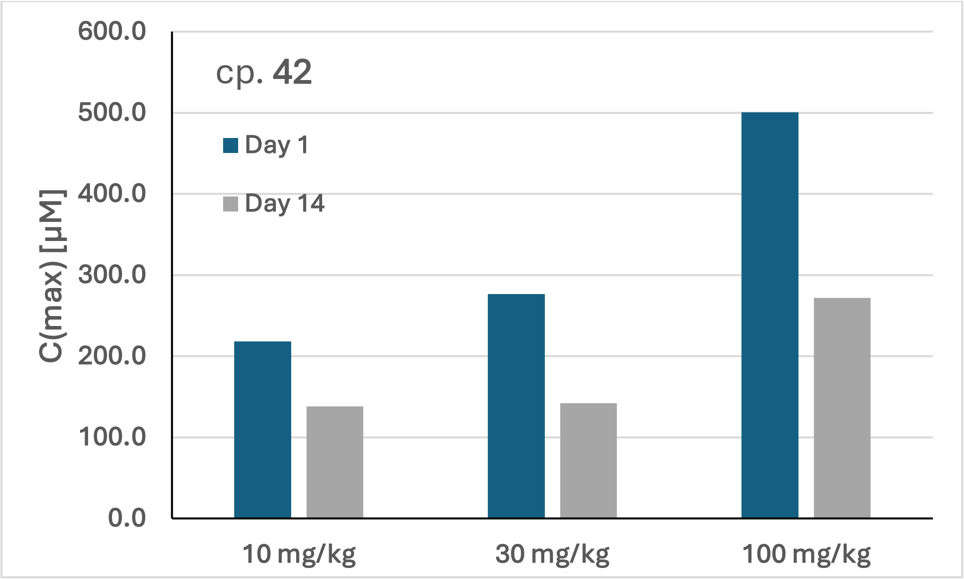

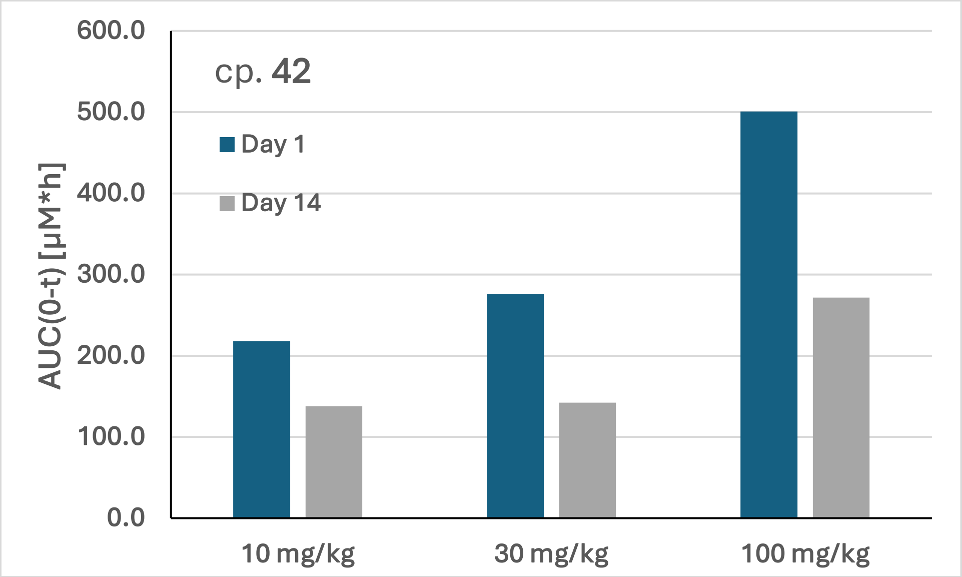

*Figure S 8: Compound **42** – pharmacokinetic parameters, determined on day 1 and day 14 in Wistar during repeated administration of compound 42 at doses of 10, 30 and 100 mg/kg/day.*

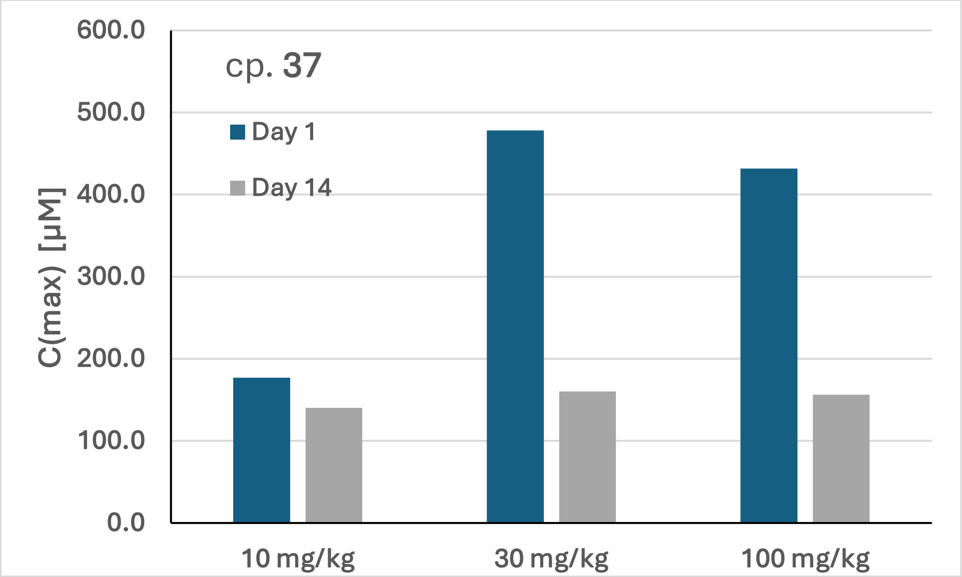

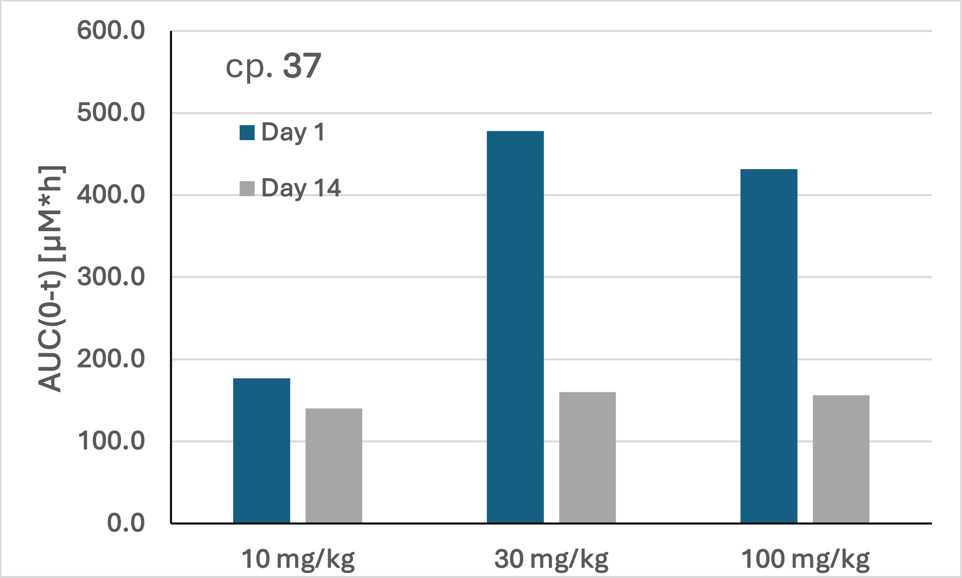

*Figure S 9: Compound **37** – pharmacokinetic parameters, determined on day 1 and day 14 in Wistar during repeated administration of compound 37 at doses of 10, 30 and 100 mg/kg/day.*

VI: References

[S1] Fabian MA, Biggs WH 3rd, Treiber DK, Atteridge CE, Azimioara MD, Benedetti MG, Carter TA, Ciceri P, Edeen PT, Floyd M, Ford JM, Galvin M, Gerlach JL, Grotzfeld RM, Herrgard S, Insko DE, Insko MA, Lai AG, Lélias JM, Mehta SA, Milanov ZV, Velasco AM, Wodicka LM, Patel HK, Zarrinkar PP, Lockhart DJ. A small molecule-kinase interaction map for clinical kinase inhibitors. Nat Biotechnol. 2005 Mar;23(3):329-36. doi: 10.1038/nbt1068. Epub 2005 Feb 13. PMID: 15711537.

[S2] Karaman MW, Herrgard S, Treiber DK, Gallant P, Atteridge CE, Campbell BT, Chan KW, Ciceri P, Davis MI, Edeen PT, Faraoni R, Floyd M, Hunt JP, Lockhart DJ, Milanov ZV, Morrison MJ, Pallares G, Patel HK, Pritchard S, Wodicka LM, Zarrinkar PP. A quantitative analysis of kinase inhibitor selectivity. Nat Biotechnol. 2008 Jan;26(1):127-32. doi: 10.1038/nbt1358. PMID: 18183025.

[S3] Nevzorova YA, Tolba R, Trautwein C, Liedtke C. Partial hepatectomy in mice. Lab Anim. 2015 Apr;49(1 Suppl):81-8. doi: 10.1177/0023677215572000. PMID: 25835741.

[S4] Mitchell, C., and Willenbring, H. (2008). A reproducible and well-tolerated method for 2/3 partial hepatectomy in mice. Nat Protoc *3*, 1167-1170.

[S5] Kloevekorn, Philip; Pfaffenrot, Bent; Juchum, Michael; Selig, Roland ; Albrecht, Wolfgang; Zender, Lars; Laufer, Stefan A. From off-to on-target: New BRAF-inhibitor-template-derived compounds selectively targeting mitogen activated protein kinase kinase 4 (MKK4). *European Journal of Medicinal Chemistry* **2021**, *210*, 112963.

[S6] Pfaffenrot, Bent; Kloevekorn, Philip; Juchum, Michael; Selig, Roland ; Albrecht, Wolfgang ; Zender, Lars; Laufer, Stefan A. Design and synthesis of 1*H*-pyrazolo[3,4-*b*]pyridines targeting mitogen-activated protein kinase kinase 4 (MKK4) - A promising target for liver regeneration. *European Journal of Medicinal Chemistry* **2021**, *218*, 113371.

[S7] Juchum, Michael; Pfaffenrot, Bent; Kloevekorn, Philip; Selig, Roland; Albrecht, Wolfgang; Zender, Lars; Laufer, Stefan A. Scaffold modified Vemurafenib analogues as highly selective mitogen. activated protein kinase kinase 4 (MKK4) inhibitors. *European Journal of Medicinal Chemistry* **2022**, 240, 114584.

[S8] Preparation of 1*H*-pyrrolo[2,3-*b*]pyridines as selective MKK4 kinase inhibitors for promoting liver regeneration or reducing or preventing hepatocyte death, WO2018134254

[S9] Preparation of substituted phenyl(1H-pyrrolo[2,3-b]pyridin-3-yl)methanones as protein kinase inhibitors for promoting liver regeneration or reducing or preventing hepatocyte death, WO2020016243

[S10] Preparation of pyrazolopyridines as protein kinase MKK4 inhibitors for promoting liver regeneration or reducing or preventing hepatocyte death, WO2019149738

[S11] N-[3-(1H-Pyrrolo[2,3-b]pyridine-3-carbonyl)phenyl]sulfonamide derivatives as protein kinase inhibitors and their preparation and use for the treatment of diseases, WO2010129570

[S12] Pyrimido [4,5-b] indole derivatives, WO2003037898

[S13] Preparation of 4-phenyl-pyrimido[4,5-b]indoles as inhibitors of MKK7, MKK4 and treatment of related diseases, WO2004058764

[S14] Heteroaryl-substituted pyrazolo-pyridines as protein kinase inhibitors for promoting liver regeneration or reducing or preventing hepatocyte death and their preparation, WO2021018820
